# Society of Surgical Oncology Consensus Statement: Assessing the Evidence for and Utility of Gene Expression Profiling of Primary Cutaneous Melanoma

**DOI:** 10.1245/s10434-024-16379-2

**Published:** 2024-10-29

**Authors:** Edmund K. Bartlett, Cristina O’Donoghue, Genevieve Boland, Tawnya Bowles, Keith A. Delman, Tina J. Hieken, Marc Moncrieff, Sandra Wong, Richard L. White, Giorgos Karakousis, Society of Surgical Oncology Gene Expression Profiling Consensus Statement Work Group

**Affiliations:** 1 Memorial Sloan Kettering Cancer Center, New York, NY USA; 2 University of Chicago, Chicago, IL USA; 3 Massachusetts General Hospital, Boston, MA USA; 4 Intermountain Medical Center, Murray, UT USA; 5 Emory Winship Cancer Institute, Atlanta, GA USA; 6 Mayo Clinic, Rochester, MN USA; 7 Norfolk and Norwich University Hospital, Norwich, UK; 8 Emory University School of Medicine, Atlanta, GA USA; 9 Atrium Health, Levine Cancer Institute, Charlotte, NC USA; 10 Hospital of the University of Pennsylvania, University of Pennsylvania Abramson Cancer Center, Philadelphia, PA USA

## Abstract

**Introduction:**

Gene expression profiling (GEP) of primary cutaneous melanoma aims to offer prognostic and predictive information to guide clinical care. Despite limited evidence of clinical utility, these tests are increasingly incorporated into clinical care.

**Methods:**

A panel of melanoma experts from the Society of Surgical Oncology convened to develop recommendations regarding the use of GEP to guide management of patients with melanoma. The use of currently available GEP tests were evaluated in three clinical scenarios: (1) the utility in patient selection for sentinel lymph node biopsy; (2) the utility to guide surveillance; and (3) the utility to inform adjuvant therapy. As a basis for these recommendations, the panel performed a systematic review of the literature, including articles published from January 2012 until August 2023.

**Results:**

After review of 137 articles, 50 met the inclusion criteria. These articles included evidence related to three available GEP tests: 31-GEP, CP-GEP, and 11-GEP. The consensus recommendations were finalized using a modified Delphi process. The panel found that current evidence often fails to account for known clinicopathologic risk factors and lacks high-level data. The panel recognizes that the study of GEP tests is still evolving. The integration of GEP into routine clinical practice for predicting sentinel lymph node status and patient prognosis in melanoma is therefore not currently recommended.

**Conclusion:**

At present, GEP should be considered primarily an investigational tool, ideally used in the context of clinical trials or specialized research settings.

**Supplementary Information:**

The online version contains supplementary material available at 10.1245/s10434-024-16379-2.

## Synopsis of Recommendations

*Full recommendations, evidentiary support, and discussion can be found in the text.*

**Question 1:** In adult patients with American Joint Committee on Cancer (AJCC) pT1a-pT4b primary cutaneous melanoma, does GEP testing improve patient selection and decision making for sentinel lymph node biopsy (SLNB) as compared with the use of conventional clinical and pathologic factors alone?

**Table:** 

*Recommendation 1.1:* GEP testing is not currently recommended for routine use in predicting sentinel lymph node (SLN) status. There is a lack of high-level evidence regarding changing indications for SLNB based on GEP results.
* Recommendation 1.1.a:* There is currently a lack of high-level evidence that GEP testing improves selection above clinicopathologic factors for SLNB in patients with AJCC pT1a primary cutaneous melanoma.
*Recommendation 1.1.b:* High-quality evidence (including prospective, adequately powered studies with independent validation) is desired to assess the role for GEP testing in guiding selection for SLNB in patients with AJCC pT1b-T2 primary cutaneous melanoma.
*Recommendation 1.1.c:* There is currently a lack of high-level evidence that GEP testing improves selection for SLNB in patients with AJCC pT3-T4 primary cutaneous melanoma.

**Question 2:** Does GEP testing improve current risk stratification of adult patients with AJCC pT1a-pT4b primary cutaneous melanoma sufficiently to recommend its utilization to guide decision making for surveillance imaging and follow-up?

**Table:** 

*Recommendation 2.1:* GEP testing is not currently recommended to guide a specific surveillance or follow-up approach in melanoma care.
*Recommendation 2.1.a:* GEP testing is not recommended to guide surveillance strategy or follow-up in patients with AJCC pT1a (clinical stage IA) melanoma who have an otherwise excellent prognosis.
*Recommendation 2.2:* EP testing is not currently recommended to replace SLNB for prognostication or staging, or to guide surveillance and adjuvant treatment approaches in patients (AJCC pT1b-pT4b) who are otherwise recommended for the procedure.

**Question 3:** In adult patients with primary cutaneous melanoma, does GEP testing provide additional information and improve risk stratification, beyond current diagnostic standards, to influence decisions for the utilization and utility of adjuvant therapy?

**Table:** 

*Recommendation 3.1:* There is currently a lack of evidence supporting the use of GEP testing to inform treatment decisions for the utilization or the utility of adjuvant therapy.	

## Future Research Directions

What further research is needed to inform indications for GEP testing in the clinical care of patients with AJCC pT1a-pT4b (cN0M0) primary cutaneous melanoma?

**Table:** 

*Recommendation:* Prospective, adequately powered studies with independent validation are needed.
*Recommendation:* Studies of patients with stage II melanoma specifically could inform treatment strategies and surveillance approaches. Recommended study designs would include use of archived tissue and prospective, adequately powered studies with independent validation.
*Recommendation:* High-quality studies comparing efficacy across GEP test platforms, nomograms, and existing clinical guidelines are needed.

The Society of Surgical Oncology (SSO) is dedicated to advancing and promoting the science and treatment of cancer. The SSO pursues this, in part, through the development of consensus statements on clinical care issues and procedures. Consensus statements and expert opinion documents are pursued when evidence is limited or there are surgical management issues and/or controversy relative to the management of disease.

## Background

Melanoma is the fifth most common cancer diagnosis in the United States, with 100,640 cases estimated to be diagnosed in 2024.^1^ Gene expression profiling (GEP) of primary cutaneous melanoma (CM) aims to offer prognostic and predictive information to guide clinical care. GEP testing is performed by using quantitative reverse transcriptase polymerase chain reaction (RT-PCR) technology to measure the messenger RNA (mRNA) expression of a panel of genes extracted from formalin-fixed paraffin-embedded primary melanoma tumor tissue slides.^2^ Current guidelines from the National Comprehensive Cancer Network (NCCN) do not recommend GEP testing to guide clinical decision making outside clinical trials until further prospective studies are performed in large, contemporary datasets of unselected patients.^3^ Similarly, the American Academy of Dermatology (AAD) currently discourages routine GEP for management decisions or prognostication outside of a clinical study or trial.^4,5^ Currently, the commercially available GEP assays marketed to aid in post biopsy clinical decision making or prognosis of melanoma include DecisionDx-Melanoma (31-GEP, Castle Biosciences), MelaGenix (11-GEP, NeraCare), and Merlin Assay (CP-GEP, SkylineDx). In 2019, the Centers for Medicare & Medicaid Services (CMS) began covering GEP in Medicare patients for nonmetastatic cutaneous melanoma T1b and above or T1a melanomas 0.3 mm or greater with documented concern about the adequacy of microstaging.^6–9^ Later, in July 2023, the CMS issued a Local Coverage Determination (LCD) stating that there is insufficient evidence to support current GEP tests for melanoma care and considers such tests investigational.^10^

GEP is marketed to dermatologists, surgeons, and oncologists, but there is uncertainty about how clinicians should act upon the results, and the tests can be associated with significant costs. GEP tests have become commonly used in some practices in order to improve upon standard clinicopathologic data to inform patient selection for sentinel lymph node biopsy (SLNB), adjuvant therapies, and surveillance, despite AAD and NCCN recommendations to the contrary.^11^ GEP results may unnecessarily delay care or be presented to clinicians after a wide local excision has already been performed, which may further introduce uncertainty into standard melanoma treatment algorithms. The cost of GEP tests varies but can be over $7000 per test.^6^ Because GEP tests are still categorized as experimental by CMS,^7,10^ many insurers do not cover this testing and patients may be responsible for payment out-of-pocket.^6^

At present, clinicians stratify patient risk of future relapse from localized melanoma based on histopathological assessment of the primary tumor. The standard melanoma synoptic pathology report provides important prognostic information that guides care. While the report is typically multifaceted,^12^ it can be categorized into:

- tumor burden, as indicated by Breslow thickness;
- tumor behavior (also known as tumor differentiation), primarily indicated by ulceration status and mitotic rate; and
- host immune response to the tumor, as indicated by tumor-infiltrating lymphocytes (TILs) and regression.

Other high-risk characteristics, such as lymphovascular invasion, neurotropism, and microsatellitosis, serve to either refine the patient’s risk of locoregional or distant relapse and likelihood of harboring sentinel lymph node (SLN) metastasis.^3^ However, these additional characteristics are only present in a small fraction of melanomas and are strongly correlated with increasing tumor burden, presence of ulceration status, and high mitotic rates.^13–18^ More recently, genetic sequencing of tumor-derived DNA to detect *BRAF, NRAS,* and *C-KIT* mutations has become standard practice in patients with metastatic disease in an effort to determine systemic treatment options.

The most important prognostic primary tumor characteristic is Breslow thickness.^3,19^ It informs not only the patient’s overall prognosis but also the risk of SLN micrometastasis. Accordingly, thin tumors, < 0.8 mm in thickness without ulceration and/or dermal mitoses, are considered very low risk (American Joint Committee on Cancer [AJCC] pT1a). Patients with these primary tumors are not, in general, recommended for SLNB. Conversely, thick tumors, > 4 mm, or tumors > 2 mm thick with ulceration (pT3b-pT4b), have a very high risk of distant relapse, even when the SLNB is negative. Adjuvant systemic therapy is now approved and can be considered for these patients,^20^ based on reports of phase III trials.^21,22^ SLNB has been offered as a standard of care for staging primary cutaneous melanomas for over 3 decades. While there may be therapeutic utility in the procedure itself for patients with low disease burden in their SLNs,^19,23–25^ the modern rationale for offering SLNB is to identify patients with micrometastatic disease who may benefit from adjuvant systemic therapy. For clinically node-negative intermediate- and high-risk primary melanoma patients, SLN status is the single most important prognostic biomarker for distant relapse and/or melanoma death.^19^

A patient’s prognosis and stage for clinically localized melanoma is based on a combination of primary tumor factors and SLNB status. For patients with a positive SLNB, prognosis is worse for thicker tumors.^19^ At the opposite end of the risk spectrum, for patients with pT1b tumors, confirmation of a negative SLNB indicates better prognosis and they are downgraded from AJCC IB to AJCC IA.^19^ The need for an accurate biomarker to stratify patients according to risk of locoregional and/or distant recurrence of their disease has become increasingly paramount with the advent of adjuvant systemic therapy for high-risk melanoma patients. Unfortunately, there is currently no blood-based biomarker for routine clinical use to screen patients at diagnosis and to allow serial surveillance of patients undergoing treatment or follow-up, although this is an area of active investigation (DETECTION trial, NCT04901988). Similarly, radiological cross-sectional imaging does not have the required sensitivity (i.e., there is a high false-negative rate) to accurately stratify clinically N0M0 primary cutaneous melanoma patients at diagnosis.^6^

There are several areas where GEP tests may hold the potential to offer information in addition to conventional clinicopathologic features for patients with AJCC pT1a-pT4b primary cutaneous melanoma. These include improving patient selection and decision making for SLNB, guiding decision making for surveillance imaging and follow-up, and improving decision making for the use and utility of adjuvant therapy. However, the ability of existing GEP assays to discern information beyond current clinicopathologic prognostication continues to evolve.

## Objective

A panel of melanoma experts from the Society of Surgical Oncology (SSO) was convened to develop consensus recommendations regarding the routine clinical use of GEP to guide the management of patients with primary cutaneous melanoma.

Current commercially available GEP tests aim to provide improved risk prediction regarding two related, but distinct, clinical scenarios: (1) prediction of the risk of a positive SLN at the time of diagnosis;^26,27^ and (2) prediction of the risk of recurrence after completed initial treatment.^28^ The panel aimed to reach expert consensus and provide recommendations regarding the use of GEP for each of these scenarios. In both scenarios, the recommendations are based on the assessment of value that GEP testing adds to currently available clinicopathologic risk factors.

Regarding the risk of a positive SLN, an ideal test would identify all patients with a positive SLN while correctly identifying at least a subset of patients without metastatic nodal disease who could be spared the procedure. The 2018 SSO/American Society of Clinical Oncology (ASCO) guidelines currently recommend SLNB for all patients with intermediate thickness melanoma, and consideration for the procedure in select patients with thin (T1b) or thick melanomas.^29^ This is based on a threshold for recommending the procedure of approximately a 10% risk of nodal positivity, with a 5% risk justifying consideration. The expert panel sought to determine whether there is sufficient evidence to suggest that treatment decisions (e.g., recommendation for SLNB) based on GEP testing would provide clinical benefit to patients compared with the current clinicopathologic-based guidelines.

Assessing the clinical impact of improved estimation of recurrence risk is complex. There are numerous potential iterations of clinical follow-up and treatment that might be varied to change management based on modest changes in predicted risk. These include the frequency of clinical examinations, the modality and frequency of imaging, and the recommendation for adjuvant therapy. Although there is little evidence that surveillance regimen impacts survival,^30^ follow-up recommendations are based on the principle that patients at increased risk of recurrence warrant more intensive follow-up. The panel decided to specifically assess the clinical utility of GEP testing for informing two clinical decisions: (1) the recommendation for cross-sectional imaging as opposed to clinical follow-up alone; and (2) a recommendation for adjuvant therapy following complete surgical treatment.

Finally, the panel also sought to provide guidance to clinicians faced with interpreting a GEP test result, even when obtained outside of recommended criteria. As many of these tests are ordered prior to surgical consultation, how this information can and should be incorporated into follow-up was evaluated.

### Intended Users

- Surgical oncologists and proceduralists performing excisions for melanoma and SLNB procedures.
- Oncologists involved in the care of melanoma patients.
- Dermatologists involved in the care of melanoma patients.

### Patient Population

The patient population for these recommendations is adults 18 years of age or older receiving or being considered for the following GEP tests:

- 31-GEP
- CP-GEP (8-gene)
- 11-GEP

## Methods

### Definitions

*Gene expression profiling:* GEP measures the relative expression of a finite panel of genes using mRNA extracted from tumor tissue.^2^ These tests have been purported to offer prognostic and predictive information to guide clinical care.

*Primary cutaneous melanoma:* Localized (T1a-4b, clinical N0, clinical M0) cutaneous melanoma, as defined by the AJCC Cancer Staging Manual, 8th edition.^19^

### Topic Selection

SSO Disease Site Work Groups (DSWGs) are made up of experts in the fields of breast, melanoma, sarcoma, gastrointestinal, colorectal, peritoneal surface malignancy, endocrine, and hepato-pancreato-biliary cancers. When identifying topics for consensus statement development, SSO DSWGs (who may collaborate with other SSO committees) are solicited for topic recommendations. Topics must be relevant to the SSO membership and meet one or more of the following criteria:

Address a disease site or clinical scenario within surgical oncology where:

- evidence is limited, inconsistent, indirect, or of poor quality;
- optimal surgical management has yet to be defined;
- there is controversy relative to management of the disease.

Topics are reviewed and prioritized by the SSO Quality Committee and submitted to the Executive Committee for final selection. Additional topics may be undertaken when there are rapid changes or advances in the cancer field.

### Expert Panel Selection

Expert panel members were appointed based on recommendations from the Melanoma DSWG and SSO leadership. The panel was approved by the SSO Executive Committee. The panel added one additional expert with experience related to consensus recommendations in this area, and a community practice surgeon. Expert panel representation included academic and community practice surgeons, as well as international representation.

### Conflicts of Interest Disclosures

All expert panel members, consensus voting group members, and peer reviewers completed disclosures in accordance with SSO policy (see the Appendix for disclosures)*.*

Chairs and Vice-Chairs may not have financial relationships with affected companies and must be free of these relationships for 6 months prior to appointment, and remain free of interests/relationships at all times during the panel’s work and through 1 year after publication. The remainder of the panel is ideally free of financial relationships with affected companies; however, it is acceptable to allow participation by a minority of members (49% or less) with permitted relationships in compliance with SSO policy. Affected companies are commercial entities with products affected by a statement for the purposes of conflict-of-interest review.

### Clinical Question Development

The expert panel Chairs identified four broad categories related to GEP testing that were shared with the panel for multiple rounds of review and comment. The panel considered standardized definitions, use of the PICOT (population, intervention, comparison, outcome, and time) method, and inclusion/exclusion criteria, resulting in the following questions that were the basis for the literature search.

- In adult patients with AJCC pT1a-pT4b primary cutaneous melanoma, does GEP testing improve patient selection and decision making for SLNB as compared with the use of conventional clinical and pathologic factors alone?
- Does GEP testing improve current risk stratification of adult patients with AJCC pT1a-pT4b primary cutaneous melanoma sufficiently to recommend its utilization to guide decision making for surveillance imaging and follow-up?
- In adult patients with primary cutaneous melanoma, does GEP testing provide additional information and improve risk stratification, beyond current diagnostic standards, to influence decisions for the utilization and utility of adjuvant therapy?
- Future Directions for Research: What further research is needed to inform indications for GEP testing in the clinical care of patients with AJCC pT1a-pT4b (cN0M0) primary cutaneous melanoma?

### Literature Search and Evidence Review

The panel used the SSO literature search protocol to ensure that literature searches were thorough, relevant, and based on a uniform strategy. Search parameters were drafted by one of the Chairs and a panel member, and included the search strategy from Marchetti et al.^28^

The draft search parameters were reviewed by the full panel. A contracted medical librarian conducted literature searches for the time periods 1 January 2012–1 September 2022 and 2 September 2022–7 August 2023 (see the Appendix for protocol and search strategies).

Expert panel members reviewed search results for relevant articles and suggested additional articles for review, including a CMS LCD.^10^ Selected articles were retrieved and assigned to panel members for evidentiary review. The panel reviewed 137 articles using the SSO-designated levels of evidence and evidentiary tables, resulting in the inclusion of 50 articles (see tables in the Appendix). A minimum of two reviewers were assigned per article. Assignments were made ensuring that no panel member reviewed any article in which they were a co-author or that listed funding from a company in which they had a relationship, or authors were from an affected company the member was affiliated with (within the allotted disclosure timeframe for each reviewer).

Publications excluded from evidentiary table review included abstracts, non-English-language publications, articles not published in a peer-reviewed journal, single case reports, and papers not specifically dealing with a commercially available GEP test. Editorials, reviews, letters to the editor, and commentaries were considered for referencing in the text if relevant but were not reviewed as part of the evidentiary support.

An arbitrator panel made up of the Expert Panel Chairs reconciled any discrepancies between reviewers in the evidentiary tables. For purposes of consistency, any studies with prospectively collected data, even if the study was conducted retrospectively, were classified as a prospective cohort study.

A summary of the evidence was generated for each question.

### Consensus Recommendation Development: Modified Delphi Process

A consensus voting panel of 20 was appointed, composed of the Expert Panel and a Consensus Voting Group. The consensus voting panel was selected keeping in mind specialty, practice setting and type (both academic and community surgeons), geographic diversity, and representation of a variety of viewpoints in the field.

Consensus recommendations were drafted by the expert panel members assigned to the evidentiary statement for each question. Each group only drafted recommendations related to their question to minimize any group bias in development and voting. Recommendations were informed by the evidence review for the related question and considerations of population, intervention, comparators, and outcome.

The full consensus voting panel voted anonymously, electronically, on each recommendation using a five-point Likert scale, with the ability to comment on the recommendations and evidentiary support and to abstain when necessary. Participants were expected to abstain if they had a conflict with an affected company or were unable to answer. Comments were mandatory if a participant disagreed or abstained. Consensus on each recommendation was considered reached if it received a minimum of 80% agreement (responses ‘strongly agree’ and ‘agree’). Abstentions were excluded from this calculation (see the Appendix for the SSO Modified Delphi Process).

Consensus was reached on all recommendations after one round of voting. Minor, non-substantive changes in wording were determined by two basic majority votes of the full consensus voting panel.

### Peer Review and Approval

The SSO Quality Committee and relevant DSWG(s) are responsible for peer review of all SSO consensus statements, with a minimum of two reviewers per group. Additional reviewers from outside the committee may be added for expertise or to reach the minimum number of reviewers. The SSO Quality Committee and Melanoma DSWG provided two peer reviewers from each group for this topic. The Board of Directors provides final review and approval.

### Updates

Review and any necessary updates are planned for within 3–5 years of publication.

### Results and Recommendations

**Fig. 1:**
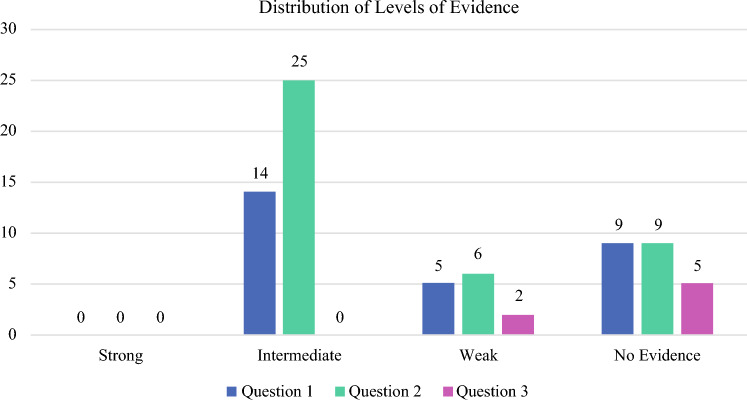
Levels of evidence for the three questions studied

Figure 1 below summarizes the levels of evidence for the three questions studied.

## Recommendations

**Question 1:** In adult patients with AJCC pT1a-pT4b primary cutaneous melanoma, does GEP testing improve patient selection and decision making for SLNB as compared with the use of conventional clinical and pathologic factors alone?

*Recommendation 1.1: *GEP testing is not currently recommended for routine use in predicting SLN status. There is a lack of high-level evidence regarding changing indications for SLNB based on GEP results.

*Recommendation 1.1.a:* There is currently a lack of high-level evidence that GEP testing improves selection above clinicopathologic factors for SLNB in patients with AJCC pT1a primary cutaneous melanoma.

*Recommendation 1.1.b:* High-quality evidence (including prospective, adequately powered studies with independent validation) is desired to assess the role for GEP testing in guiding selection for sentinel node biopsy in patients with AJCC pT1b-T2 primary cutaneous melanoma.

*Recommendation 1.1.c:* There is currently a lack of high-level evidence that GEP testing improves selection for SLNB in patients with AJCC pT3-T4 primary cutaneous melanoma.

### Evidentiary Statement

NCCN guidelines recommend that SLNB be considered in patients diagnosed with cutaneous melanoma with a ≥ 5% risk of positivity, and offered to those with a > 10% risk of positivity.^3^ Individual patient risk of SLN metastasis is currently estimated by the treating clinician based on clinicopathologic risk factors. Guidelines to assist with estimation have been published.^29^ Multiple nomograms based on clinicopathologic factors alone have additionally been developed to provide improved risk prediction, although the precise role of these tools in guiding clinical practice is undefined.^31–34^ Given the low-risk threshold for recommending SLNB, the vast majority of patients recommended to undergo the procedure will have no metastatic spread identified. In these patients, SLNB is an invasive, expensive, and potentially morbid method to provide reassurance.^35–37^ Because indications for the procedure are risk-based, improved prediction of SLN positivity could lead to a substantial reduction in the use of SLNB procedure by correctly avoiding it in patients with negative SLNs.

The systematic review identified 28 papers relevant to the use of GEP in guiding selection for SLNB. Of these, seven articles were identified as reporting novel cohorts of patients who underwent both SLNB and GEP testing with the aim of assessing the GEP predictive performance for SLNB. Five of these published studies assessed CP-GEP, one study assessed 31-GEP, and one study assessed i31-GEP. No studies directly related to SLNB prediction using the 11-GEP test were identified. There were no studies that directly compared the GEP tests against each other (Appendix Table 1).

#### CP-GEP

The clinicopathologic and gene expression (CP-GEP) test combines clinicopathologic factors of Breslow thickness and patient age with results of a composite gene-expression score from eight genes to classify patients into low-risk (< 5% SLN positivity) or high-risk. Of the five studies identified, one^26^ reported on the development of the CP-GEP test. Three of the additional studies were retrospective external validation cohorts that included a total of 839 patients. One study^38^ was prospectively performed and included an additional 260 patients. These studies almost exclusively included patients with T1b-T4 melanoma (eight total patients with T1a across all validation studies), all of whom underwent SLNB.

Across the three retrospective validation cohorts,^39–41^ assessment of the performance in T1b melanoma was limited due to small numbers. Across the three studies, a total of nine patients with positive nodes were included, four of whom had false-negative CP-GEP results. Performance in T2 melanoma appeared promising, with negative predictive value (NPV) ranging from 89 to 97% across the three studies in cohorts where the SLN positivity rate exceeded 20%. Use of the CP-GEP would result in reduction of approximately 30% of SLNBs for patients with T2 melanoma across the studies. For T3/T4 melanoma, there were very rare low-risk CP-GEP results, suggesting limited utility. Of the 341 patients with T3/T4 tumors in the three studies, five were found to have a low-risk CP-GEP result.

The results of the prospective validation study,^38^ perhaps the highest-quality data to date, support the findings of the retrospective studies, with poor performance in T1 (sensitivity 0%, albeit limited numbers) and excellent performance in T2 (sensitivity 100% in this cohort). Only two of the 74 T3/T4 patients received a low-risk CP-GEP result. There is an ongoing prospective trial assessing the ability of CP-GEP to correctly identify patients as low-risk who would otherwise be offered SLNB (MERLIN_001 Trial).^42^

#### 31-GEP

The 31-GEP test was initially developed with the aim of improving prognostication regarding the risk of recurrence. Vetto et al. assessed the use of the 31-GEP risk score in improving risk prediction for patients with SLNB.^43^ The 31-GEP risk score classified patients into risk groupings (Classes 1A, 1B, 2A, 2B), with 1A representing the lowest risk and 2B representing the highest risk. The study proposed that patients with a combination of older age (> 65 years) and a 1A 31-GEP result could safely forgo SLNB as their risk of SLN positivity was below 5%. This study had numerous methodologic limitations, including the fact that patients over 65 years of age in the study had a baseline SLN positivity rate < 5% regardless of the 31-GEP result.

#### i31-GEP

An updated model was more recently developed specifically with the aim of improving prediction of SLN positivity. This update employed a neural network-based prediction model using clinicopathological factors (thickness, mitoses, ulceration, age) plus molecular analysis (31-GEP) and has been termed i31-GEP.^27^ This study utilized a large *(n* = 1398) training cohort to develop the model and then applied the model to an additional 1674 patients as an internal validation cohort. The model provides a continuous risk estimate for SLN positivity for the patient, and the performance characteristics were assessed assuming that < 5% risk was a low risk/negative test. The validation cohort for the study included patients with T1a-T3b melanoma.

For the study, high-risk T1a patients were defined as having one or more of the following characteristics: age < 40 years, > 1 mitoses/mm^2^, present regression, present lymphovascular invasion, transected base, and absent TILs. 31-GEP had a poor sensitivity in this population (43%) and a positive predictive value of < 5%. In patients with T1b melanoma, the sensitivity was improved (83%), with an NPV of nearly 98%. Use of i31-GEP testing to guide selection for SLNB in these T1b patients would have reduced procedures by 41%. In patients with T2a melanoma, the sensitivity was high (96%) and the NPV also remained high (96%), although the reduction in procedures was less common (13%). Low-risk results were rare in T2b patients and only one low risk result was seen in 435 patients with T3/T4 melanoma. It should be noted that to date, these results have not been replicated in an external validation study. It is also worth noting that 25% of patients in the validation cohort did not actually undergo SLNB and were assumed to be node-negative.

**Question 2:** Does GEP testing improve current risk-stratification of adult patients with AJCC pT1a-pT4b primary cutaneous melanoma sufficiently to recommend its utilization to guide decision making for surveillance imaging and follow-up?

*Recommendation 2.1:* GEP testing is not currently recommended to guide a specific surveillance or follow-up approach in melanoma care.

*Recommendation 2.1.a:* GEP testing is not recommended to guide surveillance strategy or follow-up in patients with AJCC pT1a (clinical stage IA) melanoma who have an otherwise excellent prognosis.

*Recommendation 2.2:* GEP testing is not currently recommended to replace SLNB for prognostication or staging, or to guide surveillance and adjuvant treatment approaches in patients (AJCC pT1b-pT4b) who are otherwise recommended for the procedure.

### Evidentiary Statement

Risk stratification for patients with newly diagnosed localized melanoma (pT1-pT4b) has been, and continues to be, based on clinical and pathologic staging, which, when indicated, includes SLNB. Current guidelines from the NCCN for surveillance of patients with melanoma and consideration of adjuvant therapies are primarily based on AJCC staging.

To date, there have been no prospective, high-level evidence studies favoring one surveillance strategy over another. Generally, patients with low-risk melanoma (stage IA/IB/IIA) are recommended to have skin and lymph node examinations as the primary form of surveillance, while higher-risk patients (stage IIB or higher) are recommended to have imaging at variable frequency (every 3–12 months). Moreover, in the context of novel immune checkpoint inhibitors, the exact benefit of early detection of recurrent or metastatic disease remains to be seen. Notably, all adjuvant trials to date leading to drug approvals have been designed primarily using AJCC stage groupings for patient eligibility.

Therefore, an optimal surveillance strategy for melanoma has not been defined in the contemporary therapeutic landscape. There is no high-level evidence supporting the use of GEP assays to better define surveillance recommendations.

#### 31-GEP

The systematic review identified 18 publications relating to the 31-GEP assay and assessment of prognosis. The limitations of the body of literature evaluating 31-GEP and surveillance include the retrospective nature of most studies, as well as overlapping study cohorts across studies. Acknowledging these considerations and potential limitations, the preponderance of the data from numerous studies suggest that the 31-GEP assay can help further risk stratify patients with respect to melanoma outcomes beyond AJCC staging, including recurrence-free survival (RFS), distant metastasis-free survival (DMFS), and melanoma-specific survival (MSS).

Upon closer inspection of the multivariate analyses contained within the publications assessed, it becomes evident that the characteristics being displaced by GEP are related to ulceration status and mitotic rate, suggesting that 31-GEP primarily describes tumor differentiation as well as tumor depth.^44^ Class II GEP tumors are predominantly found in AJCC stage II (pT2b-pT4) patients, although the exact proportions vary and are inconsistently stated across studies. It is also noteworthy that a significant proportion of patients in these studies did not undergo SLNB.^31^ SLNB status remains an independent predictor of outcome when analyzed through multivariate analysis.^45^

A meta-analysis of 1479 patients found an improvement in sensitivity and NPV in DMFS when combining the 31-GEP assay with SLN status.^46^ The difference in prognosis between Class I and II results may appear more pronounced when stage I and II patients are aggregated. A separate meta-analysis including 623 stage I melanoma patients found a 3% overall recurrence rate, of which just 1% were Class II, suggesting a particularly limited role for prognostication in early-stage disease.^28^ There remains limited data in the performance accuracy of 31-GEP as compared with prognostic models utilizing the full complement of histopathologic and clinical factors in recurrence risk assessment. While there have been an increasing number of studies investigating the prognostic utility of GEP assays for SLN status (as summarized in the prior section), there remains scarce patient cohort data for GEP recurrence prognostication in patients who were otherwise recommended for SLNB who did not undergo the procedure.

Studies have shown that GEP results can influence clinician decision making^47,48^ in the care of patients with melanoma, including decision for SLNB and intensity of surveillance. However, the extent to which these changes in clinician behavior translate into improvements in patient outcomes remains undefined. One study^49^ demonstrated that routine imaging surveillance in the class II patient population that had undergone GEP testing led to the earlier identification of metastatic disease, as measured by the smaller size of metastatic index tumors compared with a control group that did not undergo testing and was not surveilled by imaging. It is unclear if there was selection bias for which patients underwent GEP testing compared with those who did not. For instance, there were many more stage I patients in the ‘control’, non-imaged group who recurred compared with the GEP-tested stage I group who were classified as class II. What proportion of those ‘control’ patients would have appropriately been classified as class II had they been tested is unclear. Most recurrences occurred in patients with T3b or higher tumors in the experimental group. These patients would routinely be getting surveillance imaging absent any GEP testing, based on AJCC staging and NCCN guidelines alone.

Finally, to what extent earlier detection of metastatic disease guided by GEP class resulted in improved long-term oncologic outcomes has not been sufficiently addressed.

One recent study^50^ using the Surveillance, Epidemiology, and End Results (SEER) database found that 31-GEP testing was associated with an improvement in MSS and overall survival. This was a retrospective study without defined selection criteria as to which patients underwent GEP testing, and recurrence data were not available. While a propensity-matched analysis was performed between GEP tested and non-tested patients, various factors (such as socioeconomic status, receipt of adjuvant therapies, *BRAF* status, receipt of treatments at recurrence) were not adjusted for in the analysis.

#### CP-GEP

The CP-GEP test was initially designed for the prediction of SLN positivity at the time of SLNB (see section above). The systematic review identified two retrospective studies of patients with stage I/II melanoma within the study period,^51,52^ which identified an association with a high-risk CP-GEP test and increased risk of recurrence. The authors also noted the recently presented MELARISK-001 study summarized further in the Discussion section.^53^ Further prospective studies are needed to warrant a change in clinical practice. Long-term follow-up of the ongoing MERLIN_001 trial may provide prospective data to inform the prognostic value in the future.

#### 11-GEP

The systematic review identified a single study on the prognostic value of the 11-GEP test. This study specifically examined the prognostic value of the test in a retrospective cohort of 246 patients with stage II melanoma. The study identified a significant association with the 11-GEP result and both RFS and disease-specific survival.^54^

**Question 3:** In adult patients with primary cutaneous melanoma, does GEP testing provide additional information and improve risk stratification, beyond current diagnostic standards, to influence decisions for the utilization and utility of adjuvant therapy?

*Recommendation 3.1:* There is currently a lack of evidence supporting the use of GEP testing to inform treatment decisions for the utilization or utility of adjuvant therapy.

### Evidentiary Statement

The eligibility of patients with primary cutaneous melanoma to receive adjuvant treatment is based on guidelines from the NCCN using AJCC pathologic staging for patients with stage IIB or greater melanoma. Patient eligibility for adjuvant treatments is based on AJCC pathologic stage and no trials leading to drug approval for adjuvant treatment have used GEP testing. Stage IIB and IIC melanomas are known to be high risk for disease progression based on clinicopathologic factors, and US FDA approval for adjuvant anti-programmed death-1 (PD-1) therapy was granted in 2021 as a result of the results of the KEYNOTE-716 trial showing a reduction in the risk of disease recurrence versus placebo.^21^ GEP testing has the potential to both identify high-risk patients for developing recurrence or metastasis who would benefit from the addition of adjuvant therapy, as well as decrease the overuse of adjuvant treatment by improving the selectivity of patients who are eligible according to AJCC staging and may be overtreated. Some studies assessing prognostic value (prior section) did so with the intention of providing data to guide such an adjuvant therapy trial. An example of using GEP testing to direct adjuvant therapies is a study of Swedish and Dutch cohorts that used the CP-GEP model to stratify patients into two groups differentiated by RFS. This study found that the model is not yet appropriate for clinical practice because the false positive rate is too high and would lead to overtreatment if used to guide adjuvant therapy.^52^ No studies of any GEP assay have been completed using the GEP results to prospectively guide adjuvant treatment. Current evidence is insufficient to change clinical practice to incorporate GEP testing to influence adjuvant treatment decisions.

Ongoing research evaluating whether GEP testing can provide additional information includes the NivoMela trial. This prospective, randomized, phase III trial is evaluating whether stage II patients who had surgery benefit from screening with the 11-GEP assay, and, if deemed high-risk, treatment with adjuvant nivolumab versus placebo. The study is projected to end in 2028 (NCT04309409).^55^

### Future Directions for Research: What Further Research is Needed to Inform Indications for GEP Testing in the Clinical Care of Patients with American Joint Committee on Cancer (AJCC) pT1a-pT4b (cNOMO) Primary Cutaneous Melanoma?

*Recommendation:* Prospective, adequately powered studies with independent validation are needed.

*Recommendation:* Studies of patients with stage II melanoma specifically could inform treatment strategies and surveillance approaches. Recommended study designs would include the use of archived tissue and prospective, adequately powered studies with independent validation.

*Recommendation:* High-quality studies comparing efficacy across GEP test platforms, nomograms, and existing clinical guidelines are needed.

### Evidentiary Statement

Further research is needed if GEP tests are to be incorporated into the treatment of primary cutaneous melanoma and thus change the standard of care for the determination of SLNB use, surveillance, and adjuvant treatments. Prospective, randomized clinical trials that are large enough to be powered to answer such questions are needed to acquire the data to change practice. Two ongoing trials may potentially inform the use of CP-GEP to select patients for SLNB and 11-GEP to guide adjuvant therapy (NCT04759781, NCT04309409).^42,55^ The results of these studies are awaited. As an alternative to large, prospective trials, the Melanoma Prevention Working Group (MPWG) recommended utilizing archived primary tumors from stage II patients and conducting retrospective studies from placebo-controlled trials of anti-PD1 to support their clinical utility before moving forward with costly prospective trials.^56^

## Discussion

The recommendations developed by the panel of melanoma experts from the SSO found that the role of currently available GEP tests for SLNB patient selection, surveillance, and utilization of adjuvant therapies is still investigational and should not be routinely used. This is the most comprehensive review to date of the evidence with 50 studies and supports the analysis of other organizations, including the NCCN, AAD, MPWG and CMS.^3,5,10,56^

The challenge for diagnostic biomarkers obtained when the primary tumor and SLNs are excised is that the metastatic process in melanoma is somewhat stochastic,^57,58^ meaning that both synoptic reports and GEP provide risk assessments on a population level rather than for individual patients. This stochastic nature implies that while these tools are useful for understanding and categorizing risks, they are not definitive predictors of individual outcomes. For GEP of primary tumors to afford clinical utility, especially offering the patient an SLNB, the testing needs to provide not only prognostic significance independent of the synoptic report but also prognostic value that is sufficient to lead to a change in management. The preponderance of evidence suggests that the existing GEP tests do add some degree of independent prognostic value. What has yet to be shown in any prospective study with high-quality data is that this additional prognostic value leads to improved clinical outcomes. There is potential for GEP to amplify current histopathologic information to inform patient care, but more work needs to be done to understand the appropriate GEP, patient population, and cost-effectiveness of such assays. The panel looks forward to the results of the MERLIN_001 trial assessing the ability of CP-GEP to identify patients as low-risk who may forgo an SLNB, as well as the results of the NivoMela trial, which may help inform decisions regarding adjuvant immunotherapy.^42,55^ The recently presented MELARISK-001 CP-GEP high-risk stage IB/IIA patients had a 10-year RFS of 72% and MSS of 82%, which is comparable with higher-stage melanoma patients who are candidates for adjuvant therapy and could inform adjuvant patient selection. The study tested 382 node-negative IB/IIA patients and classified 212/382 (55.4%) as high-risk.^53^ The high-risk group demonstrated poorer RFS and MSS but it remains unclear if the effect is sufficient to change management.^53^ Results of these studies and other future well-designed studies may lead to modifications of recommendations.

A substantive portion of the research included in the systematic review supporting GEP in melanoma is not only industry-sponsored but also industry-authored. This raised significant concerns about potential conflicts of interest. Furthermore, there were concerns regarding the quality of the analyses that were undertaken in some of the publications. It was also notable that there was evidence to suggest repeated publishing of datasets.^44^ This consensus statement included industry-sponsored studies that were published in peer-reviewed journals, and the funding source of each study is listed in the evidentiary tables found in the Appendix. The majority of the GEP studies included in this review featured relatively short follow-up durations, typically around 3 years or less, which might not have adequately captured the long-term outcomes and the recurrence patterns of melanoma. Independent studies demonstrating improved outcomes of patients receiving GEP compared with those treated by current standards with longer follow-up periods are crucial to ascertain the true value of GEP in melanoma prognosis and treatment.

The potential harms associated with incorrect risk classification have not been well-studied. Based on GEP test results, patients may inappropriately forgo an SLNB and potentially omit adjuvant systemic treatment that would have otherwise been recommended. Additionally, patients who would otherwise be considered low risk may have anxiety associated with a high score and increased surveillance. Increasing the frequency of surveillance imaging in early-stage melanoma when a clear outcome benefit has not been defined may increase risk to patients.^59^ Imaging is costly and can add to the financial burden of melanoma treatment. High-deductible insurance plans are becoming more common and can leave patients with high out-of-pocket costs for imaging. Insurance carriers may limit the total number of scans a patient can receive over time, particularly in the setting of surveillance. Routine imaging can also reveal incidental findings that are not clinically significant but can prompt diagnostic procedures that carry risk of harm. The radiation exposure of frequent computed tomography (CT) scan imaging and the link to increased risk of other cancers, especially in young patients, also needs to be considered. Patient education on skin and lymph node appearance of melanoma recurrence and routine skin examinations by a skin cancer expert are important in early-stage melanoma and are not necessarily inferior to imaging in the detection of recurrence.^60^ Lastly, if future studies demonstrate the validity of GEP tests, the cost effectiveness of GEP should be studied to evaluate the benefits, if any, in patient outcomes with the costs of the individual tests and downstream surveillance.

The use of GEP is common, with an estimated 5–10% of biopsied melanomas tested,^2^ and with many dermatologists reporting having ordered the 31-GEP test.^61^ GEP testing should be discussed with the multidisciplinary team of clinicians caring for patients, or in a trial setting, as surgeons and oncologists often receive results that may not impact treatment recommendations yet cause patient distress and confusion. Given the complexities and uncertainties surrounding GEP testing, patient education and informed consent become paramount. Patients should be fully informed about the potential benefits and limitations of GEP testing, as well as the current state of research in this area. This approach ensures that patients can make informed decisions about their care and have realistic expectations regarding the outcomes of GEP testing.

## Conclusion

GEP of melanomas holds immense promise. This tool has the potential to, in the future, provide crucial information to clinicians and patients to improve and better individualize treatment recommendations. Some authors of this consensus statement have been involved in GEP research since its introduction to the medical community nearly a decade ago. However, as with any new clinical tool, it is essential to balance enthusiasm for its potential with the need for rigorous scientific validation. This balance can be challenging, especially since scientific research often requires financial partnerships with industry.

**Fig. 2:**
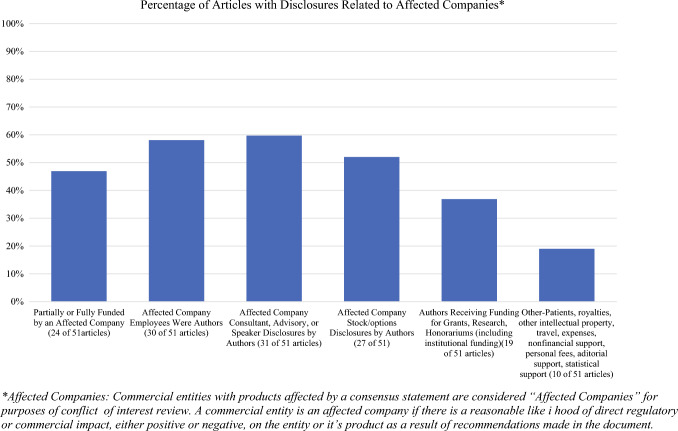
Distribution of disclosure types related to affected companies

Industry faces a dual obligation: to serve the needs of patients and clinicians, and to satisfy investors and corporate financiers, whose support is necessary for continued innovation. The studies reviewed by the panel reflect these tensions. The panel noted that nearly half of the studies evaluated were partially or fully funded by industry. Over half of the studies evaluated had industry employees listed as authors. Most articles also listed disclosures with one or more of the following: authors who were industry consultants, advisors, speakers, owned industry stock/options, or were receiving industry funding for grants, research or honoraria (see Fig. 2)*.* Many of the studies reviewed also involved overlapping patient populations. While these relationships do not necessarily indicate bias, they warrant increased scrutiny of study results.

While GEP could potentially offer objective and valuable insight into the risk profile of a given primary melanoma, its role in predicting SLNB status and overall prognosis is still evolving. The integration of GEP into routine clinical practice for predicting SLN status and patient prognosis in melanoma is therefore premature. At present, GEP should be considered an investigational tool, used primarily in the context of clinical trials or specialized research settings. Well-established clinicopathologic factors continue to be the cornerstone of melanoma prognosis and should remain the primary basis for clinical decision making at this time.

## Supplementary Information

Below is the link to the electronic supplementary material.

Supplementary file1 (PDF 631 kb)

Supplementary file2 (PDF 1497 kb)

## Comments

Comments on this document may be sent to the SSO for consideration in future iterations of the work.
