# Supplementary material for: Society of Surgical Oncology Consensus Statement: Assessing the Evidence for and Utility of Gene Expression Profiling of Primary Cutaneous Melanoma
Source: Ann Surg Oncol. 2024 Oct 29;32(3):1429–42. doi: 10.1245/s10434-024-16379-2 (PMC11811439; doi:10.1245/s10434-024-16379-2)
Supplement: Supplementary file 1 — Supplementary file1 (PDF 631 kb) [file 10434_2024_16379_MOESM1_ESM.pdf]

SSO SOCIETY OF SURGICAL ONCOLOGY LEADING. TOGETHER.

Appendix:

Assessing the Evidence for and Utility of Gene Expression Profiling of Primary Cutaneous Melanoma

Contents

Abbreviations 2
Expert Panel 3
Consensus Panel 3
Peer Review 4
Disclosures 5
Tables 6
Literature Search Protocol 7
Literature Search 8
Search Strategies 8
  Ovid MEDLINE 8
    Query notes 9
  Embase 9
    Query notes 10
  The Cochrane Library 11
    Query notes 11
Literature Flow Chart 15
Levels of Evidence 16
SSO Modified Delphi Process 18

Abbreviations

AAD	American Academy of Dermatology
AJCC	American Joint Commission on Cancer
ASCO	American Society of Clinical Oncology
BRAF	A gene on chromosome seven that encodes a protein that has a role in cell growth.[1]
C-KIT	Melanoma mutation derived from acral, mucosal, and chronically sun-damaged melanomas. Accounts for about 30% of melanomas.[2]
CM	Cutaneous melanoma
CMS	Centers for Medicare & Medicaid Services
CP-GEP	Clinicopathologic and gene expression
CT	Computed tomography
DNA	Deoxyribonucleic acid
DSWGs	Disease Site Work Groups
FDA	Food and Drug Administration
GEP	Gene expression profiling
LCD	Local coverage determination
MEK	Mitogen-activated extracellular signal-regulated kinase
MPWG	Melanoma Prevention Working Group
MRNA	Messenger ribonucleic acid
MSS	Melanoma-specific survival
N0M0	Using TNM staging system to describe amount and spread of cancer in the body. N0=No cancer in nearby lymph nodes. M0=Cancer hasn't spread to other body parts.[3]
NCCN	National Comprehensive Cancer Network
NPV	Negative predictive value
NRAs	Gene mutation caused melanoma that is more aggressive and associated with worse outcomes compared to non-NRAs melanomas.[4]
PICOT	Patient, Intervention, Comparison, Outcome, Time
RFS	Relapse-free survival
RT-PCR	Reverse transcriptase polymerase chain reaction
SEER	Surveillance, Epidemiology and End Results

SLN	Sentinel lymph node
SLNB	Sentinel lymph node biopsy
SN	Sentinel node
SNB	Sentinel node biopsy
SSO	Society of Surgical Oncology
TILs	Tumor infiltrating lymphocytes

Expert Panel

Giorgos C. Karakousis, MD, Co-Chair
University of Pennsylvania Abramson Cancer Center

Richard L. White, Jr., MD, Co-Chair
Atrium Health, Levine Cancer Institute, Charlotte, NC

Edmund Bartlett, MD
Memorial Sloan Kettering Cancer Center, New York, NY

Genevieve Boland, MD, PhD, FSSO
Massachusetts General Hospital, Boston, MA

Tanya Bowles, MD
Intermountain Medical Center, Murray, UT

Keith A. Delman, MD, FSSO
Emory Winship Cancer Institute, Atlanta, GA

Tina J. Hieken, MD
Mayo Clinic, Rochester, MN

Marc Moncrieff, MD
Norfolk & Norwich University Hospital, Norwich, England, United Kingdom

Cristina O'Donoghue, MD, MPH
University of Chicago, Chicago, IL

Sandra L. Wong, MD, MS, FSSO
Emory University School of Medicine, Atlanta, GA

Consensus Panel

**The consensus voting panel was made up of the expert panel and consensus panel.*

Georgia Beasley, MD, MHSc
Duke University, Durham, NC

Carlo M. Contreras, MD, FACS
The Ohio State University Comprehensive Cancer Center, Columbus, OH

Mark B. Faries, MD
The Angeles Clinic and Research Institute / Cedars Sinai Medical Center, Los Angeles, CA

Jeffrey M. Farma, MD, FACS
Fox Chase Cancer Center, Philadelphia, PA

Nasreen A. Vohra, MD, FACS, FSSO
Brody School of Medicine, East Carolina University, NC

Callisia Clarke, MD, MS, FSSO
Medical College of Wisconsin, Milwaukee, WI

Geoffrey Young MD, PhD, FACS, FSSO
Miami Cancer Institute, Herbert Wertheim College of Medicine, Florida International University, Miami, FL

Burton L. Eisenberg, MD
Hoag Family Cancer Institute, Newport Beach, CA

Kim E. Isaacs, MD
St. Vincent's Hospital Sydney, Darlinghurst, NSW, Australia

Katherine Shoush Cools, MD, MSCR
Henry Ford Hospital, Detroit, MI

Peer Review

Quality Committee
Steven L. Chen, MD, MBA, FACS; OasisMD
Karyn Stitzenberg, MD, MPH; University of North Carolina

Melanoma Disease Site Work Group
John Hyngstrom, MD; Huntsman Cancer Institute
Amanda Kirane, MD, FACS, FSSO; Stanford University School of Medicine

Disclosures

Disclosures: Assessing the Evidence for and Utility of Gene Expression Profiling of Primary Cutaneous Melanoma		
As of 8.20.24		
Affected Companies		
Affected Companies are commercial entities with products affected by a consensus statement for purposes of conflict of interest review under SSO COI policies. A commercial entity is an affected company if there is a reasonable likelihood of direct regulatory or commercial impact, either positive or negative, on the entity or its product as a result of recommendations made in the document.		
Castle BioSciences		
Skyline Dx		
MelaGenix (Neracare)		
Expert Panel Disclosures		
Name	***Disclosure***	***Management Notes***
Giorgos Karakousis, MD-Chair	Research Funding-Merck--PI of an investigator initiated trial on neoadjuvant PD-1 therapy for clinical stage II B/C melanoma 2019-present (research monies to support trial and studies going to the institution). Advisory Board for Merck; Bristo Meyers Squibb Advisory Board-no direct relationships to project.	No affected companies
Richard White, MD, Co-chair	None	
Tina Hieken, MD	Research Funding-Genentech and SkylineDx - current research funding to institution.	Recommended Management of Conflict: Acknowledging a COI in the disclosure report and restricting participation to elements where the conflict is not an issue
Sandra Wong, MD	None	
Genevieve Boland, MD	Consultant, Advisor, Research Funding. Novartis, Speaker, 2019; Takeda Oncology, Speaker, 2019; Consultant, NW Biotherapeutics, 2020; Merck, Consultant, 2021; InterVenn Biosciences, Consultant, 2021; Ankyra Therapeutics, Consultant, 2021; Iovance, Advisory Board, 2021, 2023; Nektar Therapeutics, SAB, Steering Committee, 2020; Merck, Advisory Board, 2021, 2022, 2023; Novartis, Surgical Advisory Board, 2020; Ankyra Therapeutics, SAB, 2021, 2022, 2023; Olink Proteomics, Palleon Pharmaceuticals, InterVenn Biosciences, Sponsored Research Agreements 2021. Ankyra Therapeutics, Stock, 2021; Moderna Scientific Advisory Board, June 2024.	No affected companies
Keith Delman, MD	None	
Marc Moncrieff, MD	Advisor, Honoraria, Travel Expenses, Research Funding. Recipient of a grant from the UK NIHR: NIHR grant - 19/102: 130886; Received an honorarium and travel expenses from Novartis in Canada in Dec 2019; Received an honorarium from Amgen to attend an advisory panel session - Oct 2019	
Cristina O'Donoghue, MD	None	
Edmund Bartlett, MD	Honoraria-Excite International in 2021; Research Funding-Institutional research support from Skyline Dx (Rotterdam, Netherlands and San Diego, United States) pertaining to the Merlin Assay which is ongoing. Institutional research support from Qbiotics Group (Added 7.19.23)	Recommended Management of Conflict: The COI is acknowledged in the disclosure report and known to the panel chair and others. They may participate, but panel chair should be cognizant of potential COI when assigning duties when relevant to the companies interest
Tawnya Bowles, MD	Research Funding-Genentech: 2019 to current. Principal investigator for a melanoma clinical research study. I don't receive any direct funds. Amgen 2018 to current- Principal investigator for a clinical research study. I don't receive any direct funds. Replimune 2019 to current- Principal investigator for a clinical research study. I don't receive any direct funds. Natera- 2020 to current- Principal investigator for a clinical research study. I don't receive any direct funds.	No affected companies
Consensus Voting Panel Disclosures		
Name	***Disclosure***	***Management Notes***
Georgia Beasley, MD, MHSc	Bristol Myers Squibb-Honoraria rec'd 10/10/23. Clinical Trial Funding, 2022-Present; Funding paid to institution from Delcath, Replimune, SkylineDx, Philogen	Recommended Management of Conflict: The COI is acknowledged in the disclosure report and known to the panel chair and others. They may participate, but panel chair should be cognizant of potential COI when assigning duties when relevant to the companies interest.
Callisia Clarke, MD, MS, FSSO	No financial relationships. No other interests/relationships.	
Carlo Contreras, MD	No financial relationships. No other interests/relationships.	
Katherine Cools, MD, MSCR	No financial relationships. No other interests/relationships.	
Burton Eisenberg, MD	No financial relationships. No other interests/relationships.	
Mark Faries, MD	Advisory boards-Merck, Bristol Myers Squibb, Regeneron, Delcath	No affected companies
Jeffrey Farma, MD, FACS	No financial relationships. No other interests/relationships.	
Kim Isaacs, MD	No financial relationships. No other interests/relationships.	
Nasreen Vohra, MD	No financial relationships. No other interests/relationships.	
Geoffrey Young, MD, PhD, FACS	No financial relationships except Miami Cancer Institute. No other interests/relationships.	
Peer Reviewer Disclosures		
Name	***Disclosure***	***Management Notes***

Steven L. Chen, MD, MBA, FACS	Employment (self). Impedimed, Inc-no known intersenction with the consensus document; Employment (spouse). Philips-no known intersection with the consensus document. Other-Castle Biosciences--honoraria for advisory board in March of 2022 (single event).	Outside the disclosure timeframe.
John Hyngstrom, MD	Research Funding-Sklyine Dx. I am a site PI for the MERLIN prospective study that is an observational study evaluating their CP-GEP test. Skyline provides funding to run the study. I do not receive direct funding from this support. I am also site PI for other clinical trials sponsored by BMS, Merck, Amgen, Philogen, Lyell, Takara, and Natera.	Recommended Management of Conflict: The COI is acknowledged in the disclosure report and known to the panel chair and others. They may participate, but panel chair should be cognizant of potential COI when assigning duties when relevant to the companies interest.
Amanda Kirane, MD, FACS, FSSO	No financial relationships.	
Karyn Stitzenberg, MD, MPH	Stock ownership-Merck, Johnson and Johnson, Pfizer, Viatris, Organon, Vertex, Cigna, United Healthcare, CVS Aetna.	No affected companies

Tables

Traditional clinicopathologic factors continue to be the cornerstone of melanoma prognosis and should remain the primary basis for clinical decision-making

Table 1. Summary of cohort studies reporting performance of GEP testing to select patients for SLNB.

Source	Funding	Design	Setting (Location)	Population	External Validation	Participants by AJCC T Category (clinically node negative)
**CP-GEP**						
Bellomo et al. 2020	NCI, Mayo Clinic, Philanthropy	Retrospective Cohort Study	Single academic center (USA)	Patients undergoing SLNB for 1.0-4.0 mm melanoma OR 0.75-0.99 mm thick melanoma with 1 high risk feature OR 0.5-0.74 mm thick melanoma with 2 high risk features	No	T1 = 186<br>T2 = 309<br>T3 = 131
Johansson et al. 2022	SkylineDx, Philanthropy	Retrospective Cohort Study	Single academic center (Sweden)	Consecutive patients undergoing SLNB (2006-2014)	Yes	T1 = 30<br>T2 = 210<br>T3 = 118<br>T4 = 63
Mulder et al. 2021	National Enterprise Agency Netherlands, Authorship by SkylineDx employees	Retrospective Cohort Study	Single academic center (Netherlands)	Consecutive patients undergoing SLNB (2007-2017)	Yes	T1= 11<br>T2 = 94<br>T3 = 70<br>T4 = 35

Stassen et al.	Supported in part by SkylineDx	Prospective Cohort Study	Multicenter (Netherlands)	pT1b-T4 clinically localized patients undergoing SLNB	Yes	T1= 74<br>T2 = 112<br>T3 = 54<br>T4 = 20
Yousaf et al.	NCI, Authorship by SkylineDx employees	Retrospective Cohort Study	Two academic centers (USA)	pT1-T4 clinically localized patients who underwent SLNB and retrospectively had CP-GEP testing		T1= 74<br>T2 = 79<br>T3 = 40<br>T4 = 15
**<u>31-GEP</u>**						
Vetto et al.	Castle Biosciences	Prospective/Retrospective Cohort Study	Multicenter (unspecified)	Combined cohorts of prospectively collected patients and those undergoing GEP at participating centers	No	T1/T2 = 1065
**<u>i31-GEP</u>**						
Whitman et al.	Castle Biosciences	Retrospective Cohort Study	Multicenter (USA and international)	T1-T4 undergoing GEP testing with either clinically or pathology determined SLN status		T1= 705<br>T2 = 534<br>T3 = 303<br>T4 = 132

Literature Search Protocol

A comprehensive literature search is an essential element of evidence analysis when developing clinical care recommendations. The quality of evidence reviewed directly impacts the quality of recommendations developed. A literature search protocol ensures consistent and comprehensive literature searches and evaluation across projects.

When a literature search is required, SSO staff will work with project members and a contracted medical librarian to run a comprehensive search using the following actions:

1. Define search parameters. Using a standardized form, search parameters are defined including:
    - Key search terms and connectors, with or without MESH terms
    - Time frames for search
    - English and/or other language
    - Geographic limits
    - Age range
    - Studies to be included (human, animal, and/or cadaver)
    - Publication/study types to be included
    - Databases to be searched.
        - Databases to be searched should minimally include MEDLINE/PubMed, EMBASE, and Cochrane Library. Additional databases may be added at the discretion of the requesting party.
    - Whether to eliminate duplicates between searches
    - Order of results
    - Whether searches should be separated by term or as one large package

2. Search results. Search results with abstracts will be assembled by the medical librarian. Results are sent to SSO staff, who will share it with the appropriate project members for review. Staff will retain search results for future use and documentation.

3. Review process

- SSO staff shares results with appropriate project members to assess relevance of articles and to identify those articles appropriate for full-article review and for which to run a "related articles" search.
- Staff coordinates additional level of searching on "related articles" with medical librarian. Results are reviewed by appropriate project members for relevance and identification of those for full-article review, and identification of a second search for additional "related articles" to be reviewed.
- Staff coordinates additional level of searching on "related articles" with medical librarian, if needed for further member review.

4. Article retrieval. Full-text articles will be retrieved for review based on the final list of selected abstracts.

5. Article review. Project members reviewing full-text articles should also review references at the end of each article to identify any articles missed in the search.

    - At least two project members should review each article.

Literature Search

Literature Search January 1, 2012-September 1, 2022

Search Strategies

Ovid MEDLINE

Interface: Ovid MEDLINE
Date searched: September 1, 2022

SEARCH	QUERY	RESULTS
#1	(English.lg. AND (exp Gene-Expression-Profiling/ OR Transcriptome/ OR (((gene? OR genet* OR geno*) ADJ4 (profil* OR expression)) OR transcriptom* OR DecisionDx).ti,ab.) AND (exp Melanoma/ OR Melanoma-Cutaneous-Malignant.rx. OR melano*.ti,ab.) AND (Sentinel-Lymph-Node-Biopsy/ OR (sentin#l OR SLN OR SLNB).ti,ab.)) NOT ((exp Animals/ NOT Humans/) OR exp Cadaver/ OR cadaver*.ti,ab. OR in-vitro.ti. OR (animal* OR dog OR dogs OR sheepdog OR canine OR cats OR feline OR horse* OR equine OR donkey* OR mouse OR mice OR murin?e OR woodmouse OR rat OR rats OR cottonrat* OR rodent* OR hamster* OR squirrel* OR chipmunk* OR otter* OR weasel* OR badger* OR beaver* OR llama* OR alpaca* OR rabbit* OR hare OR hares OR sheep OR ovine OR lamb* OR goat* OR	98<br><br>*Please note that the MeSH term Gene Expression Profiling has been superseded by Transcriptome as the preferred indexed term

	porcine OR swine* OR pig OR pigs OR piglet* OR boar OR boars OR hog OR hogs OR cow OR cows OR cattle* OR bull OR bulls OR bovine OR bison* OR buffalo* OR monkey* OR ape OR apes OR baboon* OR gibbon* OR bonobo* OR gorilla* OR lemur* OR chimp* OR orangutan* OR macaque* OR marmoset* OR primate* OR bear OR bears OR avian OR bird* OR hen OR hens OR chicken* OR duck? OR goose OR geese OR poultr* OR fowl? OR turkey* OR deer OR doe OR reindeer OR dolphin OR (fish* NOT fisher*) OR pisces OR trout* OR zebrafish* OR catfish* OR goldfish* OR seahorse* OR shark* OR salmon* OR whitefish* OR reptil* OR snake* OR lizard* OR alligator* OR crocodile* OR turtle* OR amphibian* OR frog* OR toad* OR eel? OR salamander* OR veterinar*).ti.)	beginning in 2012. As such, it has been included in this search.
#2	limit 1 to yr=2012-Current	76 – Final Result (75 after duplicates were removed)
	(exp Gene-Expression/ OR Genetic-Testing/ OR Genetic-Profile/ OR exp Oligonucleotide-Array-Sequence-Analysis/ OR ((gene? OR genet* OR geno* OR transcript*) ADJ4 (profil* OR expression OR express* OR analys* OR signature*)).ti,ab. OR (Decision-Dx OR Castle-Biosciences OR NeraCare OR Melagenix OR 31-gene OR thirty-one-gene OR nine-gene OR 9-gene OR eight-gene OR 8-gene OR GEP OR SkylineDx OR Merlin).ti,ab. OR (mRNA ADJ3 (differential OR display*)).ti,ab.) AND Melanocytes/ AND Sentinel-Lymph-Node/ OR (lymph-node?).ti,ab. AND (predict*.ti,ab. OR Predictive-Value-of-Tests/ OR prognos*.ti,ab. OR exp Prognosis/ OR (validation OR validate).ti,ab. OR Validation-Studies-as-Topic/ OR Validation-Studies.pt.)	These are tested terms that went unused because they either did not add additional articles to the search, provided articles that were irrelevant to the topic, or because they did not meaningfully focus the search as much as they were likely intended to (i.e., prognosis/predictive value/validation studies only reduced the search by about 20 articles). Those in red were removed from a query that ended up being used in the final search.

Query notes

.lg. = language
exp [Term]/ = MeSH Term, exploded (includes all references indexed to associated narrower MeSH terms as well)
[Term]/ = MeSH Term, not exploded
# = wildcard, 1 character
? = wildcard, 0 or 1 character
* = truncation, unlimited number of characters
ADJx = adjacency search; finds a term within x number of words from another term
.ti,ab. = title/abstract
.rx. = rare disease supplementary concept word
yr = publication year

Embase

Interface: Elsevier (http://www.embase.com)
Date searched: September 1, 2022

SEARCH	QUERY	RESULTS

#1	((gene-expression-profiling/exp OR transcriptome/de OR genetic-profile/de OR (((gene$ OR genet* OR geno*) NEAR/4 (profil* OR expression)) OR transcriptom* OR DecisionDx):ti,ab) AND (melanoma/exp OR cutaneous-melanoma/exp OR melano*:ti,ab) AND (sentinel-lymph-node-biopsy/de OR sentinel-lymph-node/de OR (sentin?l OR SLN OR SLNB):ti,ab) AND [english]/lim AND [2012-3000]/py) NOT (cadaver/de OR in-vitro-study/exp OR cadaver*:ti,ab OR in-vitro:ti OR animal-experiment/exp OR (animal* OR dog OR dogs OR sheepdog OR canine OR cats OR feline OR horse* OR equine OR donkey* OR mouse OR mice OR murin$e OR woodmouse OR rat OR rats OR cottonrat* OR rodent* OR hamster* OR squirrel* OR chipmunk* OR otter* OR weasel* OR badger* OR beaver* OR llama* OR alpaca* OR rabbit* OR hare OR hares OR sheep OR ovine OR lamb* OR goat* OR porcine OR swine* OR pig OR pigs OR piglet* OR boar OR boars OR hog OR hogs OR cow OR cows OR cattle* OR bull OR bulls OR bovine OR bison* OR buffalo* OR monkey* OR ape OR apes OR baboon* OR gibbon* OR bonobo* OR gorilla* OR lemur* OR chimp* OR orangutan* OR macaque* OR marmoset* OR primate* OR bear OR bears OR avian OR bird* OR hen OR hens OR chicken* OR duck$ OR goose OR geese OR poultr* OR fowl$ OR turkey* OR deer OR doe OR reindeer OR dolphin OR (fish* NOT fisher*) OR pisces OR trout* OR zebrafish* OR catfish* OR goldfish* OR seahorse* OR shark* OR salmon* OR whitefish* OR reptil* OR snake* OR lizard* OR alligator* OR crocodile* OR turtle* OR amphibian* OR frog* OR toad* OR eel$ OR salamander* OR veterinar*):ti)	128 – Final Result (89 after duplicates were removed)
	(gene-expression/exp OR genetic-screening/exp OR transcriptome-sequencing/exp OR DNA-microarray/exp OR ((gene$ OR genet* OR geno* OR transcript*) NEAR/4 (profil* OR expression OR express* OR analys* OR signature*)):ti,ab OR (Decision-Dx OR Castle-Biosciences OR NeraCare OR Melagenix OR 31-gene OR thirty-one-gene OR nine-gene OR 9-gene OR eight-gene OR 8-gene OR GEP OR SkylineDx OR Merlin):ti,ab OR (mRNA NEAR/3 (differential OR display*)):ti,ab) AND melanocyte/de AND (lymph-node$):ti,ab AND (predict*:ti,ab OR predictive-value/de OR prognos*:ti,ab OR prognosis/exp OR (validation OR validate):ti,ab OR validation-study/de)	These are tested terms that went unused because they either did not add additional articles to the search, provided articles that were irrelevant to the topic, or because they did not meaningfully focus the search as much as they were likely intended to (i.e., prognosis/predictive value/validation studies only reduced the search by about 20 articles). Those in red were removed from a query that ended up being used in the final search.

Query notes

/exp = Emtree term, exploded
/de = Emtree term, not exploded
$ = wildcard, 0 or 1 character
? = wildcard, 1 character
* = truncation, unlimited number of characters

NEAR/x = adjacency search; finds a term within x number of words from another term
:ti,ab = title/abstract
/lim = limits
/py = publication year
:it = publication type

The Cochrane Library

Includes Cochrane Database of Systematic Reviews and Cochrane Central Register for Controlled Trials
Interface: Wiley (http://onlinelibrary.wiley.com/cochranelibrary/search)
Date searched: September 1, 2022

SEARCH	QUERY	RESULTS
#1	(([mh "Gene-Expression-Profiling"] OR [mh Transcriptome] OR (((gene? OR genet* OR geno*) NEAR/4 (profil* OR expression)) OR transcriptom* OR DecisionDx):ti,ab) AND ([mh Melanoma] OR [mh "Melanoma Cutaneous Malignant"] OR melano*:ti,ab) AND ([mh "Sentinel Lymph Node Biopsy"] OR (sentin?l OR SLN OR SLNB):ti,ab)) NOT (cadaver*:ti,ab OR "in vitro":ti OR (animal* OR dog OR dogs OR sheepdog OR canine OR cats OR feline OR horse* OR equine OR donkey* OR mouse OR mice OR murin?e OR woodmouse OR rat OR rats OR cottonrat* OR rodent* OR hamster* OR squirrel* OR chipmunk* OR otter* OR weasel* OR badger* OR beaver* OR llama* OR alpaca* OR rabbit* OR hare OR hares OR sheep OR ovine OR lamb* OR goat* OR porcine OR swine* OR pig OR pigs OR piglet* OR boar OR boars OR hog OR hogs OR cow OR cows OR cattle* OR bull OR bulls OR bovine OR bison* OR buffalo* OR monkey* OR ape OR apes OR baboon* OR gibbon* OR bonobo* OR gorilla* OR lemur* OR chimp* OR orangutan* OR macaque* OR marmoset* OR primate* OR bear OR bears OR avian OR bird* OR hen OR hens OR chicken* OR duck? OR goose OR geese OR poultr* OR fowl? OR turkey* OR deer OR doe OR reindeer OR dolphin OR (fish* NOT fisher*) OR pisces OR trout* OR zebrafish* OR catfish* OR goldfish* OR seahorse* OR shark* OR salmon* OR whitefish* OR reptil* OR snake* OR lizard* OR alligator* OR crocodile* OR turtle* OR amphibian* OR frog* OR toad* OR eel? OR salamander* OR veterinar*):ti) *with Publication Year from 2012 to 2022, in Trials*	2 – Final Result (1 after duplicates were removed)

Query notes

[mh "[Term]"] = MeSH Term, exploded (includes all references indexed to associated narrower MeSH terms as well)
[mh ^"[Term]"] = MeSH Term, not exploded
? = wildcard, 1 character
* = truncation, unlimited number of characters
NEAR/x = adjacency search; finds a term within x number of words from another term
:ti,ab = title/abstract

Literature Search Update: September 2, 2022-August 7, 2023

Search Strategies

Ovid MEDLINE

Interface: Ovid MEDLINE

Date searched: August 7, 2023

SEARCH QUERY RESULTS

#1 (English.lg. AND (exp Gene-Expression-Profiling/ OR Transcriptome/ OR (((gene? OR genet* OR geno*) ADJ4 (profil* OR expression)) OR transcriptom* OR DecisionDx).ti,ab.) AND (exp Melanoma/ OR Melanoma-Cutaneous-Malignant.rx. OR melano*.ti,ab.) AND (Sentinel-Lymph-Node-Biopsy/ OR (sentin#l OR SLN OR SLNB).ti,ab.)) NOT ((exp Animals/ NOT Humans/) OR exp Cadaver/ OR cadaver*.ti,ab. OR in-vitro.ti. OR (animal* OR dog OR dogs OR sheepdog OR canine OR cats OR feline OR horse* OR equine OR donkey* OR mouse OR mice OR murin?e OR woodmouse OR rat OR rats OR cottonrat* OR rodent* OR hamster* OR squirrel* OR chipmunk* OR otter* OR weasel* OR badger* OR beaver* OR llama* OR alpaca* OR rabbit* OR hare OR hares OR sheep OR ovine OR lamb* OR goat* OR porcine OR swine* OR pig OR pigs OR piglet* OR boar OR boars OR hog OR hogs OR cow OR cows OR cattle* OR bull OR bulls OR bovine OR bison* OR buffalo* OR monkey* OR ape OR apes OR baboon* OR gibbon* OR bonobo* OR gorilla* OR lemur* OR chimp* OR orangutan* OR macaque* OR marmoset* OR primate* OR bear OR bears OR avian OR bird* OR hen OR hens OR chicken* OR duck? OR goose OR geese OR poultr* OR fowl? OR turkey* OR deer OR doe OR reindeer OR dolphin OR (fish* NOT fisher*) OR pisces OR trout* OR zebrafish* OR catfish* OR goldfish* OR seahorse* OR shark* OR salmon* OR whitefish* OR reptil* OR snake* OR lizard* OR alligator* OR crocodile* OR turtle* OR amphibian* OR frog* OR toad* OR eel? OR salamander* OR veterinar*).ti.) 108

#2 limit 1 to yr=2022-Current 23 – Final Result (11 after duplicates removed)

Query notes

.lg. = language

exp [Term]/ = MeSH Term, exploded (includes all references indexed to associated narrower MeSH terms as well)

[Term]/ = MeSH Term, not exploded

# = wildcard, 1 character

? = wildcard, 0 or 1 character

* = truncation, unlimited number of characters

ADJx = adjacency search; finds a term within x number of words from another term

.ti,ab. = title/abstract

.rx. = rare disease supplementary concept word

yr = publication year

Embase Interface: Elsevier (http://www.embase.com)

Date searched: August 7, 2023

SEARCH QUERY RESULTS

#1 ((gene-expression-profiling/exp OR transcriptome/de OR genetic-profile/de OR (((gene$ OR genet* OR geno*) NEAR/4 (profil* OR expression)) OR transcriptom* OR DecisionDx):ti,ab) AND (melanoma/exp OR cutaneous-melanoma/exp OR melano*:ti,ab) AND (sentinel-lymph-node-biopsy/de OR sentinel-lymph-node/de OR (sentin?l OR SLN OR SLNB):ti,ab) AND [english]/lim AND [2022-3000]/py) NOT (cadaver/de OR in-vitro-study/exp OR cadaver*:ti,ab OR in-vitro:ti OR animal-experiment/exp OR (animal* OR dog OR dogs OR sheepdog OR canine OR cats OR feline OR horse* OR equine OR donkey* OR mouse OR mice OR murin$e OR woodmouse OR rat OR rats OR cottonrat* OR rodent* OR hamster* OR squirrel* OR chipmunk* OR otter* OR weasel* OR badger* OR beaver* OR llama* OR alpaca* OR rabbit* OR hare OR hares OR sheep OR ovine OR lamb* OR goat* OR porcine OR swine* OR pig OR pigs OR piglet* OR boar OR boars OR hog OR hogs OR cow OR cows OR cattle* OR bull OR bulls OR bovine OR bison* OR buffalo* OR monkey* OR ape OR apes OR baboon* OR gibbon* OR bonobo* OR gorilla* OR lemur* OR chimp* OR orangutan* OR macaque* OR marmoset* OR primate* OR bear OR bears OR avian OR bird* OR hen OR hens OR chicken* OR duck$ OR goose OR geese OR poultr* OR fowl$ OR turkey* OR deer OR doe OR reindeer OR dolphin OR (fish* NOT fisher*) OR pisces OR trout* OR zebrafish* OR catfish* OR goldfish* OR seahorse* OR shark* OR salmon* OR whitefish* OR reptil* OR snake* OR lizard* OR alligator* OR crocodile* OR turtle* OR amphibian* OR frog* OR toad* OR eel$ OR salamander* OR veterinar*):ti) 33 – Final Result (11 after duplicates removed)

Query notes

/exp = Emtree term, exploded

/de = Emtree term, not exploded

$ = wildcard, 0 or 1 character

? = wildcard, 1 character

* = truncation, unlimited number of characters

NEAR/x = adjacency search; finds a term within x number of words from another term

:ti,ab = title/abstract

/lim = limits

/py = publication year

:it = publication type

The Cochrane Library

Includes Cochrane Database of Systematic Reviews and Cochrane Central Register for Controlled Trials
Interface: Wiley (http://onlinelibrary.wiley.com/cochranelibrary/search)

Date searched: August 7, 2023

SEARCH QUERY RESULTS

#1 (([mh "Gene-Expression-Profiling"] OR [mh Transcriptome] OR (((gene? OR genet* OR geno*) NEAR/4 (profil* OR expression)) OR transcriptom* OR DecisionDx):ti,ab) AND ([mh Melanoma] OR [mh "Melanoma Cutaneous Malignant"] OR melano*:ti,ab) AND ([mh "Sentinel Lymph Node Biopsy"] OR (sentin?l OR SLN OR SLNB):ti,ab)) NOT (cadaver*:ti,ab OR "in vitro":ti OR (animal* OR dog OR dogs OR sheepdog OR canine OR cats OR feline OR horse* OR equine OR donkey* OR mouse OR mice OR murin?e OR woodmouse OR rat OR rats OR cottonrat* OR rodent* OR hamster* OR squirrel* OR chipmunk* OR otter* OR weasel* OR badger* OR beaver* OR llama* OR alpaca* OR rabbit* OR hare OR hares OR sheep OR ovine OR lamb* OR goat* OR porcine OR swine* OR pig OR pigs OR piglet* OR boar OR boars OR hog OR hogs OR cow OR cows OR cattle* OR bull OR bulls OR bovine OR bison* OR buffalo* OR monkey* OR ape OR apes OR baboon* OR gibbon* OR bonobo* OR gorilla* OR lemur* OR chimp* OR orangutan* OR macaque* OR marmoset* OR primate* OR bear OR bears OR avian OR bird* OR hen OR hens OR chicken* OR duck? OR goose OR geese OR poultr* OR fowl? OR turkey* OR deer OR doe OR reindeer OR dolphin OR (fish* NOT fisher*) OR pisces OR trout* OR zebrafish* OR catfish* OR goldfish* OR seahorse* OR shark* OR salmon* OR whitefish* OR reptil* OR snake* OR lizard* OR alligator* OR crocodile* OR turtle* OR amphibian* OR frog* OR toad* OR eel? OR salamander* OR veterinar*):ti) with Publication Year from 2022 to 2023, in Trials 0 – Final Result

Query notes

[mh “[Term]”] = MeSH Term, exploded (includes all references indexed to associated narrower MeSH terms as well)

[mh ^“[Term]”] = MeSH Term, not exploded

? = wildcard, 1 character

* = truncation, unlimited number of characters

NEAR/x = adjacency search; finds a term within x number of words from another term

:ti,ab = title/abstract

Literature Flow Chart

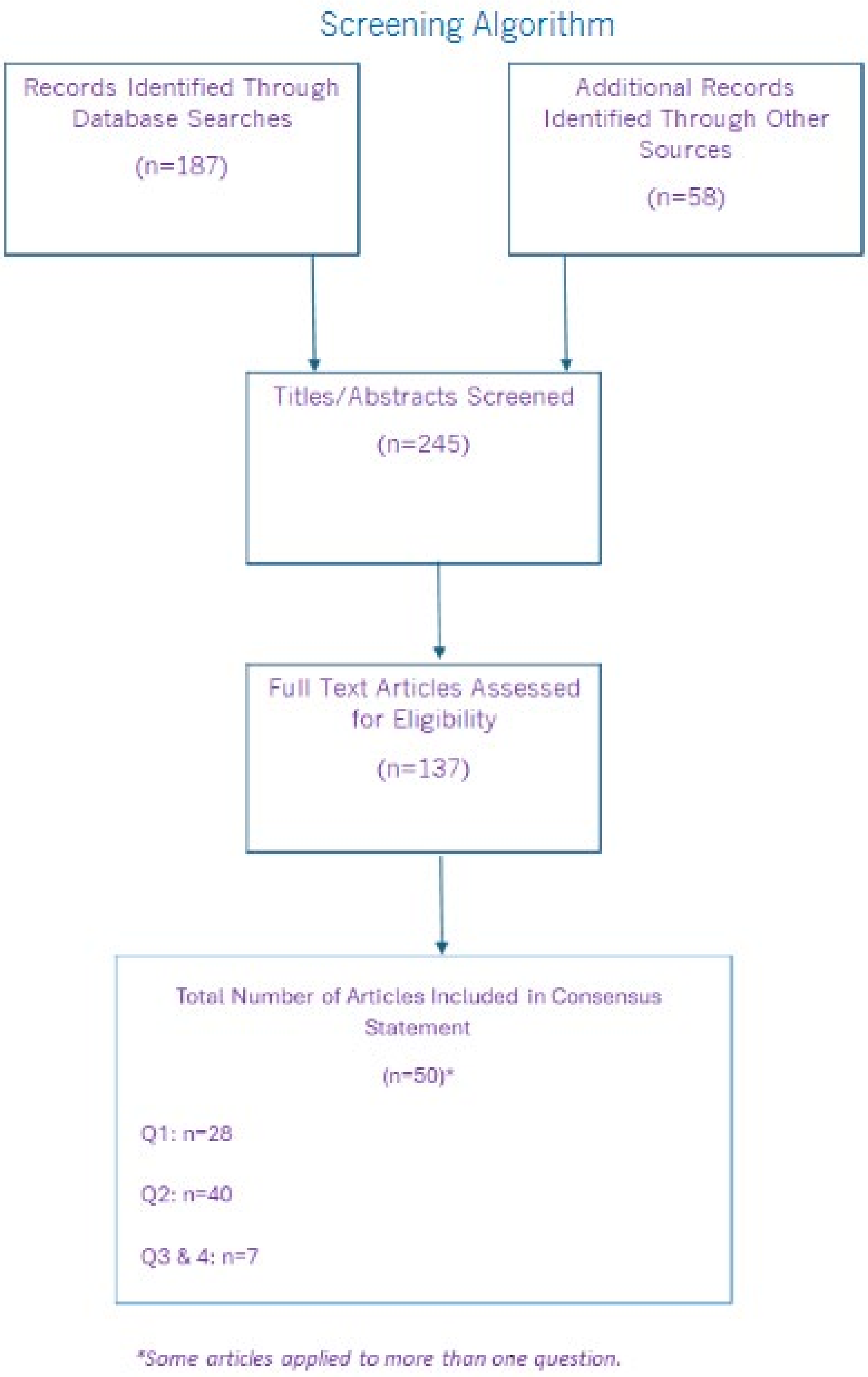

Levels of Evidence

Upon completion of a literature search and identification of appropriate evidence from that search, each evidentiary article must be reviewed and assigned levels of evidence. SSO has adopted the levels of evidence from the *American Association of Clinical Endocrinologists and American College of Endocrinology Protocol for Standardized Production of Clinical Practice Guidelines, Algorithms, and Checklists-2017 Update.*[1] SSO uses only Tables 5 and 6 from the ACCE/ACE scheme:

- Table 5-Revised Logical Ranking of Scientific Methodologies (Step I: Evidence Rating) to identify levels of evidence; and
- Table 6-Revised Evaluation of Studies (Step II: Scientific Analysis and Subjective Factors) for consideration when evaluating studies.

Individual reviewers complete an evidentiary table in its entirety for each article reviewed. Levels of evidence should be assigned for each article using the system described in Table 5 and taking into consideration the information in Table 6. Ratings will be used to summarize the literature for the topic.

	Summary of Evidence Table					
**Level of Evidence*^**<br><br>**Choose one:**	**Study Citation**<br>Author(s), Year, Title, Journal, Volume, Page #s	**Funding Source**	**Study Methodology**	**Objective & Methods**	**Results**	**Conclusions**
**Strong**<br>☐EL 1; RCT<br>☐EL 1; MRCT<br><br>**Intermediate**<br>☐EL 2; MNRCT<br>☐EL 2; NMA<br>☐EL 2; NRCT<br>☐EL 2; PCS<br>☐EL 2; RCCS<br>☐EL 2; NCCS<br>☐EL 2; CSS<br>☐EL 2; ES<br>☐EL 2; OLES<br>☐EL 2; PHAS<br><br>**Weak**<br>☐EL 3; DS<br>☐EL 3; ECON<br>☐EL 3; CCS<br>☐EL 3; SCR<br>☐EL 3; PRECLIN<br>☐EL 3; BR<br><br>**No Evidence**<br>☐EL 4; NE<br>☐EL 4: O	Citation:<br><br>Country:	Source:	Methodology:	Stated Objective:<br><br>☐ Prospective<br>☐ Retrospective<br><br>Study Population and Setting:<br><br>N:<br><br>Intervention:<br><br>Outcome Measures:<br><br>Follow-Up:<br><br>Notes:	Results:<br><br>Notes:	Describe conclusions relative to question:<br><br>Critiques of Methodology:

*See Table 5-Revised Logical Ranking of Scientific Methodologies (Step I: Evidence Rating) for explanation of levels of evidence.

^Consider the elements of Table 6-Revised Evaluation of Studies (Step II: Scientific Analysis and Subjective Factors) when evaluating studies.

Mechanick JI, Pessah-Pllack R, Camacho P, Correa R, Figaro MK, Garber JR, et al. American Association of Clinical Endocrinologists and American Collee of Endocrinology Protocol for Standardized Production of Clinical Practice Guidelines, Algorithms, and Checklists-2017 Update. Endocr Pract. 2017; 8:1006-1021.

Table 5
Revised Logical Ranking of Scientific Methodologies (Step I: Evidence Rating)[a]

Numerical Descriptor[b]	Semantic Descriptor	Methodology Descriptor
STRONG EVIDENCE		
1 (1)	RCT	Randomized controlled trial[c]
1 (1)	MRCT	Meta-analysis of only randomized controlled trials
INTERMEDIATE EVIDENCE		
2 (2)	MNRCT	Meta-analysis including nonrandomized prospective or case-controlled trials

2 (new)	NMA	Network meta-analysis[2,3]
2 (2)	NRCT	Nonrandomized controlled trial (or unconfirmed randomization)
2 (2)	PCS	Prospective cohort study (does not include open-label extension study)
2 (2)	RCCS	Retrospective case-control study
2 (new)	NCCS	Nested case-control study
2 (3;reassigned)	CSS	Cross-sectional study
2 (3;reassigned)	ES	Epidemiological study (hypothesis driven; includes survey, registry, data-mining, with or without retrospective uni-multivariate analyses or propensity matching)
2 (new)	OLES	Open-label extension study[4]
2 (new)	PHAS	Post hoc analysis study[5]
WEAK EVIDENCE		
3 (new)	DS	Discovery science (explorative/inductive; includes -omics, "big data," network analysis, systems biology, Bayesian inference, modeling[6]
3 (new)	ECON	Economic study (includes Markov models, pharmaco-economics)[7-11]
3 (3)	CCS	Consecutive case series (N>1)
3 (3)	SCR	Single case report (N=1)
3 (new)	PRECLIN	Preclinical study (e.g., feasibility, safety)
3 (new)	BR	Basic research (mut be high impact and relevant)
NO EVIDENCE		
4 (4)	NE	No evidence (theory, opinion, consensus, review, position, policy, guideline)
4 (new)	O	Other (e.g., low impact/relevant basic research; any highly flawed study)

Abbreviations: EBM=evidence-based methodology; EL=evidence level.
[a]Based on principal that interventions, scientific control, generalizability, methodological flaws, and evidentiary details determine strength,[12] consistent with other EBM systems (reviewed in Table 2 in reference[13]). Numerical and semantic descriptors of Els in on-line supplementary material.
[b]The original numerical description from G4GAC 2004, 2010, and 2014 are provided in parentheses.
[c]The superiority of RCT over all other studies, and in particular MRCT, is discussed in reference.[14] MRCT are inferior to RCT due to the bias introduced by being a retrospective analysis.[15]

Table 6
Revised Evaluation of Studies (Step II: Scientific Analysis and Subjective Factors)[a]

Study design[b]	Data analysis[b]	Interpretation of results
Allocation concealment (randomization)	Intent-to-treat	Generalizability
Blinding[c]	Modeling (e.g., Markov)	Incompleteness
Comparator group	Network analysis	Logical
Endpoints (real clinical vs. surrogate)	Statistics	Overstated
Hypothesis	Appropriate follow-up[16]	Validity
Power analysis (too small sample size)	Appropriate trial termination[17]	
Premise		
Type I error (e.g., adjusted for PHAS)		

Abbreviation: PHAS=post hoc analysis study.
[a]These subjective factors pertain to an individual citation. Subjective factors are provided in on-line supplementary material.
[b]Are these elements appropriate for the givent study?
[c]Including patients, clinicians, data collectors, adjudicators of outcome, and data analysis.

1.Mechanick JI, Pessah-Pllack R, Camacho P, Correa R, Figaro MK, Garber JR, et al. American Association of Clinical Endocrinologists and American College of Endocrinology Protocol for Standardized Production of Clinical Practice Guidelines, Algorithms, and Checklists-2017 Update. Endocr Pract. 2017; 8:1006-1021.
2. Li T, Puhan MA, Vedula SS, et al. Network meta-analysis-highly attractive but more methodological research is needed. BMC Med. 2011l70:52-60.
3. Kanters S, Ford N, Druyts E, Thorlund K, Mills EJ, Bansback N. Use of network meta-analysis in clinical guidelines. Bull World Health Organ. 2016;94:782-784.
4. Ray RO, Williams KM. Open-label extension studies: do they provide meaningful information on the safety of new drugs? Drug Saf. 2007;30:93-105.
5. Elliott HL. Post-hoc analysis: use and dangers in perspective. J Hypertens Suppl. 1996;14:S21-S24.
6. Davidson EH, Genomics, "Discovery Science," systems biology, and causal explanation: what really works? Perspect Biol Med. 2015;58:165-181.
7. Brown GC, Brown MM. Value-based medicine and pharmacoeconomics. Dev Ophthalmol 2016;55:381-390.
8. Aggarwal S, Topaloglu H, Kumar S. Systematic review on use of economic evidence in clinical guidelines. Value Health. 2015;18;A516.
9.Subedi P, Kamal-Bahl S. Economic considerations in clinical guidelines-Are patient perspectives considered? Value Health 2015;18:A565-A566.
10. Hlatky MA. Considering cost-effectiveness in cardiology clinical guidelines: progress and prospects. Value Health. 2016;19:516-519.
11. Drummond M. Clinical Guidelines: A NICE way to introduce cost-effectiveness considerations? Value Health. 2016;19:525-530.
12. Barton S. Which clinical studies provide the best evidence? BMJ 2000;321:255-256.
13. Mechanick JI, Camacho PM, Cobin RH, et al. American Association of Clinical Endocrinologists Protocol for Standardized Production of Clinical Practice Guidelines—2010 Update. Endocr Pract. 2010;16:798-804.
14. Seifres CM, Iams JD, Klebanoff M, et al. Metaanalysis vs large clinical trials: which should guide our management? Am J Obstet Synecol. 2009;200:484. e1-5.

15. Pal S. Randomized trials vs. meta-analyses: which is the better bet? ASCO Post 2014. Available at: http://www.ascopost.com/issues/june-10-2014/randomized-trials-vs-meta-analyses-which-is-the-better-bet/. Accessed February8, 2017.
16. Ioannidis JP, Bassett R, Hughes MD, Volberding PA, Sacks HS, Lau J. Predictors and impact of patient lost to follow up in a long-term randomized trial of immediate versus deferred antiretroviral treatment. J Acquir Immune Defic Syndr Hum Retrovirol. 1997;16:22-30.
17. Montori VM, Devereaux PJ, Adhikari NK, et al. Randomized trial stopped early for benefit: a systematic review. JAMA. 2005;294:2203-2209.

SSO Modified Delphi Process

Select Consensus Voting Panel	The Consensus Voting Panel will consist of the Expert Panel, plus a Consensus Voting Group. The full panel should consist of approximately 20-25 members. Members should be representative of relevant specialties, practice settings (ie, both community and academic settings), geographic representation, diverse, and represent a variety of viewpoints within the field.<br><br>All consensus panel members will complete a conflict-of-interest disclosure which will be evaluated and managed in accordance with the SSO disclosure policy. Panel members and their disclosures will be included in the final manuscript.
Develop Consensus Recommendations	Consensus statements are drafted by Expert Panel groups assigned to each clinical question. Each group drafts only the recommendations related to their question to minimize any group bias in recommendation development and voting.<br><br>Recommendations should be informed by the evidence review for the question and include considerations of population, intervention, comparator, and outcome (PICO). Recommendations for each question will be supported by clinical rationale and a referenced evidentiary statement in the final text.<br><br>The Expert Panel may review and edit the evidentiary statements or tables for accuracy. Recommendations may be viewed but not edited until consensus voting, unless corrections are required to be consistent with the presented evidence.
Questionnaire Development	Questionnaires will be in the form of electronic surveys shared via email link.
Consensus Voting Rounds	Participants will vote anonymously in each round on each recommendation and its accompanying supporting evidence (either in the form of evidentiary statements or tables) using a five-point Likert scale. Participants are expected to abstain if they have a conflict with an affected company or are unable to answer. Comments may be provided about any recommendation or evidence. Comments and/or reasons are mandatory for decision, if disagreed or abstained.
Round 1	Submit questionnaire to Consensus Voting Panel for anonymous ratings and written feedback.

	Strongly Agree	Agree	Neither Agree nor Disagree	Disagree	Strongly Disagree	Abstain/ Unable to Answer

Recommendation						
Supporting Evidence						
Recommendation Comments						
Evidence Comments						

Round 1 Review

Staff will compile ratings and comments for each round. Only those recommendations without consensus will move to the next round.

Responses are kept anonymous, with only the staff liaison having access to individual results. The moderator group and others only have access only to de-identified aggregate survey responses.

	Strongly Agree	Agree	Neither Agree nor Disagree	Disagree	Strongly Disagree	Abstain/ Unable to Answer	Percent Agreement (responses "Strongly Agree" or "Agree")
Recommendation							
Supporting Evidence							

Each recommendation receiving a minimum of 80% agreement within the group responses (responses "Strongly Agree" and "Agree") are considered to have reached consensus. Abstentions are excluded from this calculation. Non-content wording changes that are minor can be determined through a basic majority vote.

Round 2

Responses not receiving 80% agreement will be reviewed by the moderators (chairs or chairs plus others selected by the chairs) and any necessary modifications may be made based on the feedback from the raters. If contextual changes to recommendations are made, only the changes are to be sent to the voting panel for additional rating.

These recommendations are sent out for Round Two in a new questionnaire that includes both new and previous iteration of the recommendations presented.

	Strongly Agree	Agree	Neither Agree Nor Disagree	Disagree	Strongly Agree	Abstain/ Unable to Answer	Percent Agreement (responses "Strongly Agree" or "Agree"
Previous Recommendation	Rating	Rating	Rating	Rating	Rating		Percentage
Updated Recommendation with changes highlighted							

	Staff compiles ratings and comments.
Voting Process	Voting process ends when consensus is reached or it is clear consensus cannot be reached after three rounds. If after a third-round consensus is not achieved, the SSO may choose not to pursue the recommendations.

Society of Surgical Oncology. SSO Guidelines for Internally Generated Statements 4.21.20.

Savic LC, Smith AF. Reviewer Recommendations: How to Conduct a Delphi Consensus Process. Anaesthesia. 2023; 78:247-250.

---

[1] BRAF Mutation and Cancer. Johns Hopkins Medicine-Health. Available at: https://hopkinsmedicine.org/health/conditions-and-diseases/braf-mutation-and-cancer. Accessed: 1/10/24.
[2] Pham DM, Guhan S. Tsao H. KIT and Melanoma: Biological Insights and Clinical Implications. *Yonsei Med J.* 202 Jul 1; 61(7): 562-571.
[3] Cancer Staging. National Cancer Institute: About Cancer. Available at: https://www.cancer.gov/about-cancer/diagnosis-staging/staging. Accessed 1/10/24.
[4] Munoz-Couselo E, Zamora-Adelantado E, Ortiz C, Soberino Garcia J, Perez-Garcia J. NRAs-Mutant Melanoma: Current Challenges and Future Prospect. *Onco Targets Ther.* 2017; 10:3941-3947.
