# Supplementary material for: Society of Surgical Oncology Consensus Statement: Assessing the Evidence for and Utility of Gene Expression Profiling of Primary Cutaneous Melanoma
Source: Ann Surg Oncol. 2024 Oct 29;32(3):1429–42. doi: 10.1245/s10434-024-16379-2 (PMC11811439; doi:10.1245/s10434-024-16379-2)
Supplement: Supplementary file 2 — Supplementary file2 (PDF 1497 kb) [file 10434_2024_16379_MOESM2_ESM.pdf]

SSO
SOCIETY OF SURGICAL ONCOLOGY
LEADING. TOGETHER.

Consensus Statement Evidentiary Tables :

Assessing the Evidence for and Utility of Gene Expression Profiling of Primary Cutaneous Melanoma*

Levels of evidence adopted from *American Association of Clinical Endocrinologists and American College of Endocrinology Protocol for Standardized Production of Clinical Practice Guidelines, Algorithms, and Checklists-2017 Update.*

Mechanick JI, Pessah-Pllack R, Camacho P, Correa R, Figaro MK, Garber JR, et al. American Association of Clinical Endocrinologists and American Collee of Endocrinology Protocol for Standardized Production of Clinical Practice Guidelines, Algorithms, and Checklists-2017 Update. Endocr Pract. 2017; 8:1006-1021.

Bookmarks to Tables for Each Question

Question 1

Question 2

Question 3 + Future Directions for Research

Question 1

In adult patients with AJCC pT1a-pT4b primary cutaneous melanoma, does GEP testing improve patient selection and decision-making for sentinel lymph node biopsy as compared to the use of conventional clinical and pathologic factors alone?

	Summary of Evidence Table<br>Question 1, 2					
Level of Evidence*^<br><br>Choose one:	Study Citation<br>Author(s), Year, Title, Journal, Volume, Page #s	Funding Source	Study Methodology	Objective & Methods	Results	Conclusions
Strong<br>☐EL 1; RCT<br>☐EL 1; MRCT<br><br>Intermediate<br>☐EL 2; MNRCT<br>☐EL 2; NMA<br>☐EL 2; NRCT<br>☐EL 2; PCS<br>☐EL 2; RCCS<br>☐EL 2; NCCS<br>☐EL 2; CSS<br>☐EL 2; ES<br>☐EL 2; OLES<br>☐EL 2; PHAS<br><br>Weak<br>☐EL 3; DS<br>☐EL 3; ECON<br>☒EL 3; CCS<br>☐EL 3; SCR<br>☐EL 3; PRECLIN<br>☐EL 3; BR<br><br>No Evidence<br>☐EL 4; NE<br>☐EL 4: O	Amaral T, Sinnberg T, Chatziioannou E, et al. Identification of Stage I/II Melanoma Patients at High Risk for Recurrence Using a Model Combining Clinicopathologic Factors with Gene Expression Profiling (CP_GEP). *Eur J Cancer.* 2023;182:155-162.<br><br>*Country:*<br>Germany, Netherlands	*Source:*<br>The study was partially funded by SkylineDx.<br><br>4 authors: SkylineDx employees<br><br>2 authors: institutional funding from SkylineDx in relation to the submitted work.<br><br>2 authors: Institutional financial support from Neracare<br><br>5 authors: stock/ownership interest in SkylineDx<br><br>1 author: leadership SkylineDx<br><br>1 author: consulting or advisory role	*Methodology*:<br>Blinded retrospective single-center study of stage I/II patients Single center retrospective review of a prospective database.	*Stated Objective:*<br>To validate CP-GEP for the identification of high-risk for disease recurrence stage I/II patients.<br><br>☐Prospective<br>☒Retrospective<br><br>*Study Population and Setting:*<br>Stage I/II, age >18, 2000-2017, with negative SLNB<br><br>*N:* 543 patients with SLNB+ 83 control without SLNB, of which 80 were analyzed<br><br>*Intervention*: CP-GEP with clinicopathologic features age and Breslow thickness with the expression of (ITGB3, PLAT, SERPINE2, GDF15, TGFBR1, LOXL4, CXCL8 and MLANA) corrected by (RLP0 and ACTB)<br><br>CP-GEP combined protocol to predict recurrence	*Results:*<br>W/ SLNB<br>CP-GEP stratified 424 patients (78% cohort) into RFS rates of 77.8% for high risk (195 patients) and 93% for low risk (229 patients). HR 3.53 (p, 0.001).<br><br>RFS stage I 90.7%, stage II 66.1%<br>DMFS stage I 96.0%, stage II 82.2%.<br>OS stage I 95.6%, stage II 79.0%<br><br>For stage I/II: CP-GEP classified 311 as high risk with HR 4.73 with p-value <0.001<br><br>Subgroup I/IIA: CP-GEP stratified low v high-risk with a HR of 3.53 (p <0.001).<br><br>RFS I/IIA for high-risk (195 patients) was 77.8% and 93.0% for low-risk.<br><br>W/O SLNB	*Describe conclusions relative to question:*<br><br>CP-GEP predicts high risk patients in stage I/II and could be used to replace SLNB and to guide adjuvant treatments.<br><br>CP-GEP could replace SLNB,<br><br>CP-GEP enhances traditional histologic classification of patients.<br><br>*Critiques of Methodology:*<br>Group stages I/II and don't separate to be able to discern clinicopathologic characteristics vs CP-GEP. The stage I/II vs I/IIA

				Outcome Measures: Kaplan-Meier curves to determine prognostic value.<br>Primary endpoint was RFS also DMFS and OS.<br><br>Recurrence after negative SLNB<br><br>*Follow-Up*: Median follow up of 83.63 months for patients with negative SLNB; 40.77 mo for patients without SLNB<br><br>*Notes*	Classified 11/80 high-risk which captured 6/7 recurrences.<br><br>The five-year RFS for stage I/II patients was 79.9% (95% CI: 76.0%e83.2%). CP-GEP identified 311 patients as high risk for disease recurrence with a HR of 4.73 with a p-value <0.001, capturing 83 out of 98 reported relapses. For subgroup stage I/IIA, CP-GEP was able to significantly stratify CPGEP low-risk and high-risk patients with a HR of 3.53 (p value <0.001) for five-year RFS.<br>For stage I/IIA, the five-year RFS rates for CP-GEP high-risk patients were 77.8% (95% CI: 70.9%e83.3%) and 93.0% (95% CI: 88.5%e95.8%) for CP-GEP low-risk patients.<br>"Compared to AJCC low-risk (stage I/IIA) patients with an RFS rate of 86.0% (95% CI: 82.0%e89.1%), CP-GEP was able to split 195 high-risk patients who had a worse five year RFS survival of 77.8%"<br><br>Notes:Wo SLNB high-risk curves are dramatically different, does this argue for doing a SLNB.	Initial curves do not separate by histologic stage. Group "Stage I/II" together.

					Without SLNB5/11 classified as high-risk without recurrence.<br><br>They group the analyses into “who would qualify for adjuvant therapy vs who wouldn’t” There is no subset analysis by the actual histologic stages.	

	Summary of Evidence Table<br>Questions 1, 2					
Level of Evidence*^<br><br>Choose one:	Study Citation<br>Author(s), Year, Title, Journal, Volume, Page #s	Funding Source	Study Methodology	Objective & Methods	Results	Conclusions
☐EL 1; RCT<br>☐EL 1; MRCT<br>☐EL 2; MNRCT<br>☐EL 2; NMA<br>☐EL 2; NRCT<br>☒EL 2; PCS<br>☐EL 2; RCCS<br>☐EL 2; NCCS<br>☐EL 2; CSS<br>☐EL 2; ES<br>☐EL 2; OLES<br>☐EL 2; PHAS<br><br>☐EL 3; DS<br>☐EL 3; ECON<br>☐EL 3; CCS<br>☐EL 3; SCR<br>☐EL 3; PRECLIN<br>☐EL 3; BR<br><br>☐EL 4; NE<br>☐EL 4: O<br><br>Strong / Intermediate / Weak / No Evidence	Arnot SP, Han G, Fortino J, Han D, Fowler G, Vetto JT. Utility of a 31-Gene Expression Profile for Predicting Outcomes in Patients with Primary Cutaneous Melanoma Referred for Sentinel Node Biopsy. *Am J Surg* 2021;221(6):1195-1199. (71)<br><br>*Country:* USA	*Source:*<br>Non-industry sponsored<br><br>1 author declared an honorarium from Castle Biosciences.	*Methodology:*<br>Retrospective cohort study (convenience study; not consecutive patients).<br><br>Prospective collection of GEP prior to operation then correlative work done.	*Stated Objective:*<br><br>Assess outcomes in Class I vs Class II<br><br>Correlate clinical finding to GEP.<br><br>☒Prospective sample collection<br>☒Retrospective study of a prospective database<br><br>*Study Population and Setting:*<br>Single institution<br>University setting<br><br>*N:* 383<br><br>*Intervention*:<br>31-GEP<br>GEP testing<br><br>*Outcome Measures:*<br>RFS, DMFS<br><br>*Follow-Up*: 32 mos (1-68)<br><br>*Notes:*	*Results:*<br>Class II had worse RFS, DMFS.<br><br>"Breslow thickness, T stage, and SNB positivity were significantly higher in Class 2 patients. Recurrence rates were higher for Class 2 vs Class 1 patients and highest in patients who were Class 2 and SNB positive. GEP class was predictive of RFS and DMFS and independently predicted relapse in AJCC "low risk" (stages IA-IIA) patients."<br><br>*Notes:* All included patients had 31-GEP and SLNB.	*Describe conclusions relative to question:*<br><br>31-GEP results added data for stage Ia and IIa patients.<br><br>*Critiques of Methodology:*<br>Selection bias; Appears to be a convenience sample and not all consecutive patients undergoing SLNB.<br><br>Correlative to RFS and DMFS.<br><br>Small series<br><br>Single institution<br><br>Retrospective analysis of a convenience sample.

Summary of Evidence Table<br>Question 1						
Level of Evidence*^<br><br>Choose one:	Study Citation<br>Author(s), Year, Title, Journal, Volume, Page #s	Funding Source	Study Methodology	Objective & Methods	Results	Conclusions
Strong<br>☐EL 1; RCT<br>☐EL 1; MRCT<br><br>Intermediate<br>☐EL 2; MNRCT<br>☐EL 2; NMA<br>☐EL 2; NRCT<br>☐EL 2; PCS<br>☒EL 2; RCCS<br>☐EL 2; NCCS<br>☐EL 2; CSS<br>☐EL 2; ES<br>☐EL 2; OLES<br>☐EL 2; PHAS<br><br>Weak<br>☐EL 3; DS<br>☐EL 3; ECON<br>☐EL 3; CCS<br>☐EL 3; SCR<br>☐EL 3; PRECLIN<br>☐EL 3; BR<br><br>No Evidence<br>☐EL 4; NE<br>☐EL 4: O	Bellomo D, Arias-Mejias SM, Ramana C, et al. Model Combining Tumor Molecular and Clinicopathologic Risk Factors Predicts Sentinel Lymph Node Metastasis in Primary Cutaneous Melanoma. *JCO Precis Oncol.* 2020;4:319-334. (53)<br><br>*Country:*<br><br>United States with international partners	*Source:*<br>National Cancer Institute (grant CA215105), National Center for Advancing Translational Sciences (grant UL1TR000135), Mayo Clinic Center for Individualized Medicine, Mayo Clinic Cancer Center, and the 5th District Eagles Cancer Telethon. Alexander Meves<br><br>4 authors: employees of SkylineDx<br><br>4 authors: stock and other ownership interests in SkylineDx<br><br>1 author: patents, royalties, other intellectual property-gene signatures predicting	*Methodology:*<br>Archival analysis of a prospective cohort.<br><br>Cohort retrospectively identified were analyzed by PCR.<br><br>Feature selection and parameter estimation were performed through a penalized maximum likelihood estimation algorithm through least absolute shrinkage and selection operator (LASSO).<br><br>Logistic regression models estimated the probability of SLN metastasis and was converted into binary results: Samples with a probability of metastasis greater than the cutoff were classified as positive, whereas samples with a probability	*Stated Objective:*<br>To identify CM clinicopathologic variables and a gene expression profile that associate with a low risk of SLN metastasis (an average risk of nodal metastasis < 5%).<br><br>☐Prospective<br>☒Retrospective<br><br>A consecutive case series and not a prospective cohort study after reviewing the analysis.<br><br>Study Population and Setting:<br>Breslow 1.0 mm-4.0mm,<br>OR depth 0.75-0.99 with ulceration, mitoses and or <40 years;<br>OR depth 0.5-0.74 mm and two of the following : ulceration, mitoses, age < 40.<br><br>Exclusion M1 within 90 days of diagnosis, insufficient primary tumor tissue<br><br>*N:* 754<br>(1/2 of the patients were previously reported by the same group)<br><br>*Intervention*: Combined clinical and GEP analysis of risk factors for SLN status.	*Results:*<br>The authors report a SLNB reduction rate in T1b tumors (they did stratify by T-stage, unlike other studies) of 80% however the accuracy of the test in this group was only 80% as well, impacting the actual utility of the statistic (since the test missed 20% of patients, presumably).<br><br>128/754 (17%) had +SLN<br><br>Overall discriminatory ability<br>of the CP model (AUC, 0.78; 95% CI, 0.74 to 0.82) and<br>GEP (AUC, 0.78; 95% CI, 0.73 to 0.82) was improved by combining CP factors and a GEP (AUC, 0.82; 95% CI,<br>0.78 to 0.86).<br><br>Combined CP-GEP model achieved an ap-<br>proximately 15% higher SLNB reduction rate compared<br>with the benchmark CP model at a negative predictive value<br>of 95%.	*Describe conclusions relative to question:*<br><br>There is concern with the statistical analysis which impacts the conclusion that adding GEP to CP improves in selecting patients for SLNB.<br><br>GEP added to standard CP predictors of SLN status most effectively in the T1b category. This appears to be confounded by the accuracy of the test and not considered in the discussion.<br><br>CP-GEP<br><br>The statement that the CP-GEP profile reduces the incidence of SLNB performed by 42% is reasonable, but there is no data

		metastasis of melanoma<br><br>1 author: research funding from SkylineDx	lower than the cutoff were classified as negative.<br><br>Model validated by repeated cross-validation or bootstrapping.<br><br>GEP defined from 11 genes that differentiated the patients with and without nodal metastasis detected by SLNB within 90 days of primary diagnosis: ADAM metallopeptidase domain 12 (ADAM12), interleukin 8 (CXCL8), growth differentiation factor 15 (GDF15), integrin-β3 (ITGB3), galectin 1 (LGALS1), lysyl oxidase like 4 (LOXL4), melanoma antigen recognized by T cells 1 (MLANA), tissue-type plasminogen activator (PLAT), protein kinase C-β (PRKCB), glia-derived nexin (SERPINE2), and transforming growth factor-β (TGF-β) receptor 1 (TGFBR1).	CP-GEP model<br>GEP profile plus standard histologic parameters, creative modeling.<br><br>(Clinicopathologic variables with eight-gene GEP)<br>*Outcome Measures:* SLN positivity within 90 days of diagnosis.<br><br>SLN metastasis within 90 days of melanoma diagnosis<br><br>*Follow-Up:* Appears to be 90 day follow up only (no subsequent follow up for false negative, in-basin recurrence<hr>*Notes*: Excluded high risk (T4 patients and performed SLNB in T1a patients that have “high risk features,” lower than many institutional thresholds	T1b SLNB reduction rate 80%<br>T2a SLNB reduction rate 48% with NNP 95%<br><br>Compare MSKCC nomogram which performed similarly to CP model but out-performed by the CP-GEP AUC<br><br>This is a complicated statistical analysis, but the modeling seems effective. Not sure that it proves to be statistically more powerful than clinicopathological criteria alone, in real-world use. A statistician would be helpful for this study.<hr>Notes: evaluated multiple CP factors to predict SLN+ and showed age and Breslow depth most predictive<br><br>Stratified by T stage (table 4).<hr>*Notes:*	on in-nodal basin recurrences therefore the accuracy of this test longitudinally is unclear.<br><br>*Critiques of Methodology:* Sophisticated statistical analysis.<br><br>AUC greatest for combined model CP-GEP but did not analyze if statistically significant improvement.<br><br>It is a statistical model. The discussion implies that the only benefit of SLNB is to determine adjuvant therapy, but there are other benefits.

			Logistic regression modeling was used to develop a novel model combining CP factors (i.e.: Breslow depth and patient age) and a GEP.<br><br>CP-GEP model was based on the expression of MLANA, a melanosome marker, and seven genes<br><br>Functionally linked to EMT and with specific roles in an-giogenesis/hypoxia and coagulation: GDF15,<br><br>CXCL8,<br><br>LOXL4, TGFBR1, ITGB3, PLAT, and SERPINE2<br><br>Retrospective analysis of prospectively collected specimens with follow up			

	Summary of Evidence Table<br>Question 1, 2, 3					
Level of Evidence*^<br><br>Choose one:	Study Citation<br>Author(s), Year, Title, Journal, Volume, Page #s	Funding Source	Study Methodology	Objective & Methods	Results	Conclusions
Strong<br>☐EL 1; RCT<br>☐EL 1; MRCT<br><br>Intermediate<br>☐EL 2; MNRCT<br>☐EL 2; NMA<br>☐EL 2; NRCT<br>☐EL 2; PCS<br>☐EL 2; RCCS<br>☐EL 2; NCCS<br>☐EL 2; CSS<br>☐EL 2; ES<br>☐EL 2; OLES<br>☐EL 2; PHAS<br><br>Weak<br>☐EL 3; DS<br>☐EL 3; ECON<br>☐EL 3; CCS<br>☐EL 3; SCR<br>☐EL 3; PRECLIN<br>☐EL 3; BR<br><br>No Evidence<br>☒EL 4; NE<br>☐EL 4: O	Berman B, Ceilley R Cockerell C. Appropriate Use Criteria for the Integration of Diagnostic and Prognostic Gene Expression Profile Assays into the Management of Cutaneous Malignant Melanoma: An Expert Panel Consensus-Based Modified Delphi Process Assessment. *SKIN The Journal of Cutaneous Medicine*, 2019; 3(5): 291–306.<br><br>*Country:* USA	*Source:*<br><br>The National Society for Cutaneous Medicine (a 501(c)3 non-profit entity) funded the consensus development program. The group has received unrestricted educational grants from related companies in volved with these technologies.<br><br>5 authors: Castle Bioscience consultant<br><br>2 authors: Castle Bioscience investigator<br><br>2 authors: Castle Bioscience honoraria<br><br>3 authors: Castle Bioscience	*Methodology:*<br><br>Expert panel consensus based Modified Delphi Process<br><br>3 GEP tests evaluated: 2-GEP Test, myPath from Myriad, Castle 31-GEP<br><br>Literature review	*Stated Objective:*<br><br>The objective of this expert panel was to develop a set of consensus-based AUC recommendations to guide the integration of GEP technology into the diagnosis and management of melanoma<br><br>☐Prospective<br>☐Retrospective<br><br>*Study Population and Setting*:<br>Literature review of 33 manuscripts.<br>Consensus panel of 9 physicians (dermatology, dermatopathology, surgery)	*Results:*<br>Several clinical scenarios evaluated and consensus-based recommendations made based on the taxonomy below.<br><br>One of 14 scenarios received an A-strength recommendation: Use of the 31-GEP test to aid in the management of patients who are SLNB negative<br><br>___<br>*Notes:*<br>Based on SORT Taxonomy, A = Consistent, good-quality patient-oriented evidence, B = Inconsistent or limited quality patient-oriented evidence, C = Consensus, disease-oriented evidence, usual practice, expert opinion, or case series	*Describe conclusions relative to question:*<br><br>Consensus-based framework for using GEP tests in clinical management.<br><br>*Critiques of Methodology:*<br><br>Limited number of physicians on consensus panel<br><br>Framework could be limited by biases of panel physicians<br><br>No new data presented; literature review of existing studies that may have individual weaknesses

		advisory board				

	Summary of Evidence Table<br>Question 1, 2, 3					
Level of Evidence*^<br><br>Choose one:	Study Citation<br>Author(s), Year, Title, Journal, Volume, Page #s	Funding Source	Study Methodology	Objective & Methods	Results	Conclusions
Strong<br>☐EL 1; RCT<br>☐EL 1; MRCT<br><br>Intermediate<br>☐EL 2; MNRCT<br>☐EL 2; NMA<br>☐EL 2; NRCT<br>☐EL 2; PCS<br>☐EL 2; RCCS<br>☐EL 2; NCCS<br>☐EL 2; CSS<br>☐EL 2; ES<br>☐EL 2; OLES<br>☐EL 2; PHAS<br><br>Weak<br>☒EL 3; DS<br>☐EL 3; ECON<br>☐EL 3; CCS<br>☐EL 3; SCR<br>☐EL 3; PRECLIN<br>☐EL 3; BR<br><br>No Evidence<br>☐EL 4; NE<br>☐EL 4: O	Cook RW, Middlebrook B, Wilkinson J, et al. Analytic validity of DecisionDx-Melanoma, a gene expression profile test for determining metastatic risk in melanoma patients. *Diagn Pathol.* Feb 13, 2018;13(1):13.<br><br>*Country:* USA	*Source:*<br>This study was sponsored by Castle Biosciences<br><br>7 authors: employees of Castle Biosciences<br><br>7 authors: hold stock in Castle Biosciences	*Methodology:*<br><br>Lab validation study<br><br>Inter-assay, inter-instrument, and inter-operator studies to evaluate reliability of test results, sample stability and reagent stability.	*Stated Objective:*<br>Assess reproducibility of Decision DX assay<br><br>☐Prospective<br>☒Retrospective<br><br>*Study Population and Setting:*<br><br>*N:* 8244 samples run (March 2013-June 2016)<br>168 samples run twice to assess reproducibility<br><br>168 specimens (de-identified of patient info)<br><br>*Intervention:* none<br><br>*Outcome Measures:*<br>Reproducibility of Decision Dx assay<br><br>*Follow-Up:*<br><br>*Notes:* Based on existing fixed tissue samples	*Results:*<br>Among all samples, the technical success of the assay was 98% in the 85% of samples that met pre-specified tumor content parameters.<br><br>Concordance among assays (in subset of samples (n=168) for which repeat assay was performed) on two consecutive days was 99%. Inter-instrumental concordance was 95%.<br><br>Analytic validity based on inter-assay, inter-operator, and inter-instrument reliability measurements show technical success of the test (99% rate).<br><br>*Notes:*	*Describe conclusions relative to question:*<br><br>The assay is reliable and reproduceable.<br><br>*Critiques of Methodology:*

Summary of Evidence Table<br>Question 1						
Level of Evidence*^<br><br>Choose one:	Study Citation<br>Author(s), Year, Title, Journal, Volume, Page #s	Funding Source	Study Methodology	Objective & Methods	Results	Conclusions
Strong<br>☐EL 1; RCT<br>☐EL 1; MRCT<br><br>Intermediate<br>☐EL 2; MNRCT<br>☐EL 2; NMA<br>☐EL 2; NRCT<br>☒EL 2; PCS<br>☐EL 2; RCCS<br>☐EL 2; NCCS<br>☐EL 2; CSS<br>☐EL 2; ES<br>☐EL 2; OLES<br>☐EL 2; PHAS<br><br>Weak<br>☐EL 3; DS<br>☐EL 3; ECON<br>☐EL 3; CCS<br>☐EL 3; SCR<br>☐EL 3; PRECLIN<br>☐EL 3; BR<br><br>No Evidence<br>☐EL 4; NE<br>☐EL 4: O	Dillon LD, Gadzia JE, Davidson RS. Prospective, Multicenter Clinical Impact Evaluation of a 31-Gene Expression Profile Test for Management of Melanoma Patients. *SKIN The Journal of Cutaneous Medicine*, 2018; 2(2): 111–121.<br><br>*Country:* USA	*Source:*<br>This study was sponsored by Castle Biosciences Inc. Participating institutions and investigators did not receive financial compensation for their involvement.<br><br>4 authors: employees Castle Biosciences<br><br>4 authors: hold stock options in Castle Biosciences	*Methodology*:<br><br>16 clinics with prospective follow-up (Dermatology, Surgical Oncology, Medical Oncology)<br><br>Management decisions made without GEP test then adjusted after GEP test available.<br><br>Median Breslow 1.1mm<br><br>60% SLN negative<br><br>(32% didn't have SLN at all)<br><br>73% were Class 1 GEP<br><br>Prospective Case-Series Cohort, Non-randomized	*Stated Objective:*<br>Prospectively evaluate the test impact on clinical management of patients<br><br>☒Prospective<br>☐Retrospective<br><br>*Study Population and Setting:*<br>Seven academic cancer centers<br><br>*N:* 247 from 16 different centers<br><br>*Intervention:* Compared management recs after GEP results to before<br><br>GEP<br><br>*Outcome Measures:* Change of decision-making<br><br>*Follow-Up:* None<br><br>*Notes:*	*Results:*<br>49% of pts had post GEP plans change<br><br>36% of Class 1 vs 86% of Class 2.<br><br>181/247 had class 1 GEP<br>66/247 had class 2<br>167 had SLNB; 149 were SLN positive (11%)<br>There was no indication which of the class I patients were SLN positive; 81% of the class II were SLN negative.<br><br>*Notes:*<br><br>No cross-table GEP versus pT stage.<br>SNB versus GEP result	*Describe conclusions relative to question:*<br><br>Physicians used GEP test results to change pt management, especially in Class 2 pts.<br><br>Claims that management change by GEP cannot be substantiated.<br>Relatively poor quality study with poor-quality data<br><br>*Critiques of Methodology:*<br>No evidence that the "right" decision was made based on change of management- no long term clinical follow-up of these pts.<br><br>Claims that change in management was based on GEP result, but

						the majority (68%) also had sentinel lymph node biopsy done.

	Summary of Evidence Table<br>Question 1, 2					
Level of Evidence*^<br><br>Choose one:	Study Citation<br>Author(s), Year, Title, Journal, Volume, Page #s	Funding Source	Study Methodology	Objective & Methods	Results	Conclusions
Strong<br>☐EL 1; RCT<br>☐EL 1; MRCT<br><br>Intermediate<br>☐EL 2; MNRCT<br>☐EL 2; NMA<br>☐EL 2; NRCT<br>☐EL 2; PCS<br>☐EL 2; RCCS<br>☐EL 2; NCCS<br>☐EL 2; CSS<br>☐EL 2; ES<br>☐EL 2; OLES<br>☐EL 2; PHAS<br><br>Weak<br>☐EL 3; DS<br>☐EL 3; ECON<br>☐EL 3; CCS<br>☐EL 3; SCR<br>☐EL 3; PRECLIN<br>☐EL 3; BR<br><br>No Evidence<br>☒EL 4; NE<br>☐EL 4: O	Farberg AS, Glazer AM, White R, Rigel DS. Impact of a 31-gene Expression Profiling Test for Cutaneous Melanoma on Dermatologists' Clinical Management Decisions. *J Drugs Dermatol*: 2017;16(5):428-431. (16)<br><br>*Country*: USA	*Source:*<br>None noted<br><br>2 authors=consultants to Castle Biosciences, Inc.<br><br>1=author participated in research fellowship partially funded by Castle Biosciences.	*Methodology:*<br><br>Gave Derm residents patient vignettes to assess clinical decision making, incorporating 31-GEP. They were given a presentation before on "clinical validity" for GEP.<br><br>Survey study of dermatology residents provided case-based scenarios involving management of 31-GEP results.	*Stated Objective:*<br>To determine impact of 31-GEP test on clinical decision making.<br><br>☒Prospective<br>☐Retrospective<br><br>*Study Population and Setting:*<br>Survey study of case-based scenarios provided to dermatology residents.<br><br>Derm residents at a national conference given clinical scenarios involving 31-GEP test (Stage IA, IB, IIA pts).<br><br>*N:* 169 dermatology resident physicians of 172 attendees responded to survey questions<br><br>*Intervention:* 31-GEP results given or not with clinical info.<br><br>Six cutaneous melanoma cases with GEP information varied.<br><br>*Outcome Measures:*<br>Management decisions (SLN bx, onc referral)<br><br>Clinical recommendation<br><br>*Follow-Up:* N/A<br><br>*Notes:*	*Results:*<br>Approximately 50% of respondents changed clinical decision making when faced with a Class II GEP result.<br><br>Most respondents gave 1.0 mm Breslow as guiding modality for SLNbx.<br><br>Basically states that when given 31-GEP results, specifically class 2 result, Breslow depth to guide SLNbx changed (more likely to refer thinner Breslow depth if high risk GEP class). Also more likely to recommend imaging.<br><br>*Notes:*	*Describe conclusions relative to question:*<br>"GEP result had a significant and appropriate impact on clinical decision making" (there was no evidence provided that this was appropriate).<br><br>The 31-GEP test had a "significant and appropriate" impact on management while remaining within established guidelines.<br><br>*Critiques of Methodology:*<br>Provides no information regarding validity of GEP<br><br>Were they trying to get the "right" answer based on who was presenting info to them?

						-Who gave the lecture on clinical validity of the test before the study??- that could significantly bias results<br>-Authors do not recognize these limitations in the manuscript<br>-They gave the residents an answer of “onc” and imaging referral which would not be currently recommended for the thin Breslow depths noted; confusing

	Summary of Evidence Table<br>Question 1, 2, 3					
Level of Evidence*^<br><br>Choose one:	Study Citation<br>Author(s), Year, Title, Journal, Volume, Page #s	Funding Source	Study Methodology	Objective & Methods	Results	Conclusions
**Strong**<br>☐EL 1; RCT<br>☐EL 1; MRCT<br><br>**Intermediate**<br>☐EL 2; MNRCT<br>☐EL 2; NMA<br>☐EL 2; NRCT<br>☐EL 2; PCS<br>☐EL 2; RCCS<br>☐EL 2; NCCS<br>☐EL 2; CSS<br>☐EL 2; ES<br>☐EL 2; OLES<br>☐EL 2; PHAS<br><br>**Weak**<br>☐EL 3; DS<br>☐EL 3; ECON<br>☐EL 3; CCS<br>☐EL 3; SCR<br>☐EL 3; PRECLIN<br>☐EL 3; BR<br><br>**No Evidence**<br>☒EL 4; NE<br>☐EL 4: O	Farberg AS, Marson JW, Glazer A, et al. Expert Consensus on the Use of Prognostic Gene Expression Profiling Tests for the Management of Cutaneous Melanoma: Consensus from the Skin Cancer Prevention Working Group. *Dermatol Ther.* 2022;12(4):807-823.<br>(81)<br><br>*Country:* USA	*Source:*<br>No funding or sponsorship was received for this study or publication of this article.<br><br>3 authors=consultants for Castle Biosciences<br><br>1 author=advisory board member and speaker for Castle Biosciences	*Methodology:*<br>Modified Delphi technique for consensus statements.<br><br>Review – no new data.<br><br>Skin Cancer Prevention Working Group are authors.	*Stated Objective:*<br>Assess applications for GEP in melanoma management.<br><br>☐Prospective<br>☐Retrospective<br><br>*Study Population and Setting:*<br><br>*N:*<br><br>*Intervention:*<br><br>*Outcome Measures:*<br><br>*Follow-Up:*<br><br>Notes:	*Results:*<br>Consensus statements support role for GEP above and beyond AJCC and NCCN.<br><br>*Notes:*<br>Consensus statements support role for GEP above and beyond AJCC and NCCN.<br>The statements are non-specific.	*Describe conclusions relative to question:*<br><br>Consensus statements support role for GEP.<br><br>"GEP tests provide additional, reproducible information for dermatologists to consider within the larger framework of the eighth edition of the AJCC and NCCN cutaneous melanoma guidelines when counseling regarding prognosis and when considering a sentinel lymph node biopsy."<br><br>*Critiques of Methodology:*<br>No formal accompanying systematic review/meta-analysis to support the consensus

						process (only 8 panelists).<br><br>Review of existing data.

	Summary of Evidence Table<br>Question 1, 2					
Level of Evidence*^<br><br>Choose one:	Study Citation<br>Author(s), Year, Title, Journal, Volume, Page #s	Funding Source	Study Methodology	Objective & Methods	Results	Conclusions
☐EL 1; RCT<br>☐EL 1; MRCT<br>Strong<br><br>☐EL 2; MNRCT<br>☐EL 2; NMA<br>☐EL 2; NRCT<br>☐EL 2; PCS<br>☐EL 2; RCCS<br>☐EL 2; NCCS<br>☐EL 2; CSS<br>☐EL 2; ES<br>☐EL 2; OLES<br>☐EL 2; PHAS<br>Intermediate<br><br>☐EL 3; DS<br>☐EL 3; ECON<br>☒EL 3; CCS<br>☐EL 3; SCR<br>☐EL 3; PRECLIN<br>☐EL 3; BR<br>Weak<br><br>☐EL 4; NE<br>☐EL 4: O<br>No Evidence	Greenhaw BN, Zitelli JA, Brodland DG. Estimation of Prognosis in Invasive Cutaneous Melanoma: An Independent Study of the Accuracy of a Gene Expression Profile Test. *Dermatol Surg*. Dec 2018;44(12):1494-1500.<br><br>*Country:* United States	*Source:* None<br><br>The authors have indicated no significant interest with commercial supporters.	*Methodology:* Retrospective review of all patients in a single center prospective database.<br><br>Retrospective analysis of selected patients in institutional database.<br><br>Retrospective analysis of institutional database of patients undergoing 31-GEP testing supplemented with additional cases identified as having metastatic disease whose primary tumors were then retrieved for 31-GEP if not done previously	*Stated Objective:*<br><br>☐Prospective<br>☒Retrospective<br><br>*Study Population and Setting:* Single center, referral practice<br><br>Patients treated for CM within the last five years and undergone GEP testing. Clinical, histopathologic, and outcomes data were analyzed. A subcohort of patients with known metastatic disease were identified and tested.<br><br>*N:* 256<br><br>*Intervention:* GEP on all patients prospectively 31-GEP<br><br>*Outcome Measures:* Recurrent free survival and patterns<br>Metastasis at mean f/u 23 months<br><br>*Follow-Up:* Median <2 years<br>Mean 23 months<br><br>*Notes:*	*Results:*<br>13 patients developed metastases, GEP identified 77% of these.<br><br>99% NPV of the GEP "only 3 of the 214 Class I patients metastasized."<br><br>24 of 37 stage II patients were Class II and 10 metastasized, (not surprising), on 42%.<br><br>2 of 13 stage II that were Class I metastasized.<br><br>Of 256: 214 (84%) Class 1, (193 Class 1A, 21 Class 1B)<br>42 (16%) Class 2, (16 Class 2A, 26 Class 2B)<br><br>Of 256: 13 developed metastasis (10 of 13 Class 2 on testing).<br><br>3-year MFS rate 98%for Class 1 patients and 74% for Class 2 patients, with 5-year MFS rates of 93% and 69%, respectively ($p < .00001$)<br><br>Notes: No comparison based on histologic staging beyond stage I and Stage II (no substaging comparisons).	*Describe conclusions relative to question:*<br><br>The goal of this study was to validate GEP as an alternative to SLN bx for prognostication. While not explicitly stated, that becomes evident in the rhetoric. The study simply validates the prognostic value of GEP retrospectively in a cohort of patients. It does not analyze whether it is discerning when compared to traditional histologic staging methodologies.<br><br>Authors conclude 31-GEP is prognostic and can guide care.<br><br>*Critiques of Methodology:* Quoted from the paper:<br><br>*At the time of initial CM diagnosis, 219 (86%) of the tested tumors were Stage I. None of the 18 Stage I Class*

						2 tumors metastasized, whereas 1 (0.5%) of 201 Stage I Class 1 tumors metastasized.<br><br>This implies that that the GEP is not discerning in Stage I patients since Class differentiation did not predict any pattern of disease recurrence.<br><br>Discussion trying to explain why their findings might not reflect the ultimate utility of the GEP.<br><br>Retrospective<br>Enriched for subsequent testing after recurrence in some of the patients, number of patients in this subcohort nor method for identification not stated<br><br>With median f/u 23 months 5 yr KM survival analysis seems invalid.<br><br>Class 1 median Breslow 0.6 mm vs Class 2 = 2.2 mm<br><br>No analysis comparing value of 31-GEP beyond AJCC stage, Breslow Depth

	Summary of Evidence Table<br>Question 1,2,3					
Level of Evidence*^<br><br>Choose one:	Study Citation<br>Author(s), Year, Title, Journal, Volume, Page #s	Funding Source	Study Methodology	Objective & Methods	Results	Conclusions
☐EL 1; RCT<br>☐EL 1; MRCT<br>Strong<br><br>☐EL 2; MNRCT<br>☐EL 2; NMA<br>☐EL 2; NRCT<br>☐EL 2; PCS<br>☐EL 2; RCCS<br>☐EL 2; NCCS<br>☐EL 2; CSS<br>☐EL 2; ES<br>☐EL 2; OLES<br>☐EL 2; PHAS<br>Intermediate<br><br>☐EL 3; DS<br>☐EL 3; ECON<br>☐EL 3; CCS<br>☐EL 3; SCR<br>☐EL 3; PRECLIN<br>☐EL 3; BR<br>Weak<br><br>☒EL 4; NE<br>☐EL 4: O<br>No	Grossman D, Okwundu N, Bartlett EK, et al. Prognostic Gene Expression Profiling in Cutaneous Melanoma: Identifying the Knowledge Gaps and Assessing the Clinical Benefit. *JAMA Dermatol*. 2020;156(9):1004-1011.<br>(61)<br><br>*Country:* United States	*Source:* Supported by the University of Utah Department of Dermatology, the Huntsman Cancer Foundation at the University of Utah, the Melanoma Center at the Huntsman Cancer Institute, the Hope Foundation, the National Health and Medical Research Council of Australia Program, fellowship grants and grants from the American Skin Association and the Sydney Medical School Foundation, This material is the result of work supported	*Methodology:*<br><br>Consensus statement	*Stated Objective:* Develop guidelines within the national Melanoma Prevention Working Group (MPWG) on integration of GEP testing into management of patients with CM.<br>1. Review of published data using GEP tests<br>2. Definition of acceptable performance criteria<br>3. Current recommendations of use of GEP in clinical practice<br>4. Considerations for future studies<br><br>☐Prospective<br>☒Retrospective<br><br>Study Population and Setting:<br><br>N: N/A<br><br>Intervention: Consensus guideline<br><br>MPWG members and other international specialists, 2 surveys<br><br>Outcome Measures: N/A	*Results:*<br><br>The MPWG members are optimistic about the future use of prognostic GEP testing to improve risk stratification and enhance clinical decision-making but acknowledge that current utility is limited by test performance in patients with stage I disease. Published studies of GEP testing have not evaluated results in the context of all relevant clinicopathologic factors or as predictors of regional nodal metastasis to replace sentinel lymph node biopsy (SLNB). The performance of GEP tests has generally been reported for small groups of patients representing particular tumor stages or in aggregate form, such that stage-specific performance cannot be ascertained, and without survival outcomes compared with data from the American Joint Committee on Cancer 8th edition melanoma staging system international database. There are significant challenges to performing clinical trials incorporating GEP testing	*Describe conclusions relative to question:* Enthusiasm for the concept, but limited test performance in stage I disease. Published studies not evaluated results in context of all relevant clinicopathologic factors or as predictors of regional nodal metastasis to replace SLNB.<br><br>More studies are needed to determine clinical utility of GEP.<br><br>*Critiques of Methodology:*<br><br>Consensus guideline was performed reasonably.

		with resources and use of facilities at the Veterans Affairs Palo Alto Health Care System in Palo Alto CA. Contents do not represent the views of the US Dept of Veterans Affairs or the US Gov't. The funders had no role in the design and conduct of the study; collection, management, analysis and interpretation of data, preparation, review or approval of the manuscript; and decision to submit for publication.<br><br>1 author: nonfinancial support from Castle Biosciences outside submitted work.<br><br>1 author: grants from Castle Biosciences outside		Follow-Up:<br><br>Notes:	with SLNB and adjuvant therapy. The MPWG members favor conducting retrospective studies that evaluate multiple GEP testing platforms on fully annotated archived samples before embarking on costly prospective studies and recommend avoiding routine use of GEP testing to direct patient management until prospective studies support their clinical utility.<br><br>Notes:	

		submitted work<br><br>1 author: personal fees outside the submitted work Castle Biosciences<br><br>1 author: personal fees from Neracare outside submitted work<br><br>1 author: manuscripts and abstracts published using the test with company support of the assay—all publications were peer review and no personal of institutional payment or compensation was rec'd. Castle Biosciences<br><br>1 author: served as an investigator for Castle Biosciences (no personal financial compensation)				

Summary of Evidence Table<br>Question 1						
Level of Evidence*^<br><br>Choose one:	Study Citation<br>Author(s), Year, Title, Journal, Volume, Page #s	Funding Source	Study Methodology	Objective & Methods	Results	Conclusions
Strong<br>☐EL 1; RCT<br>☐EL 1; MRCT<br><br>Intermediate<br>☐EL 2; MNRCT<br>☐EL 2; NMA<br>☐EL 2; NRCT<br>☐EL 2; PCS<br>☒EL 2; RCCS<br>☐EL 2; NCCS<br>☐EL 2; CSS<br>☐EL 2; ES<br>☐EL 2; OLES<br>☐EL 2; PHAS<br><br>Weak<br>☐EL 3; DS<br>☐EL 3; ECON<br>☐EL 3; CCS<br>☐EL 3; SCR<br>☐EL 3; PRECLIN<br>☐EL 3; BR<br><br>No Evidence<br>☐EL 4; NE<br>☐EL 4: O	Hieken TJ, Sadurni MB, Quattrocchi E, et al. Using the Merlin Assay for Reducing Sentinel Lymph Node Biopsy Complications in Melanoma: A Retrospective Cohort Study. *Int J Dermatol* 2022;61(7):848-854. (83)<br><br>*Country*: USA	*Source:*<br><br>National Cancer Institute grant (K08 CA215105) and Melanoma Research Alliance award (652760)<br><br>2 authors: SkylineDx employees<br><br>2 authors that hold stock options in SkylineDx<br><br>1 author: research funding from SkylineDx to institution<br><br>1 author: research funding<br><br>1 author financial interest in Merlin assay	*Methodology:* Retrospective cohort study.<br><br>Review SLN bx complications in the context of GEP likelihood for negative SLN.	*Stated Objective:* Complications from SLNB<br><br>"Here, we gauged the potential of the Merlin assay to reduce SLNB-associated complications. The Merlin assay uses clinicopathologic variables and tumor gene expression profiling to identify low-risk patients who may forgo SLNB."<br><br>☐Prospective<br>☒Retrospective<br><br>*Study Population and Setting:* Single institution study;University<br><br>*N*: 558<br>Merlin assay results available in 547<br><br>*Intervention:* SLNB biopsy<br><br>*Outcome Measures:* Complication rates of SLNB and possibility of avoidance.<br><br>*Follow-Up:* cases from 2004 - 2018<br>Median follow up not stated<br><br>*Notes:*<br>These patients represent a subcohort of a previously published report on CP-GEP	*Results:*<br>17.4% complication rate.<br>If used Merlin to determine who not to perform SLN bx theoretically would have reduced rate by 59.2%.<br><br>*Notes:*	*Describe conclusions relative to question:*<br><br>Did not evaluate the efficacy or accuracy of Merlin assay.<br><br>*Critiques of Methodology:*<br><br>This is not a CP-GEP study.<br><br>This is theoretical construct.<br><br>Single institution (albeit 3 sites).

	Summary of Evidence Table<br>Question 1, 2					
Level of Evidence*^<br><br>Choose one:	Study Citation<br>Author(s), Year, Title, Journal, Volume, Page #s	Funding Source	Study Methodology	Objective & Methods	Results	Conclusions
**Strong**<br>☐EL 1; RCT<br>☐EL 1; MRCT<br><br>**Intermediate**<br>☐EL 2; MNRCT<br>☐EL 2; NMA<br>☐EL 2; NRCT<br>☐EL 2; PCS<br>☐EL 2; RCCS<br>☐EL 2; NCCS<br>☐EL 2; CSS<br>☐EL 2; ES<br>☐EL 2; OLES<br>☒EL 2; PHAS<br><br>**Weak**<br>☐EL 3; DS<br>☐EL 3; ECON<br>☐EL 3; CCS<br>☐EL 3; SCR<br>☐EL 3; PRECLIN<br>☐EL 3; BR<br><br>**No Evidence**<br>☐EL 4; NE<br>☐EL 4: O	Jarell A, Gastman BR, Dillon LD, et al. Optimizing Treatment Approaches for Patients With Cutaneous Melanoma by Integrating Clinical and Pathologic Features with the 31-Gene Expression Profile Test. *J Am Acad Dermtol.*. 2022. 87(6):<br>(85): 1312-1320.<br><br>*Country:* USA	*Source:*<br>Funded by Castle Biosciences, Inc.<br><br>8 authors: Castle Bioscience employees<br><br>8 authors: stock and options holders for Castle Biosciences<br><br>2 authors: speakers bureau for Castle Biosciences	*Methodology:*<br>Algorithm development.<br><br>Analysis of existing datasets.<br><br>“The cohort comprised 2104 patients from four independent centers (n = 548) and a previously published meta-analysis that combined two retrospectively tested (n = 990) and two prospectively tested (n = 566) cohorts.	*Stated Objective:* Risk of recurrence.<br><br>“To develop an algorithm by integrating the 31-gene expression profile test with clinicopathologic data for an optimized, personalized risk of recurrence (integrated 31 risk of recurrence [i31-ROR]) or death and use i31-ROR in conjunction with a previously validated algorithm for precise sentinel lymph node positivity risk estimates (i31-SLNB) for optimized treatment plan decisions.”<br><br>☐Prospective<br>☒Retrospective<br><br>*Study Population and Setting*:<br>Multicenter cohort<br>See methodology<br><br>*N:* 1581 development cohort; 523 validation cohort<br><br>Intervention:<br>31-GEP<br>AI<br><br>*Outcome Measures:*<br>Recurrence as measured by a personalized risk of recurrence (integrated 31 risk of recurrence (i31-ROR)).<br><br>RFS, DMFS, Mel Sp Survival	*Results:*<br>I31-ROR risk (low vs high) may give personalized prediction of recurrence (RFS, DMFS, MSS) risk and SLNB results.<br><br>Mathematical modeling including GEP improved prediction of RFS, MSS and DMFS. Also analyzed SLN vs model for outcomes.<br><br>*Notes:*	*Describe conclusions relative to question:*<br><br>Algorithm incorporating GEP may give personalized prediction of recurrence risk and SLNB results.<br><br>Patient risk can be analyzed more comprehensively, resulting in better patient care. We further showed that the number of patients undergoing SLNB could be reduced,”<br><br>Mathematical modeling with no clinical testing.<br><br>*Critiques of Methodology*<br><br>SLNB was only taken into account post hoc.<br>:

				Follow-Up: n/a<br>unclear		

	Summary of Evidence Table<br>Question 1					
Level of Evidence*^<br><br>Choose one:	Study Citation<br>Author(s), Year, Title, Journal, Volume, Page #s	Funding Source	Study Methodology	Objective & Methods	Results	Conclusions
Strong<br>☐EL 1; RCT<br>☐EL 1; MRCT<br><br>Intermediate<br>☐EL 2; MNRCT<br>☐EL 2; NMA<br>☐EL 2; NRCT<br>☐EL 2; PCS<br>☒EL 2; RCCS<br>☐EL 2; NCCS<br>☐EL 2; CSS<br>☐EL 2; ES<br>☐EL 2; OLES<br>☐EL 2; PHAS<br><br>Weak<br>☐EL 3; DS<br>☐EL 3; ECON<br>☐EL 3; CCS<br>☐EL 3; SCR<br>☐EL 3; PRECLIN<br>☐EL 3; BR<br><br>No Evidence<br>☐EL 4; NE<br>☐EL 4: O<br><br>RCCS	Johansson I, Tempel D, Dwarkasing JT, et al. Validation of a Clinicopathological and Gene Expression Profile Model to Identify Patients with Cutaneous Melanoma Where Sentinel Lymph Node Biopsy is Unnecessary. *Eurn J Surg Oncol.* 2022;48(2):320-325 (86)<br><br>*Country:* Sweden	*Source:*<br>Skyline DX<br>Knut and Alice Wallenberg Foundation Wallenberg Centre for Molecular and Translational Medicine, University of Gothenburg, Sweden<br><br>"Analysis and costs associated with the study were covered by SkylineDx based on a per-patient fee. The analyses were blinded and SkylineDx did not have any impact on the decision to publish or on what data to publish. Additional funding was given by the Knut and Alice Wallenberg Foundation,Wallenberg Centre for Molecular and Translational Medicine, University of Gothenburg, Sweden."<br><br>3 authors: SkylineDx employees<br><br>3 authors: option holders of SkylineDx<br><br>1 author: rec'd research grants from SkylineDx	*Methodology:* retrospective cohort study<br><br>GEP testing of previous patients. Validation cohort.	*Stated Objective:*<br>Prediction of SLN status.<br><br>Identify patients that could forgo SLN bx.<br><br>☐Prospective<br>☒Retrospective<br><br>*Study Population and Setting:*<br>Multi-center Swedish cohort<br><br>University with prospective database<br><br>*N:* 421<br><br>*Intervention:* CP-GEP<br><br>Testing of archival tissues.<br><br>*Outcome Measures:* NPV prediction of SLN status<br><br>*Follow-Up:* n/a<br>: patients from 2006-2014<br><br>*Notes:*	*Results:*<br>NPV 96.5% in T1/2 patients.<br><br>35% of low risk T1/2 patients (based on CP-GEP) could forego SLNB.<br><br>Analysis included cross-tab comparison of other prediction models.<br><br>"The SLNB positivity rate was 13%. Of 421 primary melanomas, the CP-GEP model identified 86 patients as having a low risk for nodal metastasis. In patients with pT1-2 melanomas, the SLNB reduction rate was 35.4% (95% CI: 29.4e41.8) with a negative predictive value (NPV) of 96.5% (95% CI: 90.0e99.3). Among patients with pT1-3 melanomas, CP-GEP suggested a SLNB reduction rate of 24.0% (95% CI: 19.7 e28.8) and a NPV of 96.5% (95% CI: 90.1e99.3). Only one of 118 pT3 tumors was classified as CP-GEP Low Risk, and all pT4 tumors were classified as being high risk for nodal metastasis."<br><br>*Notes:*	*Describe conclusions relative to question:*<br><br>CP-GEP can help predict SLN status.<br><br>Model can identify lower risk patients.<br><br>*Critiques of Methodology:*<br>*Methodology:* no f/u to account for false negative results.<br><br>Note that GEP was actually not meant to predict SLN status.<br><br>Retrospective analysis.<br><br>Single institution.

Level of Evidence*^<br><br>Choose one:	Summary of Evidence Table<br>Question 1, 2<br><br>Study Citation<br>Author(s), Year, Title, Journal, Volume, Page #s	Funding Source	Study Methodology	Objective & Methods	Results	Conclusions
Strong<br>☐EL 1; RCT<br>☐EL 1; MRCT<br><br>Intermediate<br>☐EL 2; MNRCT<br>☐EL 2; NMA<br>☐EL 2; NRCT<br>☐EL 2; PCS<br>☐EL 2; RCCS<br>☐EL 2; NCCS<br>☐EL 2; CSS<br>☐EL 2; ES<br>☐EL 2; OLES<br>☐EL 2; PHAS<br><br>Weak<br>☐EL 3; DS<br>☐EL 3; ECON<br>☐EL 3; CCS<br>☐EL 3; SCR<br>☐EL 3; PRECLIN<br>☐EL 3; BR<br><br>No Evidence<br>☒EL 4; NE<br>☐EL 4: O	Kashani-Sabet M, Leachman SA, Stein JA, et al. Early Detection And Prognostic Assessment Of Cutaneous Melanoma: Consensus On Optimal Practice And The Role Of Gene Expression Profile Testing. *JAMA Dermatol.* 2023;159(5):545-553.<br><br>*Country:* USA	*Source:*<br><br>The funding for the administration and facilitation of the consensus development conference and the development of this manuscript was provided by Dermtech Inc in an unrestricted award that was administratively overseen by the Melanoma Research Foundation and managed and executed at UPMC by the principal investigator (Dr Kirkwood). DermTech was not involved in the preparation or editing of the manuscript.<br><br>The funding organizations had no role in the design and	*Methodology:* Modified Delphi consensus used for case scenarios asked of 60 melanoma panelists followed by a consensus conference (n=51)	*Stated Objective:* “To provide consensus recommendations on optimal screening practices and prebiopsy diagnostic, postbiopsy diagnostic, and prognostic assessment of cutaneous melanoma.”<br><br>☐Prospective<br>☐Retrospective<br><br>*Study Population and Setting:* consensus conference<br><br>*N*: 60 melanoma panelists, 42 survey respondents, 51 panelists at consensus conference<br><br>*Intervention:* Modified Delphi consensus<br><br>*Outcome Measures:* N/A<br><br>*Follow-Up:*<br><br>*Notes:*	*Results:*<br><br>Consensus (>70% agreement) low-risk GEP score should not outweigh histologic features when determining eligibility for SLNB.<br><br>No consensus on imaging recommendations in the setting of high-risk prognostic GEP score and low-risk histology and/or negative SLN.<br><br><10% of experts recommended post biopsy GEP in management.<br><br>*Notes*: Cite need for additional studies	*Describe conclusions relative to question:*<br><br>No consensus on role of GEP testing in clinical decision making.<br><br>“1 area in which consensus was reached among the panel was that the31-Gene prognostic GEP testing result alone would not outweigh routine histopathologic features to inform selection of patients for SLNB. Panelists supported an approach that favors histopathologic vs GEP testing for SLNB. As shown in Table 4, the panelists consistently recommended WLE plus SLNB for individuals who meet histopathologic criteria for SLNB, even in the setting of a low-risk (class 1) 31-gene GEP testing result. Panelists were not queried regarding various SLNB risk calculators/ nomograms or other GEP testing (ie, Merlin Assay 8-GEP; DecisionDx

		conduct of the study; collection, management, analysis, and interpretation fo the data; preparation, review, or approval of the manuscript and decision to submit the manuscript for publication.<br><br>2 authors: research support from Castle Biosciences outside this project<br><br>1 author: research support from Castle both during and outside this work<br><br>1 author: grant support from Skyline Dx outside this work<br><br>1 author: nonfinancial support from SkylineDx outside the work				i-31-GEP) purported to predict SLN positivity."<br><br>*Critiques of Methodology:*<br><br>Other than funding support, no concerns. Appropriately designed and performed consensus conference.

	Summary of Evidence Table<br>Question 1, 2					
Level of Evidence*^<br><br>Choose one:	Study Citation<br>Author(s), Year, Title, Journal, Volume, Page #s	Funding Source	Study Methodology	Objective & Methods	Results	Conclusions
**Strong**<br>☐EL 1; RCT<br>☐EL 1; MRCT<br><br>**Intermediate**<br>☒EL 2; MNRCT<br>☐EL 2; NMA<br>☐EL 2; NRCT<br>☐EL 2; PCS<br>☐EL 2; RCCS<br>☐EL 2; NCCS<br>☐EL 2; CSS<br>☐EL 2; ES<br>☐EL 2; OLES<br>☐EL 2; PHAS<br><br>**Weak**<br>☐EL 3; DS<br>☐EL 3; ECON<br>☐EL 3; CCS<br>☐EL 3; SCR<br>☐EL 3; PRECLIN<br>☐EL 3; BR<br><br>**No Evidence**<br>☐EL 4; NE<br>☐EL 4: O	Litchman GH, Prado G, Teplitz RW, & Rigel D (2020). A Systematic Review and Meta-Analysis of Gene Expression Profiling for Primary Cutaneous Melanoma Prognosis. *SKIN The Journal of Cutaneous Medicine.* 4(3): 221–237.<br><br>*Country:* USA	*Source:* None<br><br>1 author: consultant for Castle Biosciences<br><br>2 authors: are fellows of the National Society for Cutaneous Medicine which received grants from Castle Biosciences	*Methodology:* Meta-analysis of six Decision DX studies and systematic review of other GEP.<br><br>Systematic review and meta-analysis.	*Stated Objective:*<br><br>☐Prospective<br>☒Retrospective Meta-analysis/systematic review<br><br>*Study Population and Setting:*<br><br>*N:* 29 studies with 8 gene signatures; 6 with 31-gene signature—meta-analysis performed on the 6.<br><br>*Intervention:*<br><br>Outcome Measures: Prognostic validity, analytic validity and clinical impact of GEP.<br><br>*Follow-Up:*<br><br>*Notes:* 29 articles from systematic review (9 unique gene signatures);<br>6 articles used for the meta-analysis (limited to 1 GEP [31-gene signature])	*Results:* In meta-analysis of 31-gene signature, pooled OR for recurrence was 9.42 and for distant metastases 7.93; pooled OR for OS was 6.43 and for SLN positivity 2.99.<br><br>Pooled OR for SLNB positivity = 2.99 (95% CI: 2.15-4.15).<br><br>Performance with other outcomes was slightly better (recurrence, distant metastases, overall survival).<br><br>*Notes*:	*Describe conclusions relative to question:*<br><br>Results of the study may aid clinicians in using GEP assays and in managing patients with melanoma.<br><br>Pooled OR for SLNB positivity = 2.99 (95% CI: 2.15-4.15).<br><br>Limited meta-analysis (reflecting heterogeneous data).<br><br>*Critiques of Methodology:*<br><br>Significant heterogeneity in studies included for systematic review and meta-analysis.<br><br>Limited meta-analysis (reflecting heterogeneous data from 4 fairly dated studies).

	Summary of Evidence Table<br>Question 1					
Level of Evidence*^<br><br>Choose one:	Study Citation<br>Author(s), Year, Title, Journal, Volume, Page #s	Funding Source	Study Methodology	Objective & Methods	Results	Conclusions

☐EL 1; RCT<br>☐EL 1; MRCT<br>(Strong)<br><br>☐EL 2; MNRCT<br>☐EL 2; NMA<br>☐EL 2; NRCT<br>☐EL 2; PCS<br>☐EL 2; RCCS<br>☐EL 2; NCCS<br>☐EL 2; CSS<br>☐EL 2; ES<br>☐EL 2; OLES<br>☐EL 2; PHAS<br>(Intermediate)<br><br>☒EL 3; DS<br>☐EL 3; ECON<br>☐EL 3; CCS<br>☐EL 3; SCR<br>☐EL 3; PRECLIN<br>☐EL 3; BR<br>(Weak)<br><br>☐EL 4; NE<br>☐EL 4: O<br>(No Evidence)	Marchetti MA, Dusza SW, Bartlett EK. Utility of a Model for Predicting the Risk of Sentinel Lymph Node Metastasis in Patients With Cutaneous Melanoma. *JAMA Dermatol.* 2022;158(6):680-683 (89)<br><br>*Country:* USA	*Source:*<br>This research was funded in part through the Memorial Sloan Kettering Cancer Center institutional National Institutes of Health/National Cancer Institute Cancer Center Support Grant P30 CA008748. The NIH had no role in the design and conduct of the study; collection, k management, analysis, and interpretation of the data, preparation, review, or approval of the manuscript; and decision to submit the manuscript for publication.<br><br>1 author: institutional research support from SkylineDx outside the submitted work	*Methodology:*<br>Decision analytic study based on i31-GEP model (presented as citation #78, Whitman et al.).<br><br>Use of mathematical model on existing dataset.<br><br>"Raw data and classification measures of the i31-GEP-SLNB model by American Joint Committee on Cancer (AJCC) T category were extracted from Whitman et al2 for patients with T1a-HR, T1b, and T2 disease;"	*Stated Objective:* "To determine if use of the i31-GEP-SLNB model is associated with clinical benefit when used to select patients for SLN biopsy."<br><br>☐Prospective<br>☒Retrospective<br><br>*Study Population and Setting:* See methodology.<br><br>*N:* 1097<br><br>*Intervention:* Use of mathematical modeling on existing dataset.<br><br>*Outcome Measures:* "To determine if use of the i31-GEP-SLNB model is associated with clinical benefit when used to select patients for SLN biopsy."<br><br>*Follow-Up:* Not stated<br><br>*Notes:*	*Results:*<br>"Compared with other SLN biopsy selection strategies, use of the i31-GEP-SLNB model had greater net benefit for patients with T1b (+0.012), T2a (+0.002), and T2b melanoma (+0.002) but not for those with high-risk T1a (−0.003) disease. The improvement in relative utility was +22%in patients with T1b, +1% in T2a, and +2%in T2b melanoma."<br><br>*Notes:*	*Describe conclusions relative to question:*<br><br>Appropriate<br><br>*Critiques of Methodology:*<br>Should not be included as source GEP article<br><br>Computational modeling

	Summary of Evidence Table<br>Question 1, 2					
Level of Evidence*^<br><br>Choose one:	Study Citation<br>Author(s), Year, Title, Journal, Volume, Page #s	Funding Source	Study Methodology	Objective & Methods	Results	Conclusions

Strong<br>☐EL 1; RCT<br>☐EL 1; MRCT<br><br>Intermediate<br>☐EL 2; MNRCT<br>☐EL 2; NMA<br>☐EL 2; NRCT<br>☐EL 2; PCS<br>☐EL 2; RCCS<br>☐EL 2; NCCS<br>☐EL 2; CSS<br>☐EL 2; ES<br>☐EL 2; OLES<br>☐EL 2; PHAS<br><br>Weak<br>☐EL 3; DS<br>☐EL 3; ECON<br>☐EL 3; CCS<br>☐EL 3; SCR<br>☐EL 3; PRECLIN<br>☐EL 3; BR<br><br>No Evidence<br>☒EL 4; NE<br>☐EL 4: O	Mirsky R, Prado G, Svoboda R, Glazer A, Rigel D. Management Decisions Made by Physician Assistants and Nurse Practitioners in Cutaneous Malignant Melanoma Patients: Impact of a 31-Gene Expression Profile Test. *J Drugs Dermatol* 2018;17(11):1220-1223<br>(31)<br><br>*Country:* USA	*Source:*<br>None stated<br><br>1 author: consultant to Castle Biosciences.<br><br>3 authors: participated in a research fellowship which was partially funded by Castle Biosciences	*Methodology:*<br>Questionnaire survey.<br>Hypothetical management.<br><br>Online survey given to PA/NPs with six melanoma patient vignettes.<br>Effect of lower and higher risk 31-GEP test results on management measured.	*Stated Objective:*<br>Change in practice behavior.<br><br>To determine the impact of 31-GEP test results on management decisions made by dermatology PA/NPs for CM patients.<br><br>Survey study<br><br>*Study Population and Setting:*<br>Nurse Practitioners and Physician Assistants<br><br>NPs and Pas – questionnaires sent<br><br>*N:* 164<br><br>*Intervention:* Online survey with vignettes of pt and 31-GEP results.<br><br>Questionnaires regarding practice.<br><br>*Outcome Measures:*<br>-Proportion PA/NPs alter recommendation SLNBx.<br>-Follow-up imaging and intervals recommended.<br><br>Referral rates for SNB and radiology.<br><br>*Follow-Up*:<br>none<br>______<br>*Notes:*	*Results:*<br><br>Lower risk 31-GEP led to statistically significant decrease in recommended SLNBx, imaging or quarterly follow-up.<br><br>Low risk – decreased referral rate for SNB.<br><br>High risk – increased rate of referral for SNB and radiology.<br>All hypothetical – no patients involved.<br>______<br>*Notes:*	*Describe conclusions relative to question:*<br><br>31-GEP results significantly impact management decisions by dermatology PA/NPs.<br><br>Routine use of GEP would change patient management.<br><br>*Critiques of Methodology:*<br><br>-Survey of GEP on APP management does not evaluate the validity of the test.<br>-Was survey voluntary or was there an incentive.

	Summary of Evidence Table<br>Question 1					
Level of Evidence*^<br><br>Choose one:	Study Citation<br>Author(s), Year, Title, Journal, Volume, Page #s	Funding Source	Study Methodology	Objective & Methods	Results	Conclusions
☐EL 1; RCT<br>☐EL 1; MRCT<br><br>☐EL 2; MNRCT<br>☐EL 2; NMA<br>☐EL 2; NRCT<br>☐EL 2; PCS<br>☒EL 2; RCCS<br>☐EL 2; NCCS<br>☐EL 2; CSS<br>☐EL 2; ES<br>☐EL 2; OLES<br>☐EL 2; PHAS<br><br>☐EL 3; DS<br>☐EL 3; ECON<br>☐EL 3; CCS<br>☐EL 3; SCR<br>☐EL 3; PRECLIN<br>☐EL 3; BR<br><br>☐EL 4; NE<br>☐EL 4: O	Mulder E, Dwarkasing JT, Tempel D, et al. Validation of a clinicopathological and gene expression profile model for sentinel lymph node metastasis in primary cutaneous melanoma. *Br J Dermatol*. 2021;184(5):944-951. (75)<br><br>*Country:* Netherlands	*Source:*<br><br>This work was supported by the National Enterprising Netherlands Agency.<br><br>4 authors: employees of SkylineDx<br><br>4 authors are options holders of SkylineDx	*Methodology:* Retrospective single institution cohort study.<br><br>Validation cohort.	*Stated Objective:* GEP prediction of nodal positivity.<br><br>Determine predictive value of CP-GEP on SLN metastasis.<br><br>☐Prospective<br>☒Retrospective<br><br>*Study Population and Setting:* Retrospective single institution cohort study T1-T4 included.<br><br>University<br><br>*N:* 210<br><br>*Intervention:* GEP testing of treated patients<br><br>*Outcome Measures:* Nodal metastases<br><br>SLN positivity<br><br>*Follow-Up:* n/a<br>Patients operated upon 2007-2017<br><br>*Notes:*	*Results:*<br><br>NPV is 90.5%.<br><br>"Overall, the CP-GEP model had a negative predictive value (NPV) of 90.5% [95% confidence interval (CI) 77.9–96.2], with an NPV of 100% (95% CI 722–100) in T1, 89.3% (95%CI 728–963) in T2 and 75.0% (95% CI 301–954) in T3 melanomas. The CP-GEP indicated high risk in all T4 melanomas."<br><br>*Notes:*	*Describe conclusions relative to question:* Low risk based on GEP may be able to predict negative SLNB.<br><br>Supported.<br><br>*Critiques of Methodology:*<br><br>Not sure the conclusion is supported by the data presented; noted that GEP was actually not meant to predict SLN status; no f/u to account for false negative results.<br><br>Retrospective<br><br>Single Institution<br><br>Few T1 patients with few events<br><br>"Because the algorithm has been bridged to a different platform (Quantstu-dioDx), this study does not directly validate the development platform (Fluidigm)."

Summary of Evidence Table<br>Question 1, 2						
Level of Evidence*^<br><br>Choose one:	Study Citation<br>Author(s), Year, Title, Journal, Volume, Page #s	Funding Source	Study Methodology	Objective & Methods	Results	Conclusions
Strong<br>☐EL 1; RCT<br>☐EL 1; MRCT<br><br>Intermediate<br>☐EL 2; MNRCT<br>☐EL 2; NMA<br>☐EL 2; NRCT<br>☐EL 2; PCS<br>☒EL 2; RCCS<br>☐EL 2; NCCS<br>☐EL 2; CSS<br>☐EL 2; ES<br>☐EL 2; OLES<br>☐EL 2; PHAS<br><br>Weak<br>☐EL 3; DS<br>☐EL 3; ECON<br>☐EL 3; CCS<br>☐EL 3; SCR<br>☐EL 3; PRECLIN<br>☐EL 3; BR<br><br>No Evidence<br>☐EL 4; NE<br>☐EL 4: O	Mulder E, Johansson I, Grunhagen DJ, et al. Using a Clinicopathologic and Gene Expression (CP-GEP) Model to Identify Stage I-II Melanoma Patients at Risk of Disease Relapse. Cancers. 2022;14(12):2854. (92)<br><br>*Country*<br>Netherlands, Sweden	*Source*: This work was supported by the Netherlands Enterprise Agency (IK18007).<br><br>3 authors: SkylineDx employees<br><br>3 authors: options stakeholders of SkylineDx<br><br>3 authors: rec'd two Health Holland research grants for consortium projects with SkylineDx<br><br>1 author: rec'd institutional research grants from SkylineDx	*Methodology:* Retrospective cohort study.<br><br>Determine 5-year recurrence-free survival (RFS) of low- and high-risk CP-GEP in patients with negative SLN..	*Stated Objective:*<br>CP-GEP and recurrence risk.<br><br>"The primary aim of this retrospective study was to assess the performance of a clinicopathologic and gene expression (CP-GEP) model, a model originally developed to predict sentinel node metastasis, to identify patients with stage I–II melanoma at risk of disease relapse."<br><br>☐Prospective<br>☒Retrospective<br><br>*Study Population and Setting:*<br>Multi-site study<br>Swedish and Dutch patients without nodal metastases<br><br>*N:* 535<br><br>*Intervention:* CP-GEP, all patients had negative GEP CP-GEP assessment<br><br>*Outcome Measures:* RFS<br><br>*Follow-Up:*<br>More than 75% had >5-year f/u<br>medial FU not stated.<br>75.7% more than 5 yr FU available. 26% up to 10 yrs<br><br>*Notes:* EORTC model was also examined	*Results:*<br>Low and high-risk CP-GEP was able to discern 5-year RFS in a population of SLN-negative patients.<br><br>"In total, 535 patients (stage I–II) were included. CP-GEP stratification among these patients resulted in a 5-year RFS of 92.9% (95% confidence interval (CI): 86.4–96.4) in CP-GEP low-risk patients (n = 122) versus 80.7% (95%CI: 76.3–84.3) in CP-GEP high-risk patients (n = 413; hazard ratio 2.93 (95%CI: 1.41–6.09), p < 0.004). According to the EORTC nomogram, 25% of the patients were classified as having a 'low risk' of recurrence (96.8% 5-year RFS (95%CI 91.6–98.8), n = 130), 49% as 'intermediate risk' (88.4% 5-year RFS (95%CI 83.6–91.8), n = 261), and 26% as 'high risk' (61.1% 5-year RFS (95%CI 51.9–69.1), n = 137)."<br><br>Notes:	*Describe conclusions relative to question:*<br>Low and high-risk CP-GEP was able to discern 5-year RFS in a population of SLN-negative patients.<br><br>Supported by findings.<br><br>"in its present form, the CP-GEP model is not yet suitable for clinical practice as the current false positive rate is too high to select patients for adjuvant therapy and would result in overtreatment."<br><br>*Critiques of Methodology:*<br><br>No f/u to account for false negative results.<br><br>Two institutions<br>Limited dataset

	Summary of Evidence Table<br>Question 1					
Level of Evidence*^<br><br>Choose one:	Study Citation<br>Author(s), Year, Title, Journal, Volume, Page #s	Funding Source	Study Methodology	Objective & Methods	Results	Conclusions
☐EL 1; RCT<br>☐EL 1; MRCT<br>Strong<br><br>☐EL 2; MNRCT<br>☐EL 2; NMA<br>☐EL 2; NRCT<br>☒EL 2; PCS<br>☐EL 2; RCCS<br>☐EL 2; NCCS<br>☐EL 2; CSS<br>☐EL 2; ES<br>☐EL 2; OLES<br>☐EL 2; PHAS<br>Intermediate<br><br>☐EL 3; DS<br>☐EL 3; ECON<br>☐EL 3; CCS<br>☐EL 3; SCR<br>☐EL 3; PRECLIN<br>☐EL 3; BR<br>Weak<br><br>☐EL 4; NE<br>☐EL 4: O<br>No	Citation: Stassen, RC, Mulder, EAP et al Clinical evaluation of clinicopathologic and gene expression profile (CP-GEP) in patients with melanoma eligible for sentinel lymph node biopsy. A multicenter prospective Dutch study. *European Journal of Surgical Oncology* October 25, 2023<br><br>*Country:* Netherlands (multicenter)	*Source:*<br><br>This study was partially funded by SkylineDx.	*Methodology:* Prospective multicenter study<br><br>Prospective multicenter CP-GEP performed onl pt T1b-T4 and all had SLNB<br><br>Using the result of the SLNB pathology as the gold standard, the performance of the model was calculated. Model performance was assessed by calculating sensitivity, specificity, negative predictive value<br><br>(NPV), positive predictive value (PPV), SLNB	*Stated Objective:*<br><br>to evaluate the clinical use and implementation of the CP-GEP model in a prospective multicenter study in the Netherlands.<br><br>Assess the Merlin assay (CP-GEP) in its effectiveness for allowing appropriate reduction of SLN procedure in T1b-T4 melanomas<br><br>☒Prospective<br>☐Retrospective<br><br>*Study Population and Setting:*<br><br>Mutli-institutional ≥18 years, with newly diagnosed primary cutaneous melanoma, eligible for SLNB (pT1b– pT4), Stage I-II<br><br>*N:*<br>260<br>N=222 (included for predictive utility analysis) since 38 patients did not undergo SLN biopsy<br><br>*Intervention:* CP-GEP Merlin Assay<br><br>*Outcome Measures:*	*Results:*<br><br>Negative predictive value of assay: 96.7%<br>Positive predictive value: 23.7%<br><br>Potential reduction of SLN biopsy in T1-T3 melanomas with use of assay: 42.2%<br><br>NPV in T2 lesions: 100%<br><br>CP-GEP model demonstrated an overall negative predictive value of 96.7% and positive predictive value of 23.7%, with a potential SLNB reduction rate of 42.2% in patients with T1-T3 melanoma.<br>The median IC-to-report time was 16 days (IQR 14–20 days), The median IC-to-report time was 16 days (IQR 14–20 days).<br><br>One-hundred of 260 patients (38.5%) had a low risk<br>for a positive SN. Among all patients, the model yielded a sensitivity of<br>91.4% (95% CI: 76.9–98.2), a specificity of	*Describe conclusions relative to question:*<br><br>The model may support clinical decision-making to identify patients who can forgo SLNB in clinical practice.<br><br>Merlin CP-GEP assay may have utility in clinical decision making for which patients can forego SLN biopsy procedure<br><br>*Critiques of Methodology:*<br><br>Small number of SLN positive patients in T1b (N=3) and T2 lesions (N=15)<br><br>Larger number of patients needed for analysis with so many smaller groups t1b-T4,

			reduction rate and corre-sponding 95% confidence intervals.	NPV, PPV<br>SLNB reduction rate<br><br>Time order to report= order to revision + revision to shipment + shipment to report.<br><br>*Follow-Up:*<br>Na<br><br>*Notes:* N should include 5 patients that CP-GEP was invalid	45.8% (95% CI: 38.6–53.2),<br>a NPV of 96.7% (95% CI: 90.6–99.3) and a PPV of 23.7% (95% CI: 16.8–31.8). The SLNB reduction rate was 39.2% (95% CI: 32.7–45.9).<br><br>of CP-GEP low risk cases where found, the model yielded a sensitivity of 84.2% (95% CI: 60.4–96.6), a specificity of 57.3% (95% CI: 48.8–65.6), a NPV of 96.5% (95% CI: 90.0–99.2) and PPV of 20.7% (95% CI: 12.4–31.5). In this group, a SLNB reduction rate of 52.5% (95% CI: 44.5–60.5) was found.<br><br>Notes:	high low risk and whether +/- SLN.<br><br>Only 112 T2 pt<br><br>All T4 classified as high risk, and majority T3 so unclear benefit of GEP offer over histopathology.<br><br>Utility<br>Need follow up for in-nodal basin recurrence.

	Summary of Evidence Table<br>Question 1, 2, 3					
Level of Evidence*^<br><br>Choose one:	Study Citation<br>Author(s), Year, Title, Journal, Volume, Page #s	Funding Source	Study Methodology	Objective & Methods	Results	Conclusions
Strong<br>☐EL 1; RCT<br>☐EL 1; MRCT<br><br>Intermediate<br>☐EL 2; MNRCT<br>☐EL 2; NMA<br>☐EL 2; NRCT<br>☐EL 2; PCS<br>☐EL 2; RCCS<br>☐EL 2; NCCS<br>☐EL 2; CSS<br>☐EL 2; ES<br>☐EL 2; OLES<br>☐EL 2; PHAS<br><br>Weak<br>☐EL 3; DS<br>☐EL 3; ECON<br>☐EL 3; CCS<br>☐EL 3; SCR<br>☐EL 3; PRECLIN<br>☐EL 3; BR<br><br>No Evidence<br>☒EL 4; NE<br>☐EL 4: O	Svoboda RM, Glazer AM, Farberg AS, Rigel DS. Factors Affecting Dermatologists' Use of a 31-Gene Expression Profiling Test as an Adjunct for Predicting Metastatic Risk in Cutaneous Melanoma. J. Drugs Dermatol. 2018;17(5):544-547. (35)<br><br>*Country:*<br>USA	*Source:*<br><br>This study was funded in part by a grant from Castle Biosciences Inc.<br><br>1 author: consultant to Castle Bioscience<br><br>1 author: served on advisory board for Castle Bioscience<br><br>2 authors: participated in research fellowship that was partially funded by Castle Bioscience	*Methodology:*<br><br>Dermatologists answered questionnaire with four clinical vignettes to determine the impact of Breslow thickness, ulceration and SLNBx status decision to order GEP test.<br><br>Survey-based study performed at a conference.	*Stated Objective:*<br>Clinical factors that impact dermatologists' decisions to utilize 31-GEP.<br><br>☒Prospective<br>☐Retrospective<br><br>*Study Population and Setting*:<br>Dermatology questionnaire at a national conference.<br><br>Panel of clinicians.<br>Survey<br><br>*N:* 181/187<br><br>*Intervention:*<br>Questionnaire with four clinical vignettes.<br><br>Opinion regarding management<br><br>*Outcome Measures:*<br>Percentage of respondents who would order 31-GEP in clinical scenarios.<br><br>Recommended treatment pathway.<br><br>*Follow-Up*: N/A<br><br>*Notes:*	*Results:*<br>A majority of patients would recommend GEP test."<br><br>Breslow thickness ≥ 0.5 mm, majority dermatologists would order GEP. Ulceration was associated with a statistically significant increase to recommend for all but the thickest ≥ 2.1 mm. For thin tumor (0.26 mm) ulceration significantly changed from 22% to 67%, p <0.001). A negative SLNBx only associated with statistically significant increase in the percentage for the thinnest tumors (22% to 34%, p=0.033).<br><br>Impact of GEP test result on T1b 0.76-1.0 mm melanoma:<br>Class 1 result -91% respondents reported less likely to recommend SLNBx.<br>Class 2 - 81% would make more likely to recommend SLNBx.<br><br>*Notes:*	*Describe conclusions relative to question:*<br>Not relevant to current clinical practice.<br><br>Ulceration most important factor deciding to order 31-GEP.<br><br>*Critiques of Methodology:*<br>Authors do not state whether the survey was taken at a session paid for by Castle Bioscience.<br><br>Authors have conflict of interest – was the survey voluntary<br><br>-Survey of dermatologists' ordering pattern does not evaluate the validity of the test.<br>- Selection bias in those willing to take the survey<br>-was survey voluntary or was

						there an incentive.

Level of Evidence*^ Choose one:	Summary of Evidence Table Question 1, 2, 3<br>Study Citation Author(s), Year, Title, Journal, Volume, Page #s	Funding Source	Study Methodology	Objective & Methods	Results	Conclusions
Strong<br>☐EL 1; RCT<br>☐EL 1; MRCT<br><br>Intermediate<br>☐EL 2; MNRCT<br>☐EL 2; NMA<br>☐EL 2; NRCT<br>☐EL 2; PCS<br>☐EL 2; RCCS<br>☐EL 2; NCCS<br>☐EL 2; CSS<br>☐EL 2; ES<br>☐EL 2; OLES<br>☐EL 2; PHAS<br><br>Weak<br>☐EL 3; DS<br>☐EL 3; ECON<br>☐EL 3; CCS<br>☐EL 3; SCR<br>☐EL 3; PRECLIN<br>☐EL 3; BR<br><br>No Evidence<br>☒EL 4; NE<br>☐EL 4: O	Swetter SM, Tsao H., Bichakjian CK, Curiel-Lewandrowski C, Ender DE, Gershewald JE, et al. Guidelines of Care for the Management of Primary Cutaneous Melanoma. *J. Am. Acad. Dermatol.* 2019 Jan 1; 80(1):208-250. (117)<br><br>*Country:* USA	*Source:* None<br><br>1 author: served as an advisory board member to Castle Biosciences, receiving fees.	*Methodology*: Practice guideline development.<br><br>AAD guidelines for the management of primary cutaneous melanoma from a group of dermatologists, patient advocate, at least one surgeon<br><br>2018	*Stated Objective:*<br><br>*☐Prospective*<br>*☐Retrospective*<br><br>*Study Population and Setting:*<br><br>*N:*<br><br>*Intervention:*<br><br>*Outcome Measures:*<br><br>*Follow-Up:*<br><br>*Notes:*	*Results:*<br><br>GEP testing can stratify patients into risk categories with some degree of precision; however the data to date consists of heterogenous groups of high risk event enriched groups that may not represent the spectrum of melanoma patient population and the clinical applicability of the tests is uncertain based on the lack of surgical or therapeutic RCTS to examine outcomes with GEP testing.<br><br>Page 230 of the article addresses GEP and cautions against routine use for clinical decision-making pending appropriate clinical trials evaluating clinical usefulness of tests and whether currently available tests add to AJCC8 staging.<br><br>*Notes:*	*Describe conclusions relative to question:*<br><br>The working group discourages baseline GEP testing for prognostication and for management decisions.<br><br>AAD opinion on GEP provided on p 230.<br><br>*Critiques of Methodology:*

	Summary of Evidence Table<br>Question 1, 2					
Level of Evidence*^<br><br>Choose one:	Study Citation<br>Author(s), Year, Title, Journal, Volume, Page #s	Funding Source	Study Methodology	Objective & Methods	Results	Conclusions
Strong<br>☐EL 1; RCT<br>☐EL 1; MRCT<br><br>Intermediate<br>☐EL 2; MNRCT<br>☐EL 2; NMA<br>☐EL 2; NRCT<br>☒EL 2; PCS<br>☐EL 2; RCCS<br>☐EL 2; NCCS<br>☐EL 2; CSS<br>☐EL 2; ES<br>☐EL 2; OLES<br>☐EL 2; PHAS<br><br>Weak<br>☐EL 3; DS<br>☐EL 3; ECON<br>☐EL 3; CCS<br>☐EL 3; SCR<br>☐EL 3; PRECLIN<br>☐EL 3; BR<br><br>No Evidence<br>☐EL 4; NE<br>☐EL 4: O	Thorpe RB, Covington KR, Caruso HG, et al. Development and Validation of a Nomogram Incorporating Gene Expression Profiling and Clinical Factors for Accurate Prediction of Metastasi in Patients with Cutaneous Melanoma following Mohs Micrographic Surgery. J. Am. Acad. Dermatol. 2022;86(4):846-853. (96)<br><br>*Country*: USA 9 Mohs private practices	*Source:*<br>1. Statistician provided by Castle Biosciences<br>2. IRB funded by Skin Cancer Trust for independent Research and Education, a 501c3 managed by Drs. Zitelli and Brodland<br><br>4 authors: employees of Castle Biosciences<br><br>4 authors: stockholders at Castle Biosciences	*Methodology:*<br>Prospective testing with 31-GEP of patients with melanoma undergoing melanoma surgery at Mohs surgery center.<br><br>Melanoma patients with complete clinical and pathologic information including 31-GEP enrolled from the nine centers<br><br>Years of enrollment not stated.<br><br>All pts had Mohs with six mm initial margins with further margins until clear by MART1 IHC.	*Stated Objective:*<br>To develop a nomogram for predicting metastasis using 31-GEP and T stage.<br><br>To define a nomogram for practical clinical use using 31-GEP.<br><br>☒Prospective<br>☐Retrospective<br><br>*Study Population and Setting:*<br>Patients undergoing melanoma surgery at nine Mohs surgery centers.<br><br>Nine Mohs Private Derm Practices<br><br>*N:* 1124 enrolled – data presented is from 684 patients followed for min 1 yr or a "metastatic event" as those used for nomogram development (p847).<br><br>*Intervention:* Mohs + 31-GEP<br><br>*Outcome Measures:*<br>Nomogram development for predictng metastasis using<br><br>Risk of metastasis using a nomogram<br><br>*Follow-Up:*	*Results:*<br><br>31-GEP test and T stage offers simplest nomogram with lowest Bayesian information score—validated in a separate cohort of 905 patients.<br><br>Class 2B patients do worse (53/684 = 7.7% of the cohort<br>Don't see where the nomogram considering Breslow depth and 31-GEP adds value over either alone (see Table II) except maybe T2-T3a and shifts %s in clinically unmeaningful way – wouldn't change f/u or rx recommendations.<br><br>*Notes:*<br><br>Mohs for invasive melanoma is unsupported by RCT evidence.<br>Graphs present five year survival without numbers at risk data & P value doesn't state what the comparison is (class IA,1B,2A all appear to overlap on the RFS and DMFS curves)<br>Demographics in supplemental table mean BT 0.5 mm (0.1-13.0 mm)	*Describe conclusions relative to question:*<br><br>31-GEP test and T stage can provide prognostic information for metastasis.<br><br>Question biopsy as non-invasive (need to do it to get T category which they refer to as T stage).<br><br>*Critiques of Methodology:*<br>1. SLN biopsy not done for many patients and not included in the nomogram<br>2. Limited to patients undergoing surgery at Mohs surgery centers which may reflect a selection bias (very few T2B

				At least one year f/u or metastatic event; median f/u 3.2 years<br><br>Median 3.2 years<br><br>*Notes:*	and correlate with 31-GEP class for entire 1124 pt cohort not the 684 used to develop nomogram.	and higher lesions)<br>3. Relatively short f/u particularly for earlier T stage lesions<br><br>Study design has flaws:<br>Patient selection<br>No SLNB in 654 of the 684 pts<br>No prospective validation cohort for nomogram – used an “archival cohort of 901 stage I-III patients w/ median 6.1 yrs f/u from 22 centers (no ref) to “validate” which is different from this study population.

	Summary of Evidence Table<br>Question 1					
Level of Evidence*^<br><br>Choose one:	Study Citation<br>Author(s), Year, Title, Journal, Volume, Page #s	Funding Source	Study Methodology	Objective & Methods	Results	Conclusions
Strong<br>☐EL 1; RCT<br>☐EL 1; MRCT<br><br>Intermediate<br>☐EL 2; MNRCT<br>☐EL 2; NMA<br>☐EL 2; NRCT<br>☐EL 2; PCS<br>☒EL 2; RCCS<br>☐EL 2; NCCS<br>☐EL 2; CSS<br>☐EL 2; ES<br>☐EL 2; OLES<br>☐EL 2; PHAS<br><br>Weak<br>☐EL 3; DS<br>☐EL 3; ECON<br>☐EL 3; CCS<br>☐EL 3; SCR<br>☐EL 3; PRECLIN<br>☐EL 3; BR<br><br>No Evidence<br>☐EL 4; NE<br>☐EL 4: O	Vetto JT, Hsueh EC, Gastman BR, et al. Guidance of Sentinel Lymph Node Biopsy Decisions in Patients with T1-T2 Melanoma Using Gene Expression Profiling. Future Oncol. 2019;15(11):1207-1217.<br>(51)<br><br>*Country:* United States	*Source:*<br><br>Castle Biosciences funded study. They drafted the study design and oversaw the data collection, management and all analyses. The contributed to data interpretation; preparation, review and approval of the manuscript.<br><br>2 authors: employees of Castle Bioscience<br><br>2 authors: are options holders at Castle Bioscience<br><br>Acknowledgement of 4 Castle Bioscience employees for contributions to the study<br><br>1 author: paid consultant to Castle Bioscience<br><br>1 author: received an honorarium from Castle Bioscience<br><br>5 authors: on speakers bureau for Castle Bioscience<br>3 authors: rec'd travel/meeting funding	*Methodology:*<br><br>Retrospective analysis of a cohort of patients.<br><br>Cohort 1 (n=584 pts enrolled in 1 of 2 prospective, registry studies and a prospective clinical utility study). Cohort 2 (n=837 cases) from retrospective pts/samples from 2014 – 2017. "To determine the clinical outcome impact of using this approach to determine whether to do an SLNB or not, long-term clinical outcome data were assessed from 690 patients who had more	*Stated Objective:* To determine if a GEP can help identify patients with a low risk (<5%) of a positive node.<br><br>Rules-based and regression analyses to determine significant predictors of SLN positivity and identification of optimal cut-offs to define a population in which gene expression could better predict SLNB results.<br><br>☒Prospective<br>☒Retrospective<br><br>Prospective and retrospective datasets<br><br>*Study Population and Setting:* Multiple academic centers, patients with melanoma and known outcome.<br><br>*N:* 946 to pre-calculate a target population by clinicodemographic analysis. Then 2 prospective cohorts collected and analyzed retrospectively, 584 pts, 837 pts.<br><br>Very confusing.<br><br>*Intervention:* Bioinformatic analysis	*Results:*<br><br>*Notes:*	*Describe conclusions relative to question*:<br><br>The aim of this study was to evaluate whether the GEP test could identify patients with low risk for SLN positivity in the T1–T2 melanoma population who currently would be considered for SLNB based on guidelines.<br><br>*Critiques of Methodology:*<br><br>The study analyzed T1a patients, an astonishing 34% of whom had a SLNB. It also does not seem as though they stratified for T1a patients vs T1b or T2 to compare who was GEP Class IA or Class 2b as such, it is unclear whether it was the GEP classification or T stage that was independently

		unrelated to this study from Castle Bioscience<br><br>13 authors: rec'd funding for sample and/or clinical data acquisition and processing for this study<br><br>2 authors: rec'd travel funding to present data related to this study at a meeting and/or other purposes	than 5 years of follow-up or a documented recurrence of melanoma [24,25,27,32].<br><br>These patients represent a subset of the 946 patient retrospective cohort described above."	Outcome Measures: Sentinel Node status<br><br>Follow-Up: 5 years<br><br>____This is a very curated dataset_<br><br>See Table 1.<br><br>____________________<br>*Notes:*		impactful in predicting SLN status.<br><br>Not sure that the indications to perform a SLNB for T1a melanomas are widely used in other institutions.<br><br>Methodology: Cohorts are not clearly defined.

Summary of Evidence Table
Question 1

Level of Evidence*^<br>Choose one:	Study Citation<br>Author(s), Year, Title, Journal, Volume, Page #s	Funding Source	Study Methodology	Objective & Methods	Results	Conclusions
Strong<br>☐EL 1; RCT<br>☐EL 1; MRCT<br><br>Intermediate<br>☐EL 2; MNRCT<br>☐EL 2; NMA<br>☐EL 2; NRCT<br>☐EL 2; PCS<br>☐EL 2; RCCS<br>☐EL 2; NCCS<br>☐EL 2; CSS<br>☐EL 2; ES<br>☐EL 2; OLES<br>☒EL 2; PHAS<br><br>Weak<br>☐EL 3; DS<br>☐EL 3; ECON<br>☐EL 3; CCS<br>☐EL 3; SCR<br>☐EL 3; PRECLIN<br>☐EL 3; BR<br><br>No Evidence<br>☐EL 4; NE<br>☐EL 4: O	Whitman ED, Koshenkov VP, Gastman BR, et al. Integrating 31-Gene Expression Profiling With Clinicopathologic Features to Optimize Cutaneous Melanoma Sentinel Lymph Node Metastasis Prediction. *JCO Precis Oncol.* 2021;5:1466-1479. (78)<br><br>*Country:* USA	*Source*: Supported by Castle Biosciences, Inc.<br><br>7 authors: employees of Castle Biosciences<br><br>6 author: consulting or advisory role with Castle Bioscience<br><br>8 author: speakers bureau Castle Bioscience<br><br>4 author: research funding Castle Bioscience<br><br>9 author: stock and other ownership interests Castle Biosciences<br><br>7 author: travel accommodations, expenses Castle Bioscience<br><br>3 author: patent application pending or related for Castle Bioscience	*Methodology:* Development of neural network algorithm (AI-based).<br><br>Analysis of existing dataset.	*Stated Objective:* Prediction of SLN status.<br><br>Use AI to improve metastases prediction.<br><br>☐Prospective<br>☒Retrospective<br><br>*Study Population and Setting:*<br>Multi-institutional<br><br>Existing dataset – details not provided in this paper.<br><br>*N:* 1398 development cohort, 1674 validation cohort<br><br>Patient cohort (n = 1,398) and validated using an independent cohort (n = 1,674).<br><br>*Intervention*: GEP<br><br>Cohort analysis using AI to improve predictive value.<br><br>*Outcome Measures:* <5% or >10% risk of SLN positivity.<br><br>Likelihood of positive nodes on SLN bx<br><br>*Follow-Up:* n/a<br>Not given	*Results:*<br>Neural network algorithm helps predict SLN positivity.<br><br>Compared with other covariates in the i31-GEP, the continuous 31-GEP score had the largest likelihood ratio (G2 = 91.3, P , .001) for predicting SLN positivity. The i31-GEP demonstrated high concordance between predicted and observed SLN positivity rates (linear regression slope = 0.999). The i31-GEP increased the percentage of patients with T1-T4 tumors predicted to have 5% SLN-positive likelihood from 8.5% to 27.7% with a negative predictive value of 98%. Importantly, for patients with T1 tumors originally classified with a likelihood of SLN positivity of 5%-10%, the i31-GEP reclassified 63% of cases as having 5% or . 10% likelihood of positive SLN, for a more precise, personalized, and clinically actionable SLN-positive likelihood estimate.<br><br>*Notes:*	*Describe conclusions relative to question:*<br><br>GEP results can help inform an algorithm which predicts SLN positivity and increases accuracy of prediction.<br><br>AI improved model accuracy.<br><br>*Critiques of Methodology:*<br>Impact on decision making for a small number of patients; unclear impact since the results are still “binned” into categories.<br><br>20% with “clinically known SLN status”? 25% of validation has SLN status determined clinically.<br>Not a clinical trial.

		1 author: leadership Castle Bioscience<br><br>1 author: honoraria Castle Bioscience<br><br>1 author: expert testimony Castle Bioscience		*Notes:*	Results from development cohort were previously reported (Vetto, Future Oncol 2019).	

	Summary of Evidence Table<br>Question 1					
Level of Evidence*^<br><br>Choose one:	Study Citation<br>Author(s), Year, Title, Journal, Volume, Page #s	Funding Source	Study Methodology	Objective & Methods	Results	Conclusions
**Strong**<br>☐EL 1; RCT<br>☐EL 1; MRCT<br><br>**Intermediate**<br>☐EL 2; MNRCT<br>☐EL 2; NMA<br>☐EL 2; NRCT<br>☐EL 2; PCS<br>☐EL 2; RCCS<br>☐EL 2; NCCS<br>☐EL 2; CSS<br>☐EL 2; ES<br>☐EL 2; OLES<br>☐EL 2; PHAS<br><br>**Weak**<br>☐EL 3; DS<br>☐EL 3; ECON<br>☒EL 3; CCS<br>☐EL 3; SCR<br>☐EL 3; PRECLIN<br>☐EL 3; BR<br><br>**No Evidence**<br>☐EL 4; NE<br>☐EL 4: O	Yamamoto M, Sickle-Santanello B, Beard T, et al. The 31-Gene Expression Profile Test Informs Sentinel Lymph Node Biopsy Decisions In Patients With Cutaneous Melanoma: Results Of A Prospective, Multicenter Study. *Curr Med Res Opin.* 2023;39(3):417-423.<br><br>Country: USA	Source:<br>This study was funded by Castle Biosciences.<br><br>2 authors: Castle Biosciences employees<br><br>2 authors: speakers bureau Castle Biosciences<br><br>1 author: advisor for Castle Biosciences<br><br>2 authors: stock options holders at Castle Biosciences	Methodology:<br><br>Prospective cohort survey study<br><br>Case series, not even sure it is a consecutive case series<br><br>Prospective assessment of clinical decision making based upon GEP results<br><br>Clinicians sent 31-GEP at clinic visit 1, discussed results to perform SLNB at visit 2. Decision at Visit 3.<br><br>The association between tumor, clinical, and pathological features with SLNB performance was studied by using stepwise selection on a logistic regression	Stated Objective:<br>Quantify SLNB reduction by clinicians using 31-GEP<br><br>Determine the impact of 31-GEP testing on decision for SLNB.<br><br>☒Prospective<br>☐Retrospective<br><br>Study Population and Setting:<br>Clinicians seeing T1-T2 eligible for SLNB.<br><br>Clinical decision for SLNB in 22 institutions<br><br>Multi-center 22 institutions including academic and community T1,T2 melanomas<br><br>N: 191 decisions<br># of clinicians not reported<br>N: 193<br><br>Intervention: 31-GEP results<br>Was 31 GEP test influential in decision to perform or not perform SLNB<br><br>Outcome Measures: Effect of 31-GEP results on conductance of SLNB.	Results:<br><br>100/191 patients decision to forego SLNB was influenced by Decision dx test; 70% of these patients did not undergo SLNB; in 30% of patients in which SLNB performed in any case (83% driven by patient preference) 0% of SLNs were positive.<br>In 63/191 patients, Decision DX test influenced decision for SLNB; Overall, influence of DecisionDX on SLNB was 85.3%.<br><br>SLNB with GEP guidance was reduced compared to historical control of 80%<br><br>85.3% decisions relating to SLNB influenced by 31-GEP.<br><br>Clinicians performed 29.4% fewer biopsies with FNR for class 1A 3.0% (2/67)<br><br>52.4% (100/191) decisions influenced to	Describe conclusions relative to question:<br><br>85% of decisions related to SLNB were influenced by GEP and SLNB are reduced by using GEP.<br><br>Clinicians use 31-GEP to guide SLNB. Risk appropriate decrease in SLNB<br><br>A significant proportion of decisions for performance of SLNB for T1/T2 melanomas are influenced by Decision Dx results<br><br>Critiques of Methodology:<br><br>Unconventional design because survey mixed with outcomes of two tests 31-GEP and SLNB.

			model incorporating the 31-GEP result, Breslow thickness, ulceration, mitotic rate, LVI, transected base, presence of regression, presence of TILs, microsatellites, patient preference, patient age, and patient comorbidities<br><br>SLNB reduction rates were analyzed based on a previous algorithm indicating that patients with a 31-GEP Class 1A result who are either 65 years old (primary analysis) or 55 years old (secondary analysis) with T1- T2 tumors have a < 5% risk of SLN positivity<br><br>172 patients needed	SLNB reduction rate compared to historical control<br><br>Surgeon report of utility of 31 GEP in decision to perform or not perform SNLB<br><br>What proportion of SLNB eligible patients decision for SLNB was influenced by DecisionDx test<br><br>Follow-Up: n/a<br><br>Notes: The title of the journal is informative: Medical Research and Opinion.	forgo SLNB with 70/100 not performed<br><br>Clinicians 89.1% surg onc, 7.8% dermatologists, 3.1% med onc.<br><br>32.6% (63/191) influenced to perform with 92/1% performed.<br><br>SLNB performance rate 59.1% lower that baseline 78%<br><br>SLNB positivity class 1A 3.0%, comparison to Class1B/2Aperformance rate 80.6%, positivity rate 13.8%; and class 2B SLNB performance 94.7% and positivity 22.2%<br><br>Pt > 55 yo performance class<br>IA SLNB 44.6 %, + 2.2%<br>1B/2A 77.4%, 16.7%<br>2B 100%, 26.7%<br><br>Pt > 65 yo performance class; SLNB rate, positive<br>IA SLNB 48.0 %, + 0%<br>1B/2A 71.4%, +26.7%<br>2B 100%, +33.3%<br><br>52% of clinical decisions were influenced by the results of the 31-GEP test<br><br>Notes:<br>Lowest risk group Class IA doesn't make sense influenced 88.9% (16/18) to perform SLNB. What was time to treatment	Tumor location not variable<br><br>Do not explain survey of clinicians in 31-GEP and other factors influencing decision.<br><br>SLNB reduction rates analyzed using algorithm based on prior 31-GEP study.<br><br>Grouped class IB/2A.<br><br>No patient outcomes are tied to decision making. The clinician is what is being studied here and unclear how many clinicians were included in study<br><br>Bias in case selection – they were all patients who had the test done<br>No comparison to other methods of predicting SLN positivity<br><br>There is no outcome data—specifically no data on which patients who did not

					considering that Castel ordered at clinic visit 1.<br><br>Factors such as LVI, microsatellites, ulceration had no impact on SLNB decision.<br><br>Sensitivity analysis oddly reported with rate of SLNB.<br><br>Not clear what an appropriate historical control is for this cohort.<br><br>The question here appears to be when the test was ordered and resulted prior to therapeutic operation for melanoma did the test results influence the operative plan (SLNB planned or omitted)<br><br>Notes:	undergo SLNB developed regional nodal disease—does not therefore really provide meaningful data on the accuracy of this assay

	Summary of Evidence Table<br>Question 1					
Level of Evidence*^<br><br>Choose one:	Study Citation<br>Author(s), Year, Title, Journal, Volume, Page #s	Funding Source	Study Methodology	Objective & Methods	Results	Conclusions
Strong<br>☐EL 1; RCT<br>☐EL 1; MRCT<br><br>Intermediate<br>☐EL 2; MNRCT<br>☐EL 2; NMA<br>☐EL 2; NRCT<br>☐EL 2; PCS<br>☒EL 2; RCCS<br>☐EL 2; NCCS<br>☐EL 2; CSS<br>☐EL 2; ES<br>☐EL 2; OLES<br>☐EL 2; PHAS<br><br>Weak<br>☐EL 3; DS<br>☐EL 3; ECON<br>☐EL 3; CCS<br>☐EL 3; SCR<br>☐EL 3; PRECLIN<br>☐EL 3; BR<br><br>No Evidence<br>☐EL 4; NE<br>☐EL 4: O	Yousaf A, Tjien-Fooh FJ, Rentroia-Pacheco B, et al. Validation of CP-GEP (Merlin Assay) for Predicting Sentinel Lymph Node Metastasis in Primary Cutaneous Melanoma Patients: A U.S. Cohort Study. Intern J.Dermatol. 2021;60(7):851-856. (79)<br><br>*Country:* USA	*Source:* National Cancer Institute, CA215105<br><br>3 authors: employees SkylineDx<br><br>Mayo Clinic and 1 author have a financial conflict of interest in the Merlin Assay.<br><br>2 authors: rec'd research funding SkylineDx<br><br>3 authors: equity stakes in SkylineDx	*Methodology:* Validation of CP-GEP model<br><br>Validation cohort	*Stated Objective:* Prediction of SLNB status,<br><br>Validation cohort SLN outcomes<br><br>☐Prospective<br>☒Retrospective<br><br>*Study Population and Setting:* Mayo and WVU patients<br><br>*N*: 208<br><br>*Intervention:* CP-GEP<br><br>*Outcome Measures:* SLN positivity<br><br>The main performance measures were SLNB reduction rate (RR) and negative predictive value (NPV).<br><br>*Follow-Up:* n/a<br><br>Mayo Clinic in Minnesota, Arizona, or Florida between 2004 and 2019 or the West Virginia University between 2007 and 2014.<br><br>*Notes:*	*Results:* NPV 93.8%<br><br>SLNB positivity rate for the entire cohort was 21%. Most patients had a T1b (34%) or T2a (31%) melanoma. In the T1-T2 group (153 patients), CP-GEP achieved an SLNB RR of 41.8% (95% CI: 33.9-50.1) at an NPV of 93.8% (95% CI: 84.8-98.3). Subgroup analysis showed similar performance in T1-T2 patients ≥65 years of age (51 patients; SLNB positivity rate, 9.8%): SLNB RR of 43.1% (95% CI: 29.3-57.8) at an NPV of 95.5% (95% CI: 77.2-99.9).<br><br>*Notes:*	*Describe conclusions relative to question*:<br><br>CP-GEP could help predict SLN status and reduce need for SLNB.<br><br>Conclusions are supported.<br><br>*Critiques of Methodology:* No f/u to account for false negative results.<br><br>Relatively small validation set.<br><br>Retrospective<br><br>Two institutions

Summary of Evidence Table<br>Question 1, 2						
Level of Evidence*^<br><br>Choose one:	Study Citation<br>Author(s), Year, Title, Journal, Volume, Page #s	Funding Source	Study Methodology	Objective & Methods	Results	Conclusions
☐EL 1; RCT<br>☐EL 1; MRCT<br>(Strong)<br><br>☐EL 2; MNRCT<br>☐EL 2; NMA<br>☐EL 2; NRCT<br>☐EL 2; PCS<br>☐EL 2; RCCS<br>☐EL 2; NCCS<br>☐EL 2; CSS<br>☐EL 2; ES<br>☐EL 2; OLES<br>☐EL 2; PHAS<br>(Intermediate)<br><br>☐EL 3; DS<br>☐EL 3; ECON<br>☐EL 3; CCS<br>☐EL 3; SCR<br>☐EL 3; PRECLIN<br>☐EL 3; BR<br>(Weak)<br><br>☒EL 4; NE<br>☐EL 4: O<br>(No Evidence)	Zakria D, Brownstone N, Berman B, Ceilley R, Goldenberg G, Lebwohl M, Litchman G, & Siegel D. Incorporating Prognostic Gene Expression Profile Assays into the Management of Cutaneous Melanoma: An Expert Consensus Panel Report. *SKIN The Journal of Cutaneous Medicine.* 2023; 7(1): 556–569.<br><br>*Country:* USA	*Source:*<br><br>This study was funded in part by an unrestricted educational grant from Castle Biosciences.<br><br>2 author: consultant to Castle Biosciences<br><br>1 author: served on advisory boards for Castle Biosciences	*Methodology:* Review of literature; consensus study.<br><br>Literature review and consensus panel.	*Stated Objective:*<br><br>☐Prospective<br>☒Retrospective<br><br>*Study Population and Setting:*<br><br>*N:* 785 article that met search criteria; 22 validating 31-GEP; 2 11-GEP and 7 8 GEP<br><br>*Intervention:*<br><br>*Outcome Measures:*<br><br>*Follow-Up:*<br><br>*Notes*: 22 articles used to inform the panel and 11 consensus recommendations	*Results:*<br>Review of current literature with level of evidence assessments.<br><br>SORT level A recommendations were made for<br>--GEP testing can identify a high-risk subset for recurrence, distant metastasis, or death of traditionally assessed low-risk patients (e.g., SLN negative or T1a/b) (SORT Level=A)<br>--GEP testing provides clinically useful information that augments risk-aligned management decisions to both rule-in or rule-out the need for SLNB and subsequent management plans (SORT Level=A)<br><br>SORT level B recommendations were made for<br>--Integrating GEP results can increase the precision and confidence of melanoma management decisions (e.g., follow up regimens, decision for SLNB, referral to other specialties, and need for imaging) (SORT Level=B)<br><br>*Notes:*	*Describe conclusions relative to question:*<br><br>GEP tests appear to offer valuable prognostic information and assistance with clinical management of patients with cutaneous melanoma over and above AJCC staging and NCCN guidelines; there appears to be more published data supporting the clinical utility of 31- GEP test.<br><br>General support for use of GEP in practice.<br><br>*Critiques of Methodology:*<br>Should be noted that study funded by Castle Biosciences grant Conclusions do not appear to be entirely supported by data of published literature,

					SORT is Strength of Recommendation Taxonomy (A, B, or C)	particularly for T1 lesions<br><br>This is a consensus panel (recommendations made as the National Society for Cutaneous Medicine)

Question 2

Does GEP testing improve current risk-stratification of adult patients with AJCC pT1a-pT4b primary cutaneous melanoma sufficiently to recommend its utilization to guide decision-making for surveillance imaging and follow-up?

	Summary of Evidence Table<br>Question 2					
Level of Evidence*^<br><br>Choose one:	Study Citation<br>Author(s), Year, Title, Journal, Volume, Page #s	Funding Source	Study Methodology	Objective & Methods	Results	Conclusions
**Strong**<br>☐EL 1; RCT<br>☐EL 1; MRCT<br><br>**Intermediate**<br>☐EL 2; MNRCT<br>☐EL 2; NMA<br>☐EL 2; NRCT<br>☐EL 2; PCS<br>☒EL 2; RCCS<br>☐EL 2; NCCS<br>☐EL 2; CSS<br>☐EL 2; ES<br>☐EL 2; OLES<br>☐EL 2; PHAS<br><br>**Weak**<br>☐EL 3; DS<br>☐EL 3; ECON<br>☐EL 3; CCS<br>☐EL 3; SCR<br>☐EL 3; PRECLIN<br>☐EL 3; BR<br><br>**No Evidence**<br>☐EL 4; NE<br>☐EL 4: O	Amaral TM, Hoffman MC, Sinnberg T, et al. Clinical Validation of a Prognostic 11-Gene Expression Profiling Score in Prospectively Collected FFPE Tissue of Patients with AJCC v8 Stage II Cutaneous Melanoma. *Eur J Cancer.* 2020 Jan;125:38-45. Epub 2019 Dec 12. (111)<br><br>*Country:* Germany	*Source:*<br>None declared;<br><br>3 authors rec'd grants and/or personal fees from Neracare while conducting the study.<br><br>1 author rec'd grants from Neracare outside the study.<br><br>1 author is a Neracare employee since 2017.	*Methodology:*<br>Retrospective cohort study.<br><br>Stage II melanoma pts 2000-2016 with tissue for testing from German registry.	*Stated Objective:*<br>Assess the prognostic utility of 11-gene signature in a dichotomized manner.<br><br>☐Prospective<br>☒Retrospective<br><br>*Study Population and Setting:*<br>Stage II patients with melanoma between 2000 and 2016;German registry<br><br>*N:* 246<br>24 of 302 with tissue from 1755 stage II melanoma pts in registry.<br><br>*Intervention:* 11-GEP<br><br>*Outcome Measures:*<br>Melanoma specific survival<br>Distant metastasis free survival<br>Recurrence free survival<br><br>*Follow-Up:* 41 months<br><br>*Notes*:<br>Analyzed MelaGenix score as dichotomized and continuous variable but modeling with dichotomized variable as low vs high risk	*Results:* 11-GEP signature was significantly associated with MSS, DMFS, and RFS and independently associated with MSS when accounting for tumor thickness and age.<br><br>11-GEP predictive of 5 and 10 year MSS, p=0.18<br><br>*Notes:*	*Describe conclusions relative to question:*<br>11-GEP signature can independently predict MSS when accounting for tumor thickness and age in stage II patients and may help in selection of adjuvant therapies.<br><br>Authors conclude 11-GEP independent predictor of MSS but in MVA Breslow depth and age also highly significant with tighter Cis for tumor thickness, therefore more significant P value<br>AND that patients with high score

						should have adjuvant systemic therapy which cannot be concluded from these data.<br><br>*Critiques of Methodology:*<br>Not adjusting for other factors such as ulceration<br>20% of patients did not undergo SLN biopsy.<br><br>20% of patients didn't have SLN surgery.<br><br>No information on characteristics of study cohort vs untested stage II patients.<br><br>Treatment with immune checkpoint blockade not controlled for.<br><br>Is 10 year endpoint valid for data set with 3.5 year median f/u?

Summary of Evidence Table<br>Question 1, 2						
Level of Evidence*^<br><br>Choose one:	Study Citation<br>Author(s), Year, Title, Journal, Volume, Page #s	Funding Source	Study Methodology	Objective & Methods	Results	Conclusions
Strong<br>☐EL 1; RCT<br>☐EL 1; MRCT<br><br>Intermediate<br>☐EL 2; MNRCT<br>☐EL 2; NMA<br>☐EL 2; NRCT<br>☐EL 2; PCS<br>☐EL 2; RCCS<br>☐EL 2; NCCS<br>☐EL 2; CSS<br>☐EL 2; ES<br>☐EL 2; OLES<br>☐EL 2; PHAS<br><br>Weak<br>☐EL 3; DS<br>☐EL 3; ECON<br>☒EL 3; CCS<br>☐EL 3; SCR<br>☐EL 3; PRECLIN<br>☐EL 3; BR<br><br>No Evidence<br>☐EL 4; NE<br>☐EL 4: O	Amaral T, Sinnberg T, Chatziioannou E, et al. Identification of Stage I/II Melanoma Patients at High Risk for Recurrence Using a Model Combining Clinicopathologic Factors with Gene Expression Profiling (CP_GEP). *Eur J Cancer.* 2023;182:155-162.<br><br>*Country:* Germany Germany/Netherlands	*Source:*<br>The study was partially funded by SkylineDx.<br><br>4 authors: SkylineDx employees<br><br>2 author: institutional funding from SkylineDx in relation to the submitted work.<br><br>2 author: Institutional financial support from Neracare<br><br>5 author: stock/ownership interest in SkylineDx<br><br>1 author: leadership SkylineDx<br><br>1 author: consulting or advisory role	*Methodology*: Blinded retrospective single-center of patients with stage I/II patients.<br><br>Single center RP review of a prospective database.	*Stated Objective:*<br>To validate CP-GEP for the identification of high-risk for disease recurrence stage I/II patients.<br><br>☐Prospective<br>☒Retrospective<br><br>*Study Population and Setting:*<br>Stage I/II, age >18, bw 2000-2017, with negative SLNB<br><br>Patients with Stage I/II melanoma, SLN negative<br><br>*N:* 543 patients with SLNB+ 83 control without SLNB, of which 80 analyzed<br><br>*Intervention*: CP-GEP with clinicopath features age and Breslow thickness with the expression of (ITGB3, PLAT, SERPINE2, GDF15, TGFBR1, LOXL4, CXCL8 and MLANA) corrected by (RLP0 and ACTB)<br><br>CP-GEP combined protocol to predict recurrence	*Results:*<br>W SLNB<br>CP-GEP stratified 424 patients (78% cohort) into RFS rates of 77.8% for high risk (195 patients) and RFS rate of 93% for low risk (229 patients). HR 3.53 (p, 0.001).<br><br>RFS stage I 90.7%, stage II 66.1%<br>DMFS stage I 96.0%, stage II 82.2%.<br>OS stage I 95.6%, stage II 79.0%<br><br>For stage I/II: CP-GEP classified 311 as high risk with HR 4.73 with p-value <0.001<br><br>Subgroup I/IIA: CP-GEP stratified low v high-risk with a HR of 3.53 (p-value <0.001).<br><br>RFS I/IIA for high-risk (195 patients) was 77.8% and 93.0% for low-risk.<br><br>W/o SLNB<br>Classified 11/80 high-risk which captured 6/7 recurrences.<br><br>The five-year RFS for stage I/II patients was 79.9%	*Describe conclusions relative to question:*<br><br>CP-GEP predicts high risk pt in stage I/II and could be used to replace SLNB and be used to guide adjuvant treatments.<br><br>CP-GEP could replace SLNB<br><br>CP-GEP enhances traditional histologic classification of patients.<br><br>*Critiques of Methodology:*<br>Group stages I/II and do not separate to be able to discern clinicopathologic characteristics vs CP-GEP. The stage I/II vs I/IIA

				Outcome Measures: Kaplan-Meier curves to determine prognostic value.<br>Primary endpoint was RFS also DMFS and OS.<br><br>Recurrence after negative SLNbx<br><br>*Follow-Up*: Median follow up of 83.63 months for patients with negative SLNB outcome<br><br>40.77 mo for patients wo SLNB<br><br>*Notes*	(95% CI: 76.0%e83.2%).<br>CP-GEP identified 311 patients as high risk for disease recurrence with a HR of 4.73 with a p-value <0.001, capturing 83 out of 98 reported relapses.<br>For subgroup stage I/IIA, CP-GEP was able to significantly stratify CPGEP<br>low-risk and high-risk patients with a HR of 3.53 (p value <0.001) for five-year RFS.<br>For stage I/IIA, the five-year RFS rates for CP-GEP high-risk patients were 77.8% (95% CI: 70.9%e83.3%) and 93.0% (95% CI: 88.5%e95.8%) for CP-GEP low-risk patients.<br>“Compared to AJCC low-risk (stage I/IIA) patients with an<br>RFS rate of 86.0% (95% CI: 82.0%e89.1%), CP-GEP was<br>able to split 195 high-risk patients who had a worse five year RFS<br>survival of 77.8%”<br><br>Notes: : Wo slnb high-risk curves are dramatically different, does this argue for doing a slnb.<br><br>Wo slnb 5/11 classified as high-risk without recurrence.<br><br>They group the analyses into “who would qualify for adjuvant therapy vs who wouldn’t” There is no	Initial curves again do not separate by histologic stage. Group “Stage I/II” together.

					subset analysis by the actual histologic stages which aids their analysis but doesn’t accurately reflect our ability to discern.	

	Summary of Evidence Table<br>Questions 1, 2					
Level of Evidence*^<br><br>Choose one:	Study Citation<br>Author(s), Year, Title, Journal, Volume, Page #s	Funding Source	Study Methodology	Objective & Methods	Results	Conclusions
Strong<br>☐EL 1; RCT<br>☐EL 1; MRCT<br><br>Intermediate<br>☐EL 2; MNRCT<br>☐EL 2; NMA<br>☐EL 2; NRCT<br>☒EL 2; PCS<br>☐EL 2; RCCS<br>☐EL 2; NCCS<br>☐EL 2; CSS<br>☐EL 2; ES<br>☐EL 2; OLES<br>☐EL 2; PHAS<br><br>Weak<br>☐EL 3; DS<br>☐EL 3; ECON<br>☐EL 3; CCS<br>☐EL 3; SCR<br>☐EL 3; PRECLIN<br>☐EL 3; BR<br><br>No Evidence<br>☐EL 4; NE<br>☐EL 4: O	Arnot SP, Han G, Fortino J, Han D, Fowler G, Vetto JT. Utility of a 31-Gene Expression Profile for Predicting Outcomes in Patients with Primary Cutaneous Melanoma Referred for Sentinel Node Biopsy. *Am J Surg* 2021;221(6):1195-1199. (71)<br><br>*Country:* USA	*Source:* Non-industry sponsored<br><br>1author declared an honorarium from Castle Biosciences.	*Methodology:* Retrospective cohort study (convenience study; not consecutive patients).<br><br>Prospective collection of GEP prior to operation then correlative work done.	*Stated Objective:* Assess outcomes in Class I vs Class II<br>Correlate clinical finding to GEP.<br><br>☒Prospective sample collection<br>☒Retrospective study of a prospective database<br><br>*Study Population and Setting:* Single institution<br>University setting<br><br>*N:* 383<br><br>*Intervention*:<br>31-GEP<br>GEP testing<br><br>*Outcome Measures:*<br>RFS, DMFS<br><br>*Follow-Up*: 32 mos (1-68)<br><br>*Notes:*	*Results:*<br><br>Class II had worse RFS, DMFS.<br><br>"Breslow thickness, T stage, and SNB positivity were significantly higher in Class 2 patients. Recurrence rates were higher for Class 2 vs Class 1 patients and highest in patients who were Class 2 and SNB positive. GEP class was predictive of RFS and DMFS and independently predicted relapse in AJCC "low risk" (stages IA-IIA) patients."<br><br>*Notes:* All included patients had 31-GEP and SLNB.	*Describe conclusions relative to question:*<br>31-GEP results added data for stage Ia and IIa patients.<br><br>Appropriate<br><br>*Critiques of Methodology:*<br>Selection bias; Appears to be a convenience sample and not all patients (consecutive) undergoing SLNB.<br><br>Correlative to RFS and DMFS.<br><br>Small series<br><br>Single institution<br><br>Retrospective analysis of a convenience sample.

	Summary of Evidence Table<br>Question 2					
Level of Evidence*^<br><br>Choose one:	Study Citation<br>Author(s), Year, Title, Journal, Volume, Page #s	Funding Source	Study Methodology	Objective & Methods	Results	Conclusions
☐EL 1; RCT<br>☐EL 1; MRCT<br><br>☐EL 2; MNRCT<br>☐EL 2; NMA<br>☐EL 2; NRCT<br>☐EL 2; PCS<br>☐EL 2; RCCS<br>☐EL 2; NCCS<br>☐EL 2; CSS<br>☒EL 2; ES<br>☐EL 2; OLES<br>☐EL 2; PHAS<br><br>☐EL 3; DS<br>☐EL 3; ECON<br>☐EL 3; CCS<br>☐EL 3; SCR<br>☐EL 3; PRECLIN<br>☐EL 3; BR<br><br>☐EL 4; NE<br>☐EL 4: O<br><br>Strong<br>Intermediate<br>Weak<br>No Evidence	[illegible]ation: Bailey CN, [illegible]rtin BJ, Petkov VI, et al. 31-Gene Expression Profile Testing in [illegible]taneous Melanoma [illegible]d Survival Outcomes [illegible] a Population-Based [illegible]alysis: A SEER [illegible]llaboration. JCO [illegible]ecis Oncol. 2023 [illegible]n;7:e2300044. Doi: 10.1200/PO.23.00044. PMID: 37384864.<br><br>*Country:* USA	*Source:*<br>Support-<br>: Iowa Cancer Registry, through National Cancer Institute, Surveillance, Epidemiology and End Results Program Contract Award No. HHSN261201800012I_HHSN26100001.<br><br>: Public Health Institute, Cancer Registry of Greater California, through National Cancer Institute, Surveillance, Epidemiology and End Results Program Contract Award No. HHSN261201800009I.<br><br>: HCI Cancer Control and Population Sciences Program, through National Cancer Institute, Surveillance, Epidemiology and End Results<br>Program Contract Award HHSN261201800016I<br><br>Kentucky Cancer Registry, through National Cancer Institute, Surveillance, Epidemiology and End Results Program Contract Award No. HHSN261201800013I<br><br>University of California, San Francisco, CA through National Cancer Institute, Surveillance, Epidemiology and End Results Program<br>Contract Award HHSN261201800032I	*Methodology:* Patients undergoing GEP testing were matched to seer data. MV analysis of GEP result was performed.<br><br>Linking of patients with 31-GEP test done 2016-2018 with SEER-17 data on MSS and OS and a propensity score matched cohort of non-tested patients was used for comparison.	*Stated Objective:* To evaluate ability of GEP to stratify MSS and OS. To assess the association of 31-GEP testing with survival.<br><br>investigate the prognostic capabilities of 31-GEP testing for melanoma survival outcomes using the SEER database, emphasizing its potential to categorize melanoma patients based on mortality risk.<br><br>☐Prospective<br>☒Retrospective<br><br>*Study Population and Setting*:<br><br>*N*: 4687 GEP tested patients, 3258 GEP tested patients were matched to 9774 non-GEP melanoma<br><br>4687 tested patients captured in SEER-17 2016-2018	*Results:* GEP was independently associated with MSS and OS in MV analysis. In a propensity-matched cohort of GEP to non-GEP tested patients, GEP testing was associated with improved MSS and OS compared to untested patients.<br><br>31-GEP testing stratified melanoma patients in terms of MSS<br>Class IA 3 yr MSS 99.7%<br>Class IB/2A 3 yr MSS 97.1%<br>Class 2B MSS 89.6%<br><br>*Notes:*<br>Conclusion: Due to significant limitations in study design and	*Describe conclusions relative to question:*<br><br>31-GEP successfully stratified patients by the risk of dying<br><br>31-GEP testing was associated with a 29% lower mortality from melanoma and 17% overall lower mortality<br><br>*Critiques of Methodology:*<br><br>Solid retrospective evidence of prognostic value, albeit with limited follow up. Causation vs. correlation in matched cohort.

		The Connecticut Tumor Registry is supported by Federal funds from the National Cancer Institute, National Institutes of Health, Department of Health and Human Services, under Contract no. HHSN261201800002I.<br><br>University of Hawaii Cancer Center, HI through National Cancer Institute, Surveillance, Epidemiology and End Results Program Contract Award HHSN261201300009I.<br>: Department of Population and Public Health Sciences, Keck School of Medicine, University of Southern California, Los Angeles, CA through National Cancer Institute, Surveillance, Epidemiology and End Results<br>Program Contract Award HHSN261201800032I<br><br>: Cancer Data Registry of Idaho, Idaho Hospital Association,<br>Boise, Idaho through National Cancer Institute, Surveillance, Epidemiology and End Results Program Contract Award HHSN261201800006I and Centers for Disease Control and Prevention 1NU58DP006270.<br><br>Bureau of Cancer Epidemiology, New York State Department of Health,<br>Albany, NY through National Cancer Institute, Surveillance, Epidemiology and End Results Program Contract Award HHSN261201800005I and Centers for Disease Control and Prevention NU58DP006309<br><br>: Division of Public Health Sciences Fred Hutchinson Cancer<br>Center, through National Cancer Institute, Surveillance, Epidemiology and		*Intervention:* 31-GEP<br>31 GEP vs no testing 2016-2018<br><br>*Outcome Measures:* MSS, OS<br><br>*Follow-Up:* Not specifically reported, limited to 3 years by inclusion criteria<br><br>*Notes:*	methodology, the conclusions of the research on 31-GEP's effectiveness are potentially weakened. More rigorous studies are essential to confirm the findings.	The study relied on a three-year f/u for survival outcomes, potentially skewing data due to the death of higher-stage patients within this short time frame. Absence of data on specific outcomes such as recurrence-free and distant metastasis-free survival. The study also lacked details on results specific to clinical substages and did not include clinical stage as a factor in multivariable analyses. Selection criteria for the 31-GEP patient cohort (comprising 4,687 individuals) is vague, raising concerns about selection bias and the cohort's representation of the wider melanoma patient demographic in the US.

		End Results Program Contract Award HHSN2612018000041<br><br>Emory University, Atlanta GA. through National Cancer Institute, Surveillance, Epidemiology and End Results Program Contract Award HHSN261201800003I and Centers for Disease Control and Prevention 6NU58DP006352-05-01<br><br>Emory University, Atlanta, GA, through National Cancer Institute, Surveillance, Epidemiology and End Results Program Contract Award HHSN261201800014I.<br><br>School of Public Health Louisiana State University Health New Orleans, through National Cancer Institute, Surveillance, Epidemiology and End Results Program Contract Award HHSN261201800007I/ HHSN26100002.<br><br>7 authors: employees Castle Biosciences<br>7 author: stock & other ownership interests Castle Biosciences<br><br>1 author: research funding Castle Biosciences<br><br>1 author: consulting or advisory role Castle Biosciences<br><br>3 author: patents, royalties, other intellectual property Castle Biosciences<br><br>3 author: travel, accommodations, expenses Castle Biosciences<br><br>1 author: leadership Castle Biosciences				

	Summary of Evidence Table<br>Question 2					
Level of Evidence*^<br><br>Choose one:	Study Citation<br>Author(s), Year, Title, Journal, Volume, Page #s	Funding Source	Study Methodology	Objective & Methods	Results	Conclusions
**Strong**<br>☐EL 1; RCT<br>☐EL 1; MRCT<br><br>**Intermediate**<br>☐EL 2; MNRCT<br>☐EL 2; NMA<br>☐EL 2; NRCT<br>☒EL 2; PCS<br>☐EL 2; RCCS<br>☐EL 2; NCCS<br>☐EL 2; CSS<br>☐EL 2; ES<br>☐EL 2; OLES<br>☐EL 2; PHAS<br><br>**Weak**<br>☐EL 3; DS<br>☐EL 3; ECON<br>☐EL 3; CCS<br>☐EL 3; SCR<br>☐EL 3; PRECLIN<br>☐EL 3; BR<br><br>**No Evidence**<br>☐EL 4; NE<br>☐EL 4: O	Berger AC, Davidson RS, Poitras JK, et al. Clinical impact of a 31-gene expression profile test for cutaneous melanoma in 156 prospectively and consecutively tested patients. *Curr Med Res and Opin*. 2016;32(9):1599-1604. (13)<br><br>*Country:* USA	*Source:*<br>Sponsored by Castle BioSciences, Inc. Participating institutions received financial compensation to account for costs associated with the conduct of the study.<br><br>6 authors: employees of Castle Biosciences and hold stock in company.<br><br>7 authors: rec'd sponsorship and research funding from Castle Biosciences.<br><br>3 authors: service on the speakers' bureau for Castle Biosciences	*Methodology*:<br><br>31-gene expression test<br><br>Retrospective multicenter cohort of consecutive patients tested for 31-GEP.	*Stated Objective:*<br><br>☒Prospective<br>☒Retrospective<br><br>Retrospective study of a prospectively tested patient population<br><br>*Study Population and Setting:*<br>Six centers (three surg, three derm) contributed cases of patients tested for 31-GEP as part of clinical mgmt.<br><br>*N:* 156 cutaneous melanoma 2013-2015<br><br>*Intervention:* 31-GEP<br>Chart review<br>Studied how GEP changed clinical management.<br><br>*Outcome Measures:*<br>Change in management strategy (more or less intensive follow up) from before to after GEP result.<br><br>Frequency of physical exam<br>Frequency of imaging<br>Referrals to surg onc/med onc<br><br>*Follow-Up:* None<br><br>*Notes:*	*Results:*<br>50% of patients who had a GEP test performed had management changed by treating physician as a result.<br><br>61% class 1<br>39% class 2<br><br>Median Breslow: 2mm<br><br>53% had a "documented change" in management following GEP.<br><br>37% of Class 1 changed (majority changed to reduced intensity management).<br><br>77% of Class 2 pts had documented changes in surveillance/referral, most with increased intensity of management.<br><br>72% had SLN (12% positive).<br><br>*Notes:* Study demonstrates the importance of blinding physicians to results in a prospective study.	*Describe conclusions relative to question:*<br><br>GEP does have impact on management.<br><br>GEP changed clinical management in more than half of tested pts; increased intensity of surveillance in high-risk class 2 and reduced intensity in low risk Class I.<br><br>*Critiques of Methodology:*<br><br>No outcome data.<br><br>Unable to state if the management changes made were "right."<br><br>Demonstrates no data to suggest that test results were correct or that

		1 peer reviewer=is a consultant to Castle Biosciences				management changes were beneficial to patients based on outcome.

	Summary of Evidence Table<br>Question 1, 2, 3					
Level of Evidence*^<br><br>Choose one:	Study Citation<br>Author(s), Year, Title, Journal, Volume, Page #s	Funding Source	Study Methodology	Objective & Methods	Results	Conclusions
Strong<br>☐EL 1; RCT<br>☐EL 1; MRCT<br><br>Intermediate<br>☐EL 2; MNRCT<br>☐EL 2; NMA<br>☐EL 2; NRCT<br>☐EL 2; PCS<br>☐EL 2; RCCS<br>☐EL 2; NCCS<br>☐EL 2; CSS<br>☐EL 2; ES<br>☐EL 2; OLES<br>☐EL 2; PHAS<br><br>Weak<br>☐EL 3; DS<br>☐EL 3; ECON<br>☐EL 3; CCS<br>☐EL 3; SCR<br>☐EL 3; PRECLIN<br>☐EL 3; BR<br><br>No Evidence<br>☒EL 4; NE<br>☐EL 4: O	Berman B, Ceilley R Cockerell C. Appropriate Use Criteria for the Integration of Diagnostic and Prognostic Gene Expression Profile Assays into the Management of Cutaneous Malignant Melanoma: An Expert Panel Consensus-Based Modified Delphi Process Assessment. *SKIN The Journal of Cutaneous Medicine*, 2019; 3(5): 291–306.<br><br>*Country:* USA	*Source:*<br><br>The National Society for Cutaneous Medicine (a 501(c)3 non-profit entity) funded the consensus development program. The group has received unrestricted educational grants from related companies in volved with these technologies.<br><br>5 authors: Castle Bioscience consultant<br><br>2 author: Castle Bioscience investigator<br><br>2 author: Castle Bioscience honoraria	*Methodology:*<br><br>Expert panel consensus based Modified Delphi Process<br><br>3 GEP tests evaluated: 2-GEP Test, myPath from Myriad, Castle 31-GEP<br><br>Literature review	*Stated Objective:*<br><br>The objective of this expert panel was to develop a set of consensus-based AUC recommendations to guide the integration of GEP technology into the diagnosis and management of melanoma<br><br>☐Prospective<br>☐Retrospective<br><br>*Study Population and Setting*: Lit review done (33 total articles)<br>Consensus panel was 9 derm/dermpath/surgeons<br><br>*N:*<br><br>*Intervention:*<br><br>*Outcome Measures:*<br><br>*Follow-Up:* None<br><br>*Notes:*	*Results:*<br>Several clinical scenarios evaluation and given consensus based recommendations based on the taxonomy below.<br><br>One of 14 scenarios received an A-strength recommendation: • Use of the 31-GEP test to aid in the management of patients who are SLNBx negative<br><br>Not applicable<br><br>*Notes:*<br>Based on SORT Taxonomy, A = Consistent, good-quality patient-oriented evidence, B = Inconsistent or limited quality patient-oriented evidence, C = Consensus, disease-oriented evidence, usual practice, expert opinion, or case series	*Describe conclusions relative to question:*<br><br>First "evidence based" framework for using GEP tests in clinical management.<br><br>*Critiques of Methodology:*

		3 author: Castle Bioscience advisory board				

	Summary of Evidence Table<br>Question 1, 2, 3					
Level of Evidence*^<br><br>Choose one:	Study Citation<br>Author(s), Year, Title, Journal, Volume, Page #s	Funding Source	Study Methodology	Objective & Methods	Results	Conclusions
Strong<br>☐EL 1; RCT<br>☐EL 1; MRCT<br><br>Intermediate<br>☐EL 2; MNRCT<br>☐EL 2; NMA<br>☐EL 2; NRCT<br>☐EL 2; PCS<br>☐EL 2; RCCS<br>☐EL 2; NCCS<br>☐EL 2; CSS<br>☐EL 2; ES<br>☐EL 2; OLES<br>☐EL 2; PHAS<br><br>Weak<br>☒EL 3; DS<br>☐EL 3; ECON<br>☐EL 3; CCS<br>☐EL 3; SCR<br>☐EL 3; PRECLIN<br>☐EL 3; BR<br><br>No Evidence<br>☐EL 4; NE<br>☐EL 4: O	Cook RW, Middlebrook B, Wilkinson J, et al. Analytic validity of DecisionDx-Melanoma, a gene expression profile test for determining metastatic risk in melanoma patients. *Diagn Pathol.* Feb 13, 2018;13(1):13.<br><br>*Country:* USA	*Source:*<br>This study was sponsored by Castle Biosciences<br><br>7 authors: employees of Castle Biosciences<br><br>7 authors: hold stock in the company	*Methodology:*<br><br>Lab validation study<br><br>Inter-assay, inter-instrument, and inter-operator studies to evaluate reliability of test results, sample stability and reagent stability.	*Stated Objective:*<br>Assess reproducibility of Decision DX assay<br><br>☐Prospective<br>☒Retrospective<br><br>*Study Population and Setting:*<br><br>*N:* 8244 samples run (March 2013-June 2016)<br>168 samples run twice to assess reproducibility<br><br>168 specimens (de-identified of patient info)<br><br>*Intervention:* none<br><br>*Outcome Measures:*<br>Reproducibility of Decision Dx assay<br><br>*Follow-Up:*<br><br>*Notes:* Based on existing fixed tissue samples	*Results:*<br>Among all samples, the technical success of the assay was 98% in the 85% of samples that met pre-specified tumor content parameters.<br><br>Concordance among assays (in 168 subset of samples for which repeat assay was performed) on two consecutive days was 99%. Inter-instrumental concordance was 95%.<br><br>Analytic validity based on inter-assay, inter-operator, and inter-instrument reliability measurements show technical success of the test (99% rate).<br><br>*Notes:*	*Describe conclusions relative to question:*<br><br>The assay is reliable and reproduceable.<br><br>*Critiques of Methodology:*

Summary of Evidence Table<br>Question 2						
Level of Evidence*^<br><br>Choose one:	Study Citation<br>Author(s), Year, Title, Journal, Volume, Page #s	Funding Source	Study Methodology	Objective & Methods	Results	Conclusions
Strong<br>☐EL 1; RCT<br>☐EL 1; MRCT<br><br>Intermediate<br>☐EL 2; MNRCT<br>☐EL 2; NMA<br>☐EL 2; NRCT<br>☐EL 2; PCS<br>☒EL 2; RCCS<br>☐EL 2; NCCS<br>☐EL 2; CSS<br>☐EL 2; ES<br>☐EL 2; OLES<br>☐EL 2; PHAS<br><br>Weak<br>☐EL 3; DS<br>☐EL 3; ECON<br>☐EL 3; CCS<br>☐EL 3; SCR<br>☐EL 3; PRECLIN<br>☐EL 3; BR<br><br>No Evidence<br>☐EL 4; NE<br>☐EL 4: O	*Citation:*<br>Dhillon S, Duarte-Bateman D, Fowler G, et al. Routine imaging guided by a 31-gene expression profile assay results in earlier detection of melanoma with decreased metastatic tumor burden compared to patients without surveillance imaging studies. *Arch Dermatol Res.* 2023;28:28.<br><br>*Country:* USA	*Source:* This work was supported by the IDP Foundation, Inc.<br><br>3 authors: served as consultants for Castle Biosciences and received honoraria for their work<br><br>This study was performed independent of Castle Biosciences and was not supported or funded by them in many manner.	*Methodology:*<br><br>All patients from three centers with a neg SLNB were potentially included: Patients with high risk GEP and followed by imaging were compared to those without GEP who were not followed by imaging.<br><br>Retrospective review of selected SLN negative melanoma patients comparing a group of high-risk 31-GEP but SLN negative patients who had scheduled routine imaging surveillance for recurrence to a group of SLN negative patients who did not have 31-GEP testing	*Stated Objective:*<br><br>☐Prospective<br>☒Retrospective<br><br>*Study Population and Setting:*<br><br>*N:* 327 control 307 cases, 28 recurrent control without imaging, 38 recurrent GEP with imaging<br><br>*Intervention:* GEP and imaging<br><br>*Outcome Measures:* Time to recurrence, tumor burden Recurrence, time to recurrence<br><br>*Follow-Up:* 46 months in living recurrent pts<br><br>45.6 mos in exp group vs 63.3 mos in control group<br><br>*Notes:* Listed as RCCS but not clear if a representative cohort from which cases/controls were drawn<br><br>Imaging protocols varied across sites – typical CTC/A/P + brain MRI q 6 mos but not uniform	*Results*: 20% recurrence in high risk GEP compared to 14% all patients without imaging. Tumor burden 28mm when imaging and gep used vs. 73 mm in control. Median time to recurrence 10 mon earlier in image/gep group<br><br>*Notes:* Unadjusted survival data presented	*Describe conclusions relative to question:*<br><br>GEP can guide imaging which can in turn identify earlier recurrence which in turn can improve patient outcomes.<br><br>Overstated conclusions.<br><br>Using high risk 31-GEP to determine imaging surveillance protocol results in earlier detection of recurrence and better patient outcomes<br><br>Lower tumor burden measured in mm – what is the clinical relevance of that?<br><br>*Critiques of Methodology:*<br><br>Selection bias in who was selected

			and did not have routine imaging surveillance for recurrence.			for follow up without imaging. Unclear if imaging alone without GEP would have had same results.<br><br>Retrospective<br>Differing f/u<br>Uncontrolled survival analysis<br>Excluded 25 patients from experimental group who recurred but didn't follow imaging protocol

	Summary of Evidence Table<br>Question 1, 2					
Level of Evidence*^<br><br>Choose one:	Study Citation<br>Author(s), Year, Title, Journal, Volume, Page #s	Funding Source	Study Methodology	Objective & Methods	Results	Conclusions

☐EL 1; RCT<br>☐EL 1; MRCT<br>(Strong)<br><br>☐EL 2; MNRCT<br>☐EL 2; NMA<br>☐EL 2; NRCT<br>☐EL 2; PCS<br>☐EL 2; RCCS<br>☐EL 2; NCCS<br>☐EL 2; CSS<br>☐EL 2; ES<br>☐EL 2; OLES<br>☐EL 2; PHAS<br>(Intermediate)<br><br>☐EL 3; DS<br>☐EL 3; ECON<br>☐EL 3; CCS<br>☐EL 3; SCR<br>☐EL 3; PRECLIN<br>☐EL 3; BR<br>(Weak)<br><br>☒EL 4; NE<br>☐EL 4: O<br>(No Evidence)	Farberg AS, Glazer AM, White R, Rigel DS. Impact of a 31-gene Expression Profiling Test for Cutaneous Melanoma on Dermatologists' Clinical Management Decisions. *J Drugs Dermatol*: 2017;16(5):428-431. (16)<br><br>*Country*: USA	*Source:*<br>None noted<br><br>2 authors=consultants to Castle Biosciences, Inc.<br><br>1=author participated in research fellowship partially funded by Castle Biosciences.	*Methodology:*<br><br>Gave Derm residents patient vignettes to assess clinical decision making, incorporating 31-GEP. They were given a presentation before on "clinical validity" for GEP.<br><br>Survey study of dermatology residents provided case-based scenarios involving management of 31-GEP results.	*Stated Objective:*<br>To determine impact of 31-GEP test on clinical decision making.<br><br>☒Prospective<br>☐Retrospective<br><br>*Study Population and Setting:*<br>Survey study of case-based scenarios provided to dermatology residents.<br><br>Derm residents at a national conference given clinical scenarios involving 31-GEP test (Stage IA, IB, IIA pts).<br><br>*N:* 169 dermatology resident physicians of 172 attendees responded to survey questions<br><br>*Intervention:* 31-GEP results given or not with clinical info.<br><br>Six cutaneous melanoma cases with GEP information varied.<br><br>*Outcome Measures:*<br>Management decisions (SLN bx, onc referral)<br><br>Clinical recommendation<br><br>*Follow-Up:* N/A<br><br>*Notes:*	*Results:*<br>Approximately 50% of respondents changed clinical decision making when faced with a Class II GEP result.<br><br>Most respondents gave 1.0 mm Breslow as guiding modality for SLNbx.<br><br>Basically states that when given 31-GEP results, specifically class 2 result, Breslow depth to guide SLNbx changed (more likely to refer thinner Breslow depth if high risk GEP class). Also more likely to recommend imaging.<br><br>*Notes:*	*Describe conclusions relative to question:*<br>"GEP result had a significant and appropriate impact on clinical decision making" (there was no evidence provided that this was appropriate).<br><br>The 31-GEP test had a "significant and appropriate" impact on management while remaining within established guidelines.<br><br>*Critiques of Methodology:*<br>Provides no information regarding validity of GEP<br><br>Were they trying to get the "right" answer based on who was presenting info to them?<br>-Who gave the lecture on clinical validity of the test before the study??- that could significantly bias results

						-Authors do not recognize these limitations in the manuscript<br>-They gave the residents an answer of “onc” and imaging referral which would not be currently recommended for the thin Breslow depths noted; confusing

Summary of Evidence Table<br>Question 1, 2, 3						
Level of Evidence*^<br>Choose one:	Study Citation<br>Author(s), Year, Title, Journal, Volume, Page #s	Funding Source	Study Methodology	Objective & Methods	Results	Conclusions
☐EL 1; RCT<br>☐EL 1; MRCT<br>Strong<br><br>☐EL 2; MNRCT<br>☐EL 2; NMA<br>☐EL 2; NRCT<br>☐EL 2; PCS<br>☐EL 2; RCCS<br>☐EL 2; NCCS<br>☐EL 2; CSS<br>☐EL 2; ES<br>☐EL 2; OLES<br>☐EL 2; PHAS<br>Intermediate<br><br>☐EL 3; DS<br>☐EL 3; ECON<br>☐EL 3; CCS<br>☐EL 3; SCR<br>☐EL 3; PRECLIN<br>☐EL 3; BR<br>Weak<br><br>☒EL 4; NE<br>☐EL 4: O<br>No Evidence	Farberg AS, Marson JW, Glazer A, et al. Expert Consensus on the Use of Prognostic Gene Expression Profiling Tests for the Management of Cutaneous Melanoma: Consensus from the Skin Cancer Prevention Working Group. *Dermatol Ther.* 2022;12(4):807-823.<br>(81)<br><br>*Country:* USA	*Source:*<br>No funding or sponsorship was received for this study or publication of this article.<br><br>3 authors=consultants for Castle Biosciences<br><br>1 author=advisory board member and speaker for Castle Biosciences	*Methodology:*<br>Modified Delphi technique for consensus statements.<br><br>Review – no new data.<br><br>Skin Cancer Prevention Working Group are authors.	*Stated Objective:*<br>Assess applications for GEP in melanoma management.<br><br>☐Prospective<br>☐Retrospective<br><br>*Study Population and Setting:*<br><br>*N:*<br><br>*Intervention:*<br><br>*Outcome Measures:*<br><br>*Follow-Up:*<br><br>Notes:	*Results:*<br>Consensus statements support role for GEP above and beyond AJCC and NCCN.<br><br>*Notes:*<br>Consensus statements support role for GEP above and beyond AJCC and NCCN.<br>The statements are non-specific.	*Describe conclusions relative to question:*<br><br>Consensus statements support role for GEP.<br><br>"GEP tests provide additional, reproducible information for dermatologists to consider within the larger framework of the eighth edition of the AJCC and NCCN cutaneous melanoma guidelines when counseling regarding prognosis and when considering a sentinel lymph node biopsy."<br><br>*Critiques of Methodology:*<br>No formal accompanying systematic review/meta-analysis to support the consensus

						process (only 8 panelists).<br><br>Review of existing data.

	Summary of Evidence Table<br>Question 2,3					
Level of Evidence*^<br><br>Choose one:	Study Citation<br>Author(s), Year, Title, Journal, Volume, Page #s	Funding Source	Study Methodology	Objective & Methods	Results	Conclusions
Strong<br>☐EL 1; RCT<br>☐EL 1; MRCT<br><br>Intermediate<br>☐EL 2; MNRCT<br>☐EL 2; NMA<br>☐EL 2; NRCT<br>☐EL 2; PCS<br>☐EL 2; RCCS<br>☐EL 2; NCCS<br>☐EL 2; CSS<br>☐EL 2; ES<br>☐EL 2; OLES<br>☐EL 2; PHAS<br><br>Weak<br>☐EL 3; DS<br>☐EL 3; ECON<br>☒EL 3; CCS<br>☐EL 3; SCR<br>☐EL 3; PRECLIN<br>☐EL 3; BR<br><br>No Evidence<br>☐EL 4; NE<br>☐EL 4: O	Ferris LK, Farberg AS, Middlebrook B, et al. Identification of high-risk cutaneous melanoma tumors is improved when combining the online American Joint Committee on Cancer Individualized Melanoma Patient Outcome Prediction Tool with a 31-gene expression profile-based classification. *J Am Acad Dermatol*. May 2017;76(5):818-825.e3.<br><br>*Country:* USA	*Source:* Funded by Castle Biosciences which provided financial compensation to those centers contributing cutaneous melanoma tissue to the study.<br><br>6 authors: employees of Castle Biosciences<br><br>4 authors: served as consultants to Castle Bioscience	*Methodology:* RNA isolated to run 31 gene test.<br><br>Five-year survival for each patient using AJCC Individualized Melanoma Patient Outcome Prediction Tool<br>Survival cutoff for low and high-risk determined by stage IIA (79%) and stage IIB (68%) cohorts.<br><br>K-M analysis and Cox proportional hazards survival analysis performed.<br><br>205 cases of stage I/II CM with available tissue were collected from six US centers.	*Stated Objective:* To compare accuracy of GEP with risk determined using the web-based AJCC Individualized Melanoma Patient Outcome Prediction Tool.<br><br>Compare recurrence risk prediction by AJCC Stage vs. 31-GEP class.<br><br>☐Prospective<br>☒Retrospective<br><br>*Study Population and Setting:* Stage I/II CM. 205 retrospectively collected.<br><br>*N:* 205<br><br>*Intervention:* GEP and AJCC prediction tool<br>GEP vs conventional staging<br><br>*Outcome Measures:* RFS, DMFS, OS<br><br>*Follow-Up:* 6.9 y<br>f/u 6.9 years (0.1-15.4)<br><br>*Notes:*	*Results:* 43 (21%) cases had discordant GEP and AJCC classification (with 79% cutoff). 11/43 discordant cases classified as high risk GEP but low by AJCC.<br><br>Sensitivity GEP 82%, 81% and 78% for RFS, DMFS and MSS. AJCC 70%, 69% and 60%.<br><br>Specificity<br>GEP 77%, 69%, 69% and AJCC 83%, 76%, 74%<br><br>Increased sensitivity in combining GEP + AJCC with decreased specificity.<br><br>Multivariate cox regression GEP w AJCC tool indicated GEP more significantly associated with DM and death than binary AJCC.<br><br>HR GEP vs AJCC 79%<br>Recurrence- 5.9 v 3.6<br>DMFS- 5.3 v 3.0,<br>Death -5.3 vs 2.2<br><br>Use of GEP test with AJCC stage improved sensitivity to detect recurrence and to a lesser degree death at the cost of specificity compared with AJCC alone.	*Describe conclusions relative to question:*<br><br>To combine GEP with AJCC may help identify pt benefit from increased surveillance and administer therapies.<br><br>GEP can improve detection of recurrence/mets when used in combination with AJCC.<br><br>Somewhat overstated based on limitations of dataset.<br><br>*Critiques of Methodology:* Using AJCC tool as binary high-risk vs low-risk to fit comparison.<br><br>Unconventional and narrow to define sensitivity and specificity of

					Notes:	test using definition of low-risk group from stage IIA AJCC 79% survival and high-risk 68% survival based off of stage IIB.<br><br>These patients were previously published in the development of the model. Unclear how this subset was selected. Stage was dichotomized at IIA- vs IIB+ somewhat arbitrarily.

Summary of Evidence Table<br>Question 2						
Level of Evidence*^<br><br>Choose one:	Study Citation<br>Author(s), Year, Title, Journal, Volume, Page #s	Funding Source	Study Methodology	Objective & Methods	Results	Conclusions
Strong<br>☐EL 1; RCT<br>☐EL 1; MRCT<br><br>Intermediate<br>☐EL 2; MNRCT<br>☐EL 2; NMA<br>☐EL 2; NRCT<br>☐EL 2; PCS<br>☒EL 2; RCCS<br>☐EL 2; NCCS<br>☐EL 2; CSS<br>☐EL 2; ES<br>☐EL 2; OLES<br>☐EL 2; PHAS<br><br>Weak<br>☐EL 3; DS<br>☐EL 3; ECON<br>☐EL 3; CCS<br>☐EL 3; SCR<br>☐EL 3; PRECLIN<br>☐EL 3; BR<br><br>No Evidence<br>☐EL 4; NE<br>☐EL 4: O	Gastman BR, Gerami P, Kurley SJ, Cook RW, Leachman S, Vetto JT. Identification of Patients at Risk of Metastasis Using a Prognostic 31-Gene Expression Profile in Subpopulations of Melanoma Patients with Favorable Outcomes by Standard Criteria. *J Am Acad Dermatol.* 2019;80(1):149-157.e144.<br>(39)<br><br>*Country:* USA	*Source:*<br><br>Sponsored by Castle Biosciences, Inc, which provided funding for tissue and clinical data retrieval to contributing centers.<br><br>2 authors=employees of Castle Biosciences<br><br>3 authors: members of speakers' bureau for Castle Biosciences<br><br>2 authors: options holders for Castle Biosciences	*Methodology:*<br><br>Paraffin-embedded primary CM tumor tissue was obtained from 18 centers of CM stage I-III diagnosed bw (1998-2014). Case report form submitted with clinical, pathologic and outcomes data. Censor date Oct 2016. 31-GEP used to determine molecular profile of each sample. Analysis using Kaplan-Meier and Cox methods.<br><br>Multicenter study. Archived tissue tested using GEP and then survival outcomes stratified by GEP class.	*Stated Objective:*<br><br>Prognostic biomarker study.<br><br>To assess risk prediction by 31-GEP test within low-risk (AJCC) patients (SLN -, stage I-IIA, thin ≤ 1 mm).<br><br>Identify high-risk patients using 31-GEP from traditionally low-risk patient population.<br><br>☐Prospective<br>☒Retrospective<br><br>*Study Population and Setting:*<br>CM stage I-III diagnosed bw (1998-2014) multicenter (18 institutions)<br><br>Primary cutaneous melanoma patients.<br>*N:* 690<br>*Intervention:* 31-GEP GEP profiling of primary<br><br>*Outcome Measures:*<br><br>Recurrence free survival (RFS) and Distant metastasis-free survival (DMFS). Secondary endpoint Melanoma Specific Survival	*Results:*<br>Class 2B identifies putatively low risk patients at high risk of DSS/DMFS and MSS event compared to other subgroups.<br><br>Identification 70% SLN-negative patients who have metastasis as Class 2.<br><br>Reduced RFS Class 2B v Class 1A (p<0.001).<br><br>Class 1A had higher RFS, DMFS, and MSS rates compared to Class 2B p<0.0001.<br><br>Multivariate analysis, molecular class and SLN positivity were independent predictors of RFS, DMFS, and MSS. Ulceration significant for DMFS and thickness significant for RFS and DMFS.<br><br>Class 2B 31-GEP, positive node and thickness were independent predictors of MSS (p<0.05).<br><br>259 SLN-negative/Class 2B had worse RFS, DMFS, and MSS than SLN-neg/ Class 1A. (<0.01)<br><br>SLN-neg, Class 2 identified 71.3%, 70.4% and 78.6% of recurrences, mets and	*Describe conclusions relative to question:*<br><br>31-GEP identifies high-risk patients within low risk AJCC stage I-IIA and thin tumors.<br><br>31-GEP useful for personalized clinical decisions to identify high-risk CM patients.<br><br>2B seems to be surrogate marker for poorly differentiated tumors, since ulcerations status and MR drop out of the Cox-Regression analysis.<br>Breslow and SN status remain independent predictors of outcome, GEP DOES NOT replace these variables.<br><br>..........

				Recurrence-free survival – primary<br>DMFS and DSS - secondary<br><br>*Follow-Up:*<br>At least 5-years or documented recurrence<br><br>Minimum 5 years or event<br><br>*Notes:*	melanoma-specific mortality events.<br><br>-31-GEP Class 2A not significant predictor RFS, and DMFS.<br><br>Stage I - IIA<br>Class IA had significantly better 5-year RFS, DMFS and MSS than 2B (p<.0001).<br><br>For stage I-IIA, 31-GEP class 2B most significant predictor of RFS and DMFS in multivariate analysis including thickness, ulceration, and mitotic rate. Thickness was only significant for RFS.<br><br>Multivariate analysis for MSS and only Class 2 significant predictor of MSS.<br><br>Majority thin tumors T1 were low-risk Class 1 89.3% (251/281) however 5.3% Class 2 B. (2.0% of T1a and 13.9% of T1b).<br><br>Thin tumor Class 1A v 2B RFS 96.8% and 64.6% respectively (p<0.001). DMFS 97.2% v 84.4% (p=0.007). Only one death in this group so MSS not done.<br><br>Thin tumor Cox multivariate analysis of thickness, mitoses, ulceration and SLNB positivity showed 31-GEP Class 2B only independent and significant predictor of	*Critiques of Methodology:*<br>Retrospective<br>-size of cohort especially as analyze smaller groups such as in stage or ulceration and fewer events.<br><br>31-GEP most significant single variable for predictor but what about combination of thickness, ulceration and mitosis as is clinically used. Limitations discussed included incomplete pathologic staging data owing to variation in reporting standards.<br><br>Only 429 (63%) staged with SNB.<br><br>Authors have conflict of interest – on advisory board for Castle Bioscience.

					RFS. n=57; HR 9.34, p=.004).<br><br>*Notes:*	

Level of Evidence*^ Choose one:	Summary of Evidence Table Question 2 Study Citation Author(s), Year, Title, Journal, Volume, Page #s	Funding Source	Study Methodology	Objective & Methods	Results	Conclusions
Strong: ☐EL 1; RCT<br>☐EL 1; MRCT<br><br>Intermediate: ☐EL 2; MNRCT<br>☐EL 2; NMA<br>☐EL 2; NRCT<br>☐EL 2; PCS<br>☒EL 2; RCCS<br>☐EL 2; NCCS<br>☐EL 2; CSS<br>☐EL 2; ES<br>☐EL 2; OLES<br>☐EL 2; PHAS<br><br>Weak: ☐EL 3; DS<br>☐EL 3; ECON<br>☐EL 3; CCS<br>☐EL 3; SCR<br>☐EL 3; PRECLIN<br>☐EL 3; BR<br><br>No Evidence: ☐EL 4; NE<br>☐EL 4: O	Gastman BR, Zager JS, Messina JL, et al. Performance of a 31-gene Expression Profile Test in Cutaneous Melanomas of the Head and Neck. *Head & Neck.* 2019;41(4):871-879.<br>(40)<br><br>*Country:*<br>USA	*Source:*<br><br>Sponsored by Castle Biosciences,Inc. which provided funding for tissue and clinical data retrieval to contributing centers.<br><br>3 authors: employees of Castle Biosciences<br><br>2 authors: stock option holders of Castle Biosciences<br><br>2 authors: consultants for Castle Biosciences<br><br>3 authors: on the speaker bureau for Castle Biosciences	*Methodology:*<br>Head and neck CM, SLN-negative, stage I-III and thin tumor patients classified with 31-GEP test.<br><br>Survival analysis using Kaplan-Meier and Cox methods.<br><br>Primaries tested for GEP-31 profile using archival tissue.	*Stated Objective:*<br>Report subgroup analysis from prior validation studies using GEP classification in DM of head and neck to classify as low-risk or high-risk.<br><br>☐Prospective<br>☒Retrospective<br><br>*Study Population and Setting:*<br>Head and Neck Primary CM treated at academic cancer centers<br><br>Head and neck CM, SLN-negative, stage I-III and thin tumor patients from 16 centers diagnosed 1999-2011.<br><br>Excluded pt < 18 and another malignancy.<br><br>Exclusive of training set used in test's algorithm but previously analyzed in broader cohorts but not in head and neck subgroup as in this study.<br><br>*N*: 157<br><br>*Intervention:* 31-GEP GEP-testing of primary SNB staging of patients<br><br>*Outcome Measures:*	*Results:*<br><br>GEP class, SNB status and Breslow thickness were independent predictors of outcome.<br>Survival curves of SN+ve and Class 2B patients very similar.<br>Breslow most important predictor of survival.<br><br>79 patients (50%) low-risk Class 1A -60, Class 1B- 19 Class 2A -19, Class 2B 59<br><br>5-year RFS, DMFS, OS, MSS<br>Class 1A: 80%, 83%, 97% and 98%; Class 2B 25%, 33%, 43% and 61% (p<.001)<br><br>Univariate Cox proportional hazard models with Breslow thickness, ulceration, node positivity, and GEP class 2 results significant predictors of recurrence, DM, all-cause death and MS death (<.01).<br><br>Multivariate analysis, Breslow thickness independent predictor of all survival endpoints (p<.03), Node positivity and Class 2 independent predictors of recurrence (p=.02 and	*Describe conclusions relative to question:*<br><br>Would seem to indicate that GEP is surrogate for differentiation of tumor i.e. ulceration status and mitotic rate.<br><br>GEP is independent predictor of outcome.<br><br>No mention of therapeutic role of SLNB procedure for intermediate thickness melanoma.<br><br>31-GEP identifies high-risk patients likely to experience recurrence or die of melanoma within low-risk groups of SLN-negative, stage I-IIA and thin tumor patients.<br><br>*Critiques of Methodology:*<br>Retrospective

				Primary: RFS, DMFS, OS, MSS<br>Secondary endpoint: 31-GEP predicted outcome in combination with node status to determine prognostic value added.<br><br>DSS/DMFS/DSS & OS<br><br>*Follow-Up:*<br>≥ 5 years required, median length 7.1 years<br><br>5 years or event<br><br>*Notes:*	.01). Class 2 predictor of DM (p=.04).<br><br>Binary GEP combined with node status for Kaplan-Meier curves. Class 1/ node-negative v Class 2 / node negative RFS 83% v 37%, DMFS 85% v 45%, OS 91% v 62% and MSS ws 96% v 78%.<br><br>Class 1/node-positive v Class 2/node positive RFS 29% v 19%, DMFS 43% v 23%, OS 100% v 36% and MSS ws 100% v 50%.<br><br>Molecular class sensitivity for prediction recurrence, DM, death any cause, and melanoma-specific death 74%, 74%, 80%, and 88%. compared to node status 41%, 40%, 43%, and 52%.<br><br>NPV molecular class 76%, 78%, 87%, and 96% compared to node status 64%, 67%, 76%, and 90%.<br><br>Combining molecular and node increased the accuracy of identifying high-risk sensitivity of 81%, 80%, 82% and 88%.<br>NPV 81%, 82%, 88% and 96%.<br><br>Notes:<br>Figure 2 Class1/node+ 1 event and 0 events	Size of cohort too small for subgroup analysis<br><br>Limitation discussed in paper is low sample size impacting multivariate analysis w low MSS events corrected by using only GEP class and AJCC stage but then doesn't control for all clinicopathologic factors.<br><br>Statement that this cohort has some features more aggressive than clinical population should be expounded upon<br><br>What does that say of selection bias<br><br>Same cohort has been used in Gastman BR, Gerami P, Kurley SJ, Cook RW, Leachman S, Vetto JT. Identification of patients at risk of metastasis using a prognostic 31-gene expression

						profile in subpopulations of melanoma patients with favorable outcomes by standard criteria. JI Am Acad Dermatol. 2019;80(1):149-157.e144<br><br>Small cohort of patients<br><br>Specificity and sensitivity data quoted for outcome? appropriate?

Summary of Evidence Table<br>Question 2						
Level of Evidence*^<br><br>Choose one:	Study Citation<br>Author(s), Year, Title, Journal, Volume, Page #s	Funding Source	Study Methodology	Objective & Methods	Results	Conclusions
**Strong**<br>☐EL 1; RCT<br>☐EL 1; MRCT<br><br>**Intermediate**<br>☐EL 2; MNRCT<br>☐EL 2; NMA<br>☐EL 2; NRCT<br>☐EL 2; PCS<br>☒EL 2; RCCS<br>☐EL 2; NCCS<br>☐EL 2; CSS<br>☐EL 2; ES<br>☐EL 2; OLES<br>☐EL 2; PHAS<br><br>**Weak**<br>☐EL 3; DS<br>☐EL 3; ECON<br>☐EL 3; CCS<br>☐EL 3; SCR<br>☐EL 3; PRECLIN<br>☐EL 3; BR<br><br>**No Evidence**<br>☐EL 4; NE<br>☐EL 4: O	Gerami P, Cook RW, Russell MC, et al. Gene expression profiling for molecular staging of cutaneous melanoma in patients undergoing sentinel lymph node biopsy. *J Am Acad Dermatol*. 2015;72(5):780-785.e783.<br>(7)<br><br>*Country*: USA	*Source:*<br><br>Partially supported by Castle BioSciences Inc.<br><br>4 authors: employees of Castle Biosciences<br><br>3 authors: consultants to Castle Biosciences<br><br>3 authors: speakers for Castle Biosciences	*Methodology:*<br><br>Case series of patients with GEP 31-GEP result.<br><br>RT-PCR; 31 genes.<br><br>SLN outcome from clinical data.<br><br>DFS<br>DMFS<br>OS	*Stated Objective:* Evaluate prognostic value of GEP in combination with SLNB.<br><br>☐Prospective<br>☒Retrospective<br><br>*Study Population and Setting:* Multicenter retrospective cohort of patients with GEP available. Patients who underwent SLNB and had at least 5 years follow up were included.<br><br>*N*: 217 cutaneous melanoma undergoing SLNbx.<br><br>1998-2009<br><br>*Intervention:*<br>Castle 31 gene GEP<br><br>*Outcome Measures:*<br>DFS<br>DMFS<br>OS<br><br>*Follow-Up:*<br>Not reported in abstract.<br><br>*Notes:* This cohort is a subset of pts from a previously published cohort. Not clearly stated.	*Results:*<br>In patients with both GEP class and SLN status available, GEP class II was associated with adverse outcomes in both SLN+ and SLN- patients.<br><br>SLNB+=58/217<br>SLNB- 159/217<br><br>37 of 58 SLNB+ developed a metastatic event.<br><br>70 of 159 SLNB- developed a metastatic event.<br><br>Class 2 high risk: 141<br>Class I low risk: 76<br><br>91 of 141 Class 2 to metastatic disease.<br><br>16 of 76 Class 1 to metastatic disease.<br><br>PPV of SLNB for predicting distant mets: 55% and NPV 67%.<br><br>PPV GEP for distant mets: 50% and NPV 82%.<br><br>OS at 5 years for SLNB+ 62%; 70% for SLN-.<br><br>SLNB+ and GEP class 2 were predictors of DFS, DMFS, OS.<br><br>GEP with SLNB:	*Describe conclusions relative to question:*<br><br>In combination with SLNB, GEP will help identify high risk SLN-patients.<br><br>GEP an objective tool to predict metastatic risk in SLNB-eligible pts.<br><br>GEP outcome a more significant and better predictor of each endpoint compared to SLNB.<br><br>With SLNB, GEP improved prognostication.<br><br>*Critiques of Methodology:*<br>Bi-variate analysis (SLN and GEP only). Limited sample size.<br><br>Their SLN- group had a 30% risk of metastatic

					Class 1/SLNB- (67 pts): DFS and DMFS 83% and 86%.<br><br>Class 1/SLNB+ (9 pts): DFS 53% DMFS 53% OS 77%.<br><br>Class 2/SLNB+ (49 pts): DFS 33% DMFS 42%, OS 57%.<br><br>Class 2/SLNB (58): DFS 35%, DMFS 49%, OS 55%.<br><br>*Notes:*	events which is high – lower OS also than expected.<br><br>Time span is older cohort (early 2000's).<br><br>The Breslow depth for SLN- was 2.3 (fairly high risk group to begin with); the SLNB was 4mm- thick melanomas. Would be better to have more detailed TNM staging described instead of how they presented it and more clinicopathologic features (site of melanoma etc)..

	Summary of Evidence Table<br>Question 2					
Level of Evidence*^<br><br>Choose one:	Study Citation<br>Author(s), Year, Title, Journal, Volume, Page #s	Funding Source	Study Methodology	Objective & Methods	Results	Conclusions
☐EL 1; RCT<br>☐EL 1; MRCT<br>Strong<br><br>☐EL 2; MNRCT<br>☐EL 2; NMA<br>☐EL 2; NRCT<br>☐EL 2; PCS<br>☐EL 2; RCCS<br>☐EL 2; NCCS<br>☐EL 2; CSS<br>☐EL 2; ES<br>☐EL 2; OLES<br>☐EL 2; PHAS<br>Intermediate<br><br>☒EL 3; DS<br>☐EL 3; ECON<br>☐EL 3; CCS<br>☐EL 3; SCR<br>☐EL 3; PRECLIN<br>☐EL 3; BR<br>Weak<br><br>☐EL 4; NE<br>☐EL 4: O<br>No Evidence	Gerami P, Cook R, Wilkinson J, et al. Development of a prognostic genetic signature to predict the metastatic risk associated with cutaneous melanoma. *Clin Cancer Res*. 2015 Jan 1;21(1):175-83 (106)<br><br>*Country:* USA	*Source:*<br><br>Castle Biosciences, Inc. and with support from the Irene D. Pritzker Foundation.<br><br>The costs of publication were defrayed in part by the payment of page charges; article must be marked advertisement in accordance with law.<br><br>4 authors: employees of Castle Biosciences<br><br>1 author: consultant for Castle Biosciences<br><br>3 authors:have ownership interests in Castle Biosciences	*Methodology:* 28-gene signature developed from microarray data from a multicenter cohort with development and validation cohorts.<br><br>FFPE tissue from 6 US institutions' Stage I-IV melanoma patients with 5 years minimum f/u or metastatic event + deidentified clinical data diagnosed 1998-2009.	*Stated Objective:* "the novel signature can accurately determine metastatic risk profile for cutaneous melanoma tumors and adds value to current AJCC staging methods that can overlook potential for metastasis in low-stage, sentinel node–negative cutaneous melanoma tumors."<br><br>☐Prospective<br>☒Retrospective<br><br>*Study Population and Setting:* 6 center multicenter cohort 6 US melanoma center patients Stage I-IV.<br><br>*N:* 107 initial development set (stage I, II)<br>Ultimately 268 patients stage 0-IV included from 7 institutions; validation set N=104<br><br>Intervention: 28 GEP signature (+3 control genes) evaluation by RT-PCR in primary melanoma + radial basis machine learning modeling to test Class I vs Class 2.<br><br>Outcome Measures: DFS from time of diagnosis.	*Results:*<br><br>DFS rates in Class I (low risk) and Class II (high risk) in validation cohort were 97% and 31% respectively.<br><br>Gene expression profile, AJCC stage, Breslow thickness, ulceration, and age were independent predictors of metastatic risk by Cox regression analysis.<br><br>MVA done in subset of 78 of 220 Stage I&II patients (table 3) considering GEP class II, AJCC IIB/C, BD>0.75 mm, Ulceration, Mit rate >1, Age >50 (binarized variables).<br><br>Accuracy 120/134 for class I and 24/30 for class 2 for Stage I and IIA patients.<br><br>Notes:	*Describe conclusions relative to question:*<br><br>GEP signature accurately predicts metastasis risk.<br><br>*Critiques of Methodology:* Relatively few stage I patients (56) and no metastasis in validation cohort of stage I patients although 6 patients classified as class II.<br><br>Subset patient selection for reporting unclear.<br><br>Overlap in discovery, training sets.

				Follow-Up: At least 5 years follow-up or metastatic event—N=34 <5 years follow-up accepted in initial preliminary analysis<br>5 year min or until recurrence; median f/u except 34 patients who had <3 yrs f/u data.<br>---<br>*Notes:*<br>Tumor density on slide>60% required.<br>Signature genes enumerated.		

	Summary of Evidence Table<br>Question 2					
Level of Evidence*^<br><br>Choose one:	Study Citation<br>Author(s), Year, Title, Journal, Volume, Page #s	Funding Source	Study Methodology	Objective & Methods	Results	Conclusions
☐EL 1; RCT<br>☐EL 1; MRCT<br>(Strong)<br><br>☒EL 2; MNRCT<br>☐EL 2; NMA<br>☐EL 2; NRCT<br>☐EL 2; PCS<br>☐EL 2; RCCS<br>☐EL 2; NCCS<br>☐EL 2; CSS<br>☐EL 2; ES<br>☐EL 2; OLES<br>☐EL 2; PHAS<br>(Intermediate)<br><br>☐EL 3; DS<br>☐EL 3; ECON<br>☐EL 3; CCS<br>☐EL 3; SCR<br>☐EL 3; PRECLIN<br>☐EL 3; BR<br>(Weak)<br><br>☐EL 4; NE<br>☐EL 4: O<br>(No)	Greenhaw BN, Covington KR, Kurley SJ, et al. Molecular risk prediction in cutaneous melanoma: A meta-analysis of the 31-gene expression profile prognostic test in 1,479 patients. *J Am Acad Dermatol*. 2020;83(3):745-753. (59)<br><br>*Country:* United States	*Source:*<br>Supported by Castle Biosciences, Inc<br><br>4 authors: employees of Castle Biosciences<br><br>4 authors: options owners at Castle Biosciences<br><br>3 authors: speakers bureau at Castle Biosciences	*Methodology:*<br><br>Meta-analysis of three published studies<br><br>Meta-analysis performed to determine overall effect of 31-gene profile, outcomes for GEP compared to AJCC staging criteria.	*Stated Objective*: To assess consistency of data across studies in the predictive power of the test.<br><br>☐Prospective<br>☒Retrospective<br><br>Study Population and Setting:<br><br>Meta-analysis of 3 studies (out of 51 identified).<br><br>N: 1479 stage I – III patients<br><br>Intervention: Predictive modeling<br>: meta-analysis comparing GEP performance to AJCC<br><br>Outcome Measures: RFS and DMFS<br><br>Follow-Up: media 3.3 years<br><br>____________________<br>Notes:	*Results:*<br>5-year recurrence-free survival rate was 91.4% for Class 1A patients and 43.6% for Class 2B patients (*P* < .0001).<br><br>The 5-year distant metastasis-free survival rate was 94.1% (95% CI 91.9%-96.4%) for Class 1A patients and 55.5% (95% CI 49.9%-61.9%) for Class 2B patients (*P* < .0001).<br><br>Ten-year recurrence-free survival rates for Class 1A and 2B were 88.3%, 38.8% and 10-year distant metastasis-free survival rates were 90.8% and 49.9% (see Table 1 and Table 3).<br><br>____________________<br>Notes:	*Describe conclusions relative to question:*<br><br>GEP consistently differentiates between high risk and low risk patients.<br><br>*Critiques of Methodology:*<br><br>Only 10% of the patients in the analysis are IIB or IIC patients, a total of 156 individuals. This severely impacts the statistical reliability of any analysis of these patients.<br><br>85% of the Stage IA patients were class IA which also likely skews the data.<br><br>This accompanying commentary summarizes significant concerns with methodology:

						Problematic methodology in a systematic review and meta-analysis of DecisionDx-Melanoma Journal of the American Academy of Dermatology, Volume 83, Issue 5, November 2020, Pages e357-e358 Michael A. Marchetti, Stephen W. Dusza, Edmund K. Bartlett<br><br>Search criteria included “31-gene” which is specific for their assay (not unbiased look at GEP in general). Their cohorts were heterogeneous across stages I – III.

	Summary of Evidence Table<br>Question 1, 2					
Level of Evidence*^<br><br>Choose one:	Study Citation<br>Author(s), Year, Title, Journal, Volume, Page #s	Funding Source	Study Methodology	Objective & Methods	Results	Conclusions
Strong<br>☐EL 1; RCT<br>☐EL 1; MRCT<br><br>Intermediate<br>☐EL 2; MNRCT<br>☐EL 2; NMA<br>☐EL 2; NRCT<br>☐EL 2; PCS<br>☒EL 2; RCCS<br>☐EL 2; NCCS<br>☐EL 2; CSS<br>☐EL 2; ES<br>☐EL 2; OLES<br>☐EL 2; PHAS<br><br>Weak<br>☐EL 3; DS<br>☐EL 3; ECON<br>☐EL 3; CCS<br>☐EL 3; SCR<br>☐EL 3; PRECLIN<br>☐EL 3; BR<br><br>No Evidence<br>☐EL 4; NE<br>☐EL 4: O	Greenhaw BN, Zitelli JA, Brodland DG. Estimation of Prognosis in Invasive Cutaneous Melanoma: An Independent Study of the Accuracy of a Gene Expression Profile Test. *Dermatol Surg*. 2018 Dec;44(12):1494-1500. (108)<br><br>*Country:* USA	*Source:*<br>None listed<br><br>The authors have indicated no significant interest with commercial supporters.	*Methodology:*<br>Retrospective cohort study assessing prognostic value of 31-GEP test.<br><br>Melanoma Registry data from two private dermatology practices in MI and PA.<br><br>Retrospective review of all patients in a single center prospective database.<br><br>Retrospective analysis of selected patients in institutional database.<br><br>Retrospective analysis of institutional database of patients undergoing 31-GEP testing supplemented with additional	*Stated Objective:*<br>independent study of predictive accuracy of 31-GEP 4 classes (for recurrence?).<br><br>☒Prospective<br>☐Retrospective<br><br>Retrospective study of prospectively collected registry melanoma database<br><br>*Study Population and Setting*: : Two private dermatology practices 2013-2017 where 31-GEP done routinely once commercially available.<br><br>Single center, referral practice<br><br>*N*: N=256<br><br>*Intervention:* 31-GEP<br>GEP on all patients prospectively<br>31-GEP<br><br>*Outcome Measures:*<br>Melanoma free survival<br>Melanoma specific survival<br>Recurrence<br>Recurrent free survival and patterns<br>Metastasis at mean f/u 23 months	*Results:*<br><br>GEP test accurately predicted 77% of patients as Class II who developed metastasis;<br>Negative predictive value of Class I was 99%;<br>5 yr MFS rates of Class I and II 93% and 69% respectively;<br>5 yr MSS 99% and 79% respectively for Class I and II.<br><br>10 (77%) of the 13 patients who developed metastatic disease accurately identified as Class 2.<br><br>NPV for Class 1 was 99%: 3 of 214 pts classified as Class 1 developed metastatic disease at median f/u 23 months.<br><br>193 Class 1A<br>21 Class 1B<br>16 Class 2A<br>26 Class 2B<br><br>MVA – ulc + Breslow depth (2.0 mm for Class 2 vs 0.6 mm for Class 1) associated with Class 2 status (not age, sex, mitoses).	*Describe conclusions relative to question:*<br><br>31-GEP test helped in prognostication and could potentially be helpful in directing patient care.<br><br>Authors state GEP test performance is helpful to direct patient care.<br><br>The goal of this study was to validate GEP as an alternative to SLNB for prognostication. While not explicitly stated, that becomes evident in the rhetoric. The study simply validates the prognostic value of GEP retrospectively in a cohort of patients. It does not analyze whether it is discerning when compared to traditional histologic staging methodologies.<br><br>Authors conclude 31-GEP is prognostic and can guide care.<br><br>*Critiques of Methodology:*

			cases identified as having metastatic disease whose primary tumors were then retrieved for 31-GEP if not done previously	*Follow-Up:* 23 months<br>Median <2 years<br>Mean 23 months<br><br>*Notes:*<br>Also analyze patient and tumor features associated with 31-GEP class and performance of 1B and 2A categories in terms of clinical utility.<br><br>SLN+ not considered as an event for this study.<br><br>No data on untested pts.	13 patients developed metastases, GEP identified 77% of these.<br><br>99% NPV of the GEP "only 3 of the 214 Class I patients metastasized."<br><br>24 of 37 stage II patients were Class II and 10 metastasized, (not surprising), on 42%.<br><br>2 of 13 stage II that were Class I metastasized.<br><br>Of 256: 214 (84%) Class 1, (193 Class 1A, 21 Class 1B)<br>42 (16%) Class 2, (16 Class 2A, 26 Class 2B)<br><br>Of 256: 13 developed metastasis (10 of 13 Class 2 on testing).<br><br>3-year MFS rate 98%for Class 1 patients and 74% for Class 2 patients, with 5-year MFS rates of 93% and 69%, respectively (p < .00001)<br><br>Notes: No comparison based on histologic staging beyond stage I and Stage II (no substaging comparisons).<br><br>Notes:	Relatively short follow-up.<br>Only 13 metastatic events in the entire cohort making any robust multivariate analysis not possible.<br><br>Unclear what the treatment was of the primary (WLE? SLNB?) and type of recurrence is not stated.<br><br>Short f/u time.<br><br>Mention in Data Collection that separate IRB to retrospectively test tumors from a subcohort of patients with known metastatic disease after initial excision.<br><br>Flaws in study with key treatment and other data elements (AJCC stage, e.g.) missing and very short f/u and >80% of patients studied at very low risk based on conventional pathology risk factors – in the population of interest N is small.<br><br>*Critiques of Methodology:*<br>Quoted from the paper:<br><br>*At the time of initial CM diagnosis, 219 (86%) of the*

						tested tumors were Stage I. None of the 18 Stage I Class 2 tumors metastasized, whereas 1 (0.5%) of 201 Stage I Class 1 tumors metastasized.<br><br>This implies that that the GEP is not discerning in Stage I patients since Class differentiation did not predict any pattern of disease recurrence.<br><br>Discussion tries to explain why their findings might not reflect the ultimate utility of the GEP.<br><br>Retrospective<br><br>Enriched for subsequent testing after recurrence in some of the patients, number of patients in this subcohort nor method for identification not stated<br><br>With median f/u 23 months 5 yr KM survival analysis seems invalid.<br><br>Class 1 median Breslow 0.6 mm vs Class 2 = 2.2 mm<br><br>No analysis comparing value of 31-GEP beyond AJCC stage, Breslow Depth

Summary of Evidence Table<br>Question 1,2,3						
Level of Evidence*^<br><br>Choose one:	Study Citation<br>Author(s), Year, Title, Journal, Volume, Page #s	Funding Source	Study Methodology	Objective & Methods	Results	Conclusions
☐EL 1; RCT<br>☐EL 1; MRCT<br>Strong<br><br>☐EL 2; MNRCT<br>☐EL 2; NMA<br>☐EL 2; NRCT<br>☐EL 2; PCS<br>☐EL 2; RCCS<br>☐EL 2; NCCS<br>☐EL 2; CSS<br>☐EL 2; ES<br>☐EL 2; OLES<br>☐EL 2; PHAS<br>Intermediate<br><br>☐EL 3; DS<br>☐EL 3; ECON<br>☐EL 3; CCS<br>☐EL 3; SCR<br>☐EL 3; PRECLIN<br>☐EL 3; BR<br>Weak<br><br>☒EL 4; NE<br>☐EL 4: O<br>No	Grossman D, Okwundu N, Bartlett EK, et al. Prognostic Gene Expression Profiling in Cutaneous Melanoma: Identifying the Knowledge Gaps and Assessing the Clinical Benefit. *JAMA Dermatol.* 2020;156(9):1004-1011.<br>(61)<br><br>*Country:* United States	*Source:* Supported by the University of Utah Department of Dermatology, the Huntsman Cancer Foundation at the University of Utah, the Melanoma Center at the Huntsman Cancer Institute, the Hope Foundation, the National Health and Medical Research Council of Australia Program, fellowship grants and grants from the American Skin Association and the Sydney Medical School Foundation, This material is the result of	*Methodology:*<br><br>Consensus statement	*Stated Objective:* Develop guidelines within the national Melanoma Prevention Working Group (MPWG) on integration of GEP testing into management of patients with CM.<br>5. Review of published data using GEP tests<br>6. Definition of acceptable performance criteria<br>7. Current recommendations of use of GEP in clinical practice<br>8. Considerations for future studies<br><br>☐Prospective<br>☒Retrospective<br><br>Study Population and Setting:<br><br>N: N/A<br><br>Intervention: Consensus guideline<br><br>MPWG members and other international specialists, 2 surveys	*Results:*<br><br>The MPWG members are optimistic about the future use of prognostic GEP testing to improve risk stratification and enhance clinical decision-making but acknowledge that current utility is limited by test performance in patients with stage I disease. Published studies of GEP testing have not evaluated results in the context of all relevant clinicopathologic factors or as predictors of regional nodal metastasis to replace sentinel lymph node biopsy (SLNB). The performance of GEP tests has generally been reported for small groups of patients representing particular tumor stages or in aggregate form, such that stage-specific performance cannot be ascertained, and without survival outcomes compared with data from the American Joint Committee on Cancer 8th edition melanoma staging system international database. There are significant challenges to	*Describe conclusions relative to question:* Enthusiasm for the concept, but limited test performance in stage I disease. Published studies not evaluated results in context of all relevant clinicopathologic factors or as predictors of regional nodal metastasis to replace SLNB.<br><br>More studies are needed to determine clinical utility of GEP.<br><br>*Critiques of Methodology:*<br><br>Consensus guideline was performed reasonably.

		work supported with resources and use of facilities at the Veterans Affairs Palo Alto Health Care System in Palo Alto CA. Contents do not represent the views of the US Dept of Veterans Affairs or the US Gov't. The funders had no role in the design and conduct of the study; collection, management, analysis and interpretation of data, preparation, review or approval of the manuscript; and decision to submit for publication.<br><br>1 author: nonfinancial support from Castle Biosciences outside submitted work.<br><br>1 author: grants from Castle		Outcome Measures: N/A<br><br>Follow-Up:<br><br>________________________<br>Notes:	performing clinical trials incorporating GEP testing with SLNB and adjuvant therapy. The MPWG members favor conducting retrospective studies that evaluate multiple GEP testing platforms on fully annotated archived samples before embarking on costly prospective studies and recommend avoiding routine use of GEP testing to direct patient management until prospective studies support their clinical utility.<br><br>______________________<br>Notes:	

		Biosciences outside submitted work<br><br>1 author: personal fees outside the submitted work Castle Biosciences<br><br>1 author: personal fees from Neracare outside submitted work<br><br>1 author: manuscripts and abstracts published using the test with company support of the assay—all publications were peer review and no personal of institutional payment or compensation was rec'd. Castle Biosciences<br><br>1 author: served as an investigator for Castle Biosciences (no personal financial compensation)				

	Summary of Evidence Table<br>Question 2					
Level of Evidence*^<br><br>Choose one:	Study Citation<br>Author(s), Year, Title, Journal, Volume, Page #s	Funding Source	Study Methodology	Objective & Methods	Results	Conclusions
☐EL 1; RCT<br>☐EL 1; MRCT<br><br>☐EL 2; MNRCT<br>☐EL 2; NMA<br>☐EL 2; NRCT<br>☒EL 2; PCS<br>☐EL 2; RCCS<br>☐EL 2; NCCS<br>☐EL 2; CSS<br>☐EL 2; ES<br>☐EL 2; OLES<br>☐EL 2; PHAS<br><br>☐EL 3; DS<br>☐EL 3; ECON<br>☐EL 3; CCS<br>☐EL 3; SCR<br>☐EL 3; PRECLIN<br>☐EL 3; BR<br><br>☐EL 4; NE<br>☐EL 4: O	Strong / Intermediate / Weak / No Evidence<br><br>Hsueh EC, DeBloom JR, Lee J, et al. Interim Analysis of Survival in a Prospective, Multi-Center Registry Cohort of Cutaneous Melanoma Tested With a Prognostic 31-Gene Expression Profile Test. *J Hematol & Oncology* 2017;10(1):152.<br><br>*Country:* USA	*Source:*<br><br>Partially sponsored by Castle Biosciences, Inc. which provided financial compensation to those centers contributing melanoma tissue to the study.<br><br>4 authors: employees of Castle Biosciences<br><br>4 authors: hold stock on Castle Biosciences<br><br>2 authors: on Castle Bioscience speakers bureau<br><br>4 authors: rec'd honoraria for advisory board participation	*Methodology:*<br>Used pts from two ongoing GEP studies: EXPAND and INTEGRATE who had GEP testing done; outcomes being followed.<br><br>Used pts/samples from 2 studies (EXPAND/INTEGRATE).<br><br>All had GEP test data, and at least 1 f/u visit.<br><br>Prospective multicenter cohort study	*Stated Objective*:<br>The objective of the reported study was a prospective evaluation of the GEP performance in patients enrolled in two clinical registries.<br><br>☒Prospective<br>☐Retrospective<br><br>*Study Population and Setting:*<br><br>*N:* 322<br>282 staged with SNB<br><br>*Intervention:* All had castle test 31 GEP<br><br>*Outcome Measures:*<br>RFS<br>DMFS<br>OS<br>Survival: RFS/DMFS/OS<br><br>Follow-Up: Median 1.5 yrs<br><br>*Notes:* Really short follow-up for melanoma study	*Results:*<br><br>Median f/u 1.5 yrs<br>Median Breslow 1.2mm<br>88% Stage I/II<br>74% SLN bx<br>77% Class I<br><br>Med Breslow 1.2mm<br>88% Stage I/II disease<br>74% had SLNbx.<br><br>77% class 1<br><br>Multivariate Cox regression showed Breslow thickness, mitotic rate, and GEP class to significantly predict recurrence ($p < 0.01$), while tumor thickness was the only significant predictor of distant metastasis and overall survival in this interim analysis.<br><br>74/322 class II (23%)<br>GEP classification significantly associated with RFS /DMFS and OS on univariate analysis.<br>Class II heavily correlated with ulceration and Breslow thickness.<br>Class 2 not independent predictor of DMFS or OS.<br>Cox shows class 2 independent predictor of	*Describe conclusions relative to question:*<br><br>Interim analysis supports 31-GEP ability to stratify early stage mel pts into two groups.<br><br>GEP testing "complements" current clinicopathologic features.<br><br>Interim analysis of patient outcomes from a combined prospective cohort supports the 31-gene GEP's ability to stratify early-stage CM patients into two groups with significantly different metastatic risk.<br><br>Class 2 associated with worse prognosis on this very limited follow-up study.<br><br>Interim data so no clinical

					recurrence-free survival, along with Breslow, mitotic rate and sentinel node status.<br><br>Ulceration status not significant independent predictor of RFS, suggesting class 2 is surrogate marker of ulceration (which in turn would be called “poorly differentiated” if it were any other tumor)<br><br>*Notes*:	recommendations could be made.<br><br>*Critiques of Methodology:*<br>-Short f/u < than 2 years overall will not capture all recurrence/survival events.<br><br>No new evidence from this alone.<br><br>Short F/u < 2 years.

	Summary of Evidence Table<br>Question 2					
Level of Evidence*^<br><br>Choose one:	Study Citation<br>Author(s), Year, Title, Journal, Volume, Page #s	Funding Source	Study Methodology	Objective & Methods	Results	Conclusions
☐EL 1; RCT<br>☐EL 1; MRCT<br><br>☐EL 2; MNRCT<br>☐EL 2; NMA<br>☐EL 2; NRCT<br>☒EL 2; PCS<br>☐EL 2; RCCS<br>☐EL 2; NCCS<br>☐EL 2; CSS<br>☐EL 2; ES<br>☐EL 2; OLES<br>☐EL 2; PHAS<br><br>☐EL 3; DS<br>☐EL 3; ECON<br>☐EL 3; CCS<br>☐EL 3; SCR<br>☐EL 3; PRECLIN<br>☐EL 3; BR<br><br>☐EL 4; NE<br>☐EL 4: O<br><br>Strong | Intermediate | Weak | No Evidence	Hsueh EC, DeBloom JR, Lee JH, et al. Long-Term Outcomes in a Multicenter, Prospective Cohort Evaluating the Prognostic 31-Gene Expression Profile for Cutaneous Melanoma. *JCO Precis Oncol.* Apr 6, 2021;5:<br><br>*Country:* USA	*Source:*<br>This study was partially sponsored by Castle Biosciences, which provided financial compensation to those who provided melanoma tissue for this study.<br><br>4 authors: employees of Castle Biosciences<br><br>4 author: stock and other ownership interests<br><br>1 author: Castle Biosciences speakers bureau<br><br>1 author: Consulting or advisory role with Castle Biosciences<br><br>2 author: patent, royalty, other intellectual property in GEP tests or Castle	*Methodology:*<br>Used INTEGRATE/EXPAND Patients with Stage I-III CM.<br><br>Multicenter registry INTEGRATE and EXPAND<br>RFS, DMFS, OS assessed using Kaplan-Meier and Cox regression analysis.	*Stated Objective:*<br>To test the hypotheses that the 31-GEP provides prognostic value for patients with stage I-III CM, and that patients with stage I-IIA melanoma and class 2 31-GEP results have metastatic risk similar to patients for whom surveillance is recommended.<br><br>To assess if the 31-GEP add prognostic value to clinicopathologic features.<br><br>☒Prospective<br>☐Retrospective<br><br>*Study Population and Setting:*<br>Stage I- III CM<br><br>11 centers<br>CM stage I-III pts 16 years old with GEP results<br><br>*N:* 372 assessed for eligibility. 334 met enrollment criteria and had test results were enrolled. Eleven were excluded from analysis, leaving 323 who met enrollment and analysis inclusion criteria.<br><br>*Intervention:* All had castle 31-GEP test	*Results:*<br>The 31-GEP was significant for RFS, DMFS, and OS in a univariate analysis and was a significant, independent predictor of RFS, DMFS, and OS in a multivariable analysis.<br><br>GEP class 2 results were significantly associated with lower 3-year RFS, DMFS, and OS in all patients and those with stage I-IIA disease.<br><br>Patients with stage I-IIA CM and a class 2 result had recurrence, distant metastasis, and death rates similar to patients with stage IIB-III CM.<br><br>Class 2 were associated with high-risk CP features as compared to class 1 including age, male, Breslow, ulceration and + SLN.<br><br>Class 2 v Class 1: 3 -year<br>RFS 66% v 95%<br>DMFS 79% v 97%<br>OS 81% v 95%<br><br>univariate analysis including class 2 (v class 1) 31-	*Describe conclusions relative to question:*<br>Pts with Stage I-IIA CM and class 2 GEP may be candidates for more intense f/u.<br><br>31-GEP is a significant, independent prognostic factor for 3-year recurrence-free survival, distant metastasis-free survival, and overall survival in a prospectively enrolled cohort of patients diagnosed with stage I-III melanoma. Patients diagnosed with stage I-IIA melanoma who receive a high-risk 31-GEP result (class 2) have

		Bioscience related patents<br><br>4 author: travel, accommodations, expenses, Castle Biosciences		*Outcome Measures:* RFS, DMFS, OS<br><br>*Follow-Up*: 3.2 years<br><br>*Notes:*	GEP results, continuous age, male (v female) sex, head and neck (v non–head and neck) tumor location, continuous mitotic rate, and high-risk stage IIB-III (v stage I-IIA) AJCC 8th ed staging, only GEP class 2 and high-risk AJCC stages reached a threshold of P , .01 for<br><br>all three end points; whereas, mitotic rate was signifi- cant for DMFS, and age was significant for OS.<br><br>Similarly, subclass analysis of the 31-GEP demonstrated that classes 1B, 2A, and 2B were significant<br><br>predictors of recurrence risk; whereas, classes 2A and 2B were significant predictors of distant metastasis, and class 2B was a significant predictor of mortality<br><br>In a multivariable analysis, 31-GEP class 2 (hazard ratio [HR], 4.34 [95% CI, 2.10 to 8.96], P , .001) and AJCC stage IIB-III<br><br>(HR, 2.98 [95% CI, 1.48 to 6.02], P = .002) were inde-	survival outcomes like those with stage IIB-III melanoma, for whom national guidelines recommend more intense follow-up.<br><br>*Critiques of Methodology:* Relatively short f/u<br><br>Doesn't break down by stage

					pendent, significant predictors of RFS. For 3-year DMFS, 31-<br><br>GEP class 2 (HR, 5.45 [95% CI, 2.09 to 14.25], P , .001),<br>AJCC stage IIB-III (HR, 2.81 [95% CI, 1.16 to 6.58], P = .023),<br>and mitotic rate (HR, 1.04 [95% CI, 1.01 to 1.07], P = .007)<br>were significant. 31-GEP class 2 (HR, 3.13 [95% CI, 1.23 to 7.96], P = .016),<br>AJCC stage IIB-III (HR, 3.89 [95% CI, 1.60 to 9.50], P = .003), and age (HR, 1.08 [95% CI, 1.04 to 1.13],<br>P , .001) were significant for OS<br><br>identifying high-risk patients by either 31-GEP class 2 result or AJCC high-risk category, sensitivity was<br>enhanced for 3-year RFS (76%), DMFS (88%), and OS<br>(76%) compared with AJCC alone with sensitivities of 57%<br>(RFS), 62% (DMFS), and 60% (OS) or 31-GEP status<br>alone with sensitivities of 64% (RFS), 69% (DMFS), and<br>68% (OS). Class 2 31-GEP results identified AJCC stage<br>I-IIA patients with increased risk for recurrence, distant	

					metastasis, and death with 44%, 70%, and 40% sensi- tivity, respectively; whereas a class 1 result confirmed a low risk of recurrence, distant metastasis, and death in this population with an NPV of 92%, 98%, and 95%, respectively<br>*Notes*: multivariate HR for OS almost identical	

	Summary of Evidence Table<br>Question 1, 2					
Level of Evidence*^<br><br>Choose one:	Study Citation<br>Author(s), Year, Title, Journal, Volume, Page #s	Funding Source	Study Methodology	Objective & Methods	Results	Conclusions
**Strong**<br>☐EL 1; RCT<br>☐EL 1; MRCT<br><br>**Intermediate**<br>☐EL 2; MNRCT<br>☐EL 2; NMA<br>☐EL 2; NRCT<br>☐EL 2; PCS<br>☐EL 2; RCCS<br>☐EL 2; NCCS<br>☐EL 2; CSS<br>☐EL 2; ES<br>☐EL 2; OLES<br>☒EL 2; PHAS<br><br>**Weak**<br>☐EL 3; DS<br>☐EL 3; ECON<br>☐EL 3; CCS<br>☐EL 3; SCR<br>☐EL 3; PRECLIN<br>☐EL 3; BR<br><br>**No Evidence**<br>☐EL 4; NE<br>☐EL 4: O	Jarell A, Gastman BR, Dillon LD, et al. Optimizing Treatment Approaches for Patients With Cutaneous Melanoma by Integrating Clinical and Pathologic Features with the 31-Gene Expression Profile Test. *J Am Acad Dermtol*.. 2022. 87(6):<br>(85): 1312-1320.<br><br>*Country:* USA	*Source:*<br>Funded by Castle Biosciences, Inc.<br><br>8 authors: Castle Bioscience employees<br><br>8 authors: stock and options holders for Castle Biosciences<br><br>2 authors: speakers bureau for Castle Biosciences	*Methodology:*<br>Algorithm development.<br><br>Analysis of existing datasets.<br><br>"The cohort comprised 2104 patients from four independent centers (n = 548) and a previously published meta-analysis that combined two retrospectively tested (n = 990) and two prospectively tested (n = 566) cohorts.	*Stated Objective:* Risk of recurrence.<br><br>"To develop an algorithm by integrating the 31-gene expression profile test with clinicopathologic data for an optimized, personalized risk of recurrence (integrated 31 risk of recurrence [i31-ROR]) or death and use i31-ROR in conjunction with a previously validated algorithm for precise sentinel lymph node positivity risk estimates (i31-SLNB) for optimized treatment plan decisions."<br><br>☐Prospective<br>☒Retrospective<br><br>*Study Population and Setting*:<br>Multicenter cohort<br>See methodology<br><br>*N:* 1581 development cohort; 523 validation cohort<br><br>Intervention:<br>31-GEP<br>AI<br><br>*Outcome Measures:*<br>Recurrence as measured by a personalized risk of recurrence (integrated 31 risk of recurrence (i31-ROR)).<br><br>RFS, DMFS, Mel Sp Survival	*Results:*<br>I31-ROR risk (low vs high) may give personalized prediction of recurrence (RFS, DMFS, MSS) risk and SLNB results.<br><br>Mathematical modeling including GEP improved prediction of RFS, MSS and DMFS. Also analyzed SLN vs model for outcomes.<br><br>*Notes:*	*Describe conclusions relative to question:*<br><br>Algorithm incorporating GEP may give personalized prediction of recurrence risk and SLNB results.<br><br>Patient risk can be analyzed more comprehensively, resulting in better patient care. We further showed that the number of patients undergoing SLNB could be reduced,"<br><br>Mathematical modeling with no clinical testing.<br><br>*Critiques of Methodology*<br><br>SLNB was only taken into account post hoc.<br>:

				Follow-Up: n/a<br>unclear<br>*Notes:*<br>Could be considered retrospective cohort study vs. data science.		

	Summary of Evidence Table<br>Question 2					
Level of Evidence*^<br><br>Choose one:	Study Citation<br>Author(s), Year, Title, Journal, Volume, Page #s	Funding Source	Study Methodology	Objective & Methods	Results	Conclusions
**Strong**<br>☐EL 1; RCT<br>☐EL 1; MRCT<br><br>**Intermediate**<br>☐EL 2; MNRCT<br>☐EL 2; NMA<br>☐EL 2; NRCT<br>☐EL 2; PCS<br>☒EL 2; RCCS<br>☐EL 2; NCCS<br>☐EL 2; CSS<br>☐EL 2; ES<br>☐EL 2; OLES<br>☐EL 2; PHAS<br><br>**Weak**<br>☐EL 3; DS<br>☐EL 3; ECON<br>☐EL 3; CCS<br>☐EL 3; SCR<br>☐EL 3; PRECLIN<br>☐EL 3; BR<br><br>**No Evidence**<br>☐EL 4; NE<br>☐EL 4: O	Jarell A, Skenderis B, Dillon LD, et al. The 31-Gene Expression Profile Stratifies Recurrence and Metastasis Risk in Patients with Cutaneous Melanoma. *Future Oncol.* 2021;17(36):5023-5031. (73)<br><br>*Country:* USA	*Source:*<br>Funded by Castle Biosciences, Inc.<br><br>7 authors: employees of Castle Biosciences<br><br>7 authors: Castle Bioscience stock holders<br><br>2 authors: speakers bureau for Castle Biosciences	*Methodology:*<br>Retrospective cohort study	*Stated Objective:*<br>Assess outcomes in Class I vs Class II.<br><br>Assess RFS, DMFS, MSS (melanoma -specific survival)l-risk stratify and effect of adding AJCC.<br><br>☐Prospective<br>☒Retrospective<br><br>Study Population and Setting:<br>Multi-site practices<br>Derm clinic and surgeons<br><br>N: 438<br><br>Intervention:<br>31-GEP assay<br><br>Outcome Measures: RFS, DMFS, MSS<br><br>Follow-Up: between 1.27 and 1.69 years (depending on group).<br><br>Class 1A 1.27 yrs<br>Class 1B/2A 1.68 yrs<br>Class 2B 1.56 yrs<br><br>*Notes:*	*Results:* Class 2B has higher risk of recurrence than Class 1A.<br><br>"The 31-GEP significantly stratified patient risk for recurrence-free survival (p<0.001), distant metastasis-free survival (p<0.001) and melanoma-specific survival (p<0.001) and was a significant, independent predictor of metastatic recurrence (hazard ratio: 5.38; p=0.014)."<br><br>*Notes:* Not all patients underwent SLNB	*Describe conclusions relative to question:*<br><br>Class 2B has higher risk of recurrence than Class 1A.<br><br>Supported<br><br>*Critiques of Methodology:*<br>Not all patients underwent SLNB, unclear if this was a consecutive group of patients so possibility of selection bias.<br><br>Small series<br><br>Short follow up<br><br>Retrospective<br><br>Few stage III<br><br>Low event rate

Level of Evidence*^ Choose one:	Summary of Evidence Table Question 2					
	**Study Citation** Author(s), Year, Title, Journal, Volume, Page #s	**Funding Source**	**Study Methodology**	**Objective & Methods**	**Results**	**Conclusions**
Strong: ☐EL 1; RCT<br>☐EL 1; MRCT<br><br>Intermediate: ☐EL 2; MNRCT<br>☐EL 2; NMA<br>☐EL 2; NRCT<br>☐EL 2; PCS<br>☒EL 2; RCCS<br>☐EL 2; NCCS<br>☐EL 2; CSS<br>☐EL 2; ES<br>☐EL 2; OLES<br>☐EL 2; PHAS<br><br>Weak: ☐EL 3; DS<br>☐EL 3; ECON<br>☐EL 3; CCS<br>☐EL 3; SCR<br>☐EL 3; PRECLIN<br>☐EL 3; BR<br><br>No Evidence: ☐EL 4; NE<br>☐EL 4: O	Kangas-Dick AW, Greenbaum A, Gall V, et al. Evaluation of a Gene Expression Profiling Assay in Primary Cutaneous Melanoma.*Ann Surg Oncol*. 2021;28(8):4582-4589<br>(74)<br><br>*Country:* USA<br><br>POST PUBLICATION DISCUSSION: In Reply<br><br>Reply AnnSurgOnc 29:818, 2022	*Source:* none<br><br>Not stated	*Methodology:* Castle GEP<br><br>Retrospective review	*Stated Objective:* Prediction of melanoma recurrence.<br><br>Determine whether GEP added to pathologic staging.<br><br>☐Prospective<br>☒Retrospective<br><br>*Study Population and Setting:*<br>Single institution study University hospital<br><br>*N:* 361 initially<br><br>*Intervention:* GEP<br>GEP testing and review<br><br>*Outcome Measures:* RFS, DMFS<br><br>*Follow-Up:*<br>15.3 mos<br><br>Both listed in manuscript.<br>___<br>*Notes:*	*Results:*<br>GEP not predictive in multivariate analysis.<br><br>"The 3- and 5-year RFS rates were respectively 85% and 75% for the class 1A group, 74% and 47% for the class 1B/class 2A group, and54% and 45% for the class 2B group. Increased Breslow thickness, ulceration, mitoses, sentinel node biopsy positivity, and GEP class 2B status were significantly associated with RFS and distant metastasis-free survival(DMFS) in the univariate analysis (all p<0.05). In the multivariate analysis, only Breslow thickness and ulceration were associated with RFS (p<0.003), and only Breslow thickness was associated with DMFS (p\0.001)."<br><br>Notes: revisions made after letter from editors Drs Roh and Tyler Ann Surg Onc 29:820 2022<br><br>After re-review:" The only factors that were predictive of a recurrence on multivariate analysis were ulceration and SLN biopsy (SLNB) status, which is slightly different from the manuscript, where both Breslow depth and ulceration were predictive, but not SLNB status."___	*Describe conclusions relative to question:*<br><br>Though GEP showed some predictive value on univariate analysis, GEP class was not predictive in multivariate analysis.<br><br>Note revisions.<br><br>*Critiques of Methodology:*<br><br>Not all patients had SLNB; Relatively short f/u time.<br><br>Retrospective<br><br>Limited series with some patients tested ad hoc and/or retrospectively.<br><br>Selection bias<br><br>See ref under results and citation

					Notes: 25% of patients did not have SLNB.	

	Summary of Evidence Table<br>Question 1, 2					
Level of Evidence*^<br><br>Choose one:	Study Citation<br>Author(s), Year, Title, Journal, Volume, Page #s	Funding Source	Study Methodology	Objective & Methods	Results	Conclusions
**Strong**<br>☐EL 1; RCT<br>☐EL 1; MRCT<br><br>**Intermediate**<br>☐EL 2; MNRCT<br>☐EL 2; NMA<br>☐EL 2; NRCT<br>☐EL 2; PCS<br>☐EL 2; RCCS<br>☐EL 2; NCCS<br>☐EL 2; CSS<br>☐EL 2; ES<br>☐EL 2; OLES<br>☐EL 2; PHAS<br><br>**Weak**<br>☐EL 3; DS<br>☐EL 3; ECON<br>☐EL 3; CCS<br>☐EL 3; SCR<br>☐EL 3; PRECLIN<br>☐EL 3; BR<br><br>**No Evidence**<br>☒EL 4; NE<br>☐EL 4: O	Kashani-Sabet M, Leachman SA, Stein JA, et al. Early Detection And Prognostic Assessment Of Cutaneous Melanoma: Consensus On Optimal Practice And The Role Of Gene Expression Profile Testing. *JAMA Dermatol.* 2023;159(5):545-553.<br><br>*Country:* USA	*Source:*<br><br>The funding for the administration and facilitation of the consensus development conference and the development of this manuscript was provided by Dermtech Inc in an unrestricted award that was administratively overseen by the Melanoma Research Foundation and managed and executed at UPMC by the principal investigator (Dr Kirkwood). DermTech was not involved in the preparation or editing of the manuscript.<br><br>The funding organizations had no role in the design and conduct of the	*Methodology:* Modified Delphi consensus used for case scenarios asked of 60 melanoma panelists followed by a consensus conference (n=51)	*Stated Objective:* "To provide consensus recommendations on optimal screening practices and prebiopsy diagnostic, postbiopsy diagnostic, and prognostic assessment of cutaneous melanoma."<br><br>☐Prospective<br>☐Retrospective<br><br>*Study Population and Setting:* consensus conference<br><br>*N*: 60 melanoma panelists, 42 survey respondents, 51 panelists at consensus conference<br><br>*Intervention:* Modified Delphi consensus<br><br>*Outcome Measures:* N/A<br><br>*Follow-Up:*<br><br>*Notes:*	*Results:*<br><br>Consensus (>70% agreement) low-risk GEP score should not outweigh histologic features when determining eligibility for SLNB.<br><br>No consensus on imaging recommendations in the setting of high-risk prognostic GEP score and low-risk histology and/or negative SLN.<br><br><10% of experts recommended post biopsy GEP in management.<br><br>*Notes*: Cite need for additional studies	*Describe conclusions relative to question:*<br><br>No consensus on role of GEP testing in clinical decision making.<br><br>"1 area in which consensus was reached among the panel was that the31-Gene prognostic GEP testing result alone would not outweigh routine histopathologic features to inform selection of patients for SLNB. Panelists supported an approach that favors histopathologic vs GEP testing for SLNB. As shown in Table 4, the panelists consistently recommended WLE plus SLNB for individuals who meet histopathologic criteria for SLNB, even in the setting of a low-risk (class 1) 31-gene GEP testing result. Panelists were not queried regarding various SLNB risk calculators/ nomograms or other GEP testing (ie, Merlin Assay 8-GEP; DecisionDx

		study; collection, management, analysis, and interpretation fo the data; preparation, review, or approval of the manuscript and decision to submit the manuscript for publication.<br><br>2 authors: research support from Castle Biosciences outside this project<br><br>1 author: research support from Castle both during and outside this work<br><br>1 author: grant support from Skyline Dx outside this work<br><br>1 author: nonfinancial support from SkylineDx outside the work				i-31-GEP) purported to predict SLN positivity."<br><br>*Critiques of Methodology:*<br><br>Other than funding support, no concerns. Appropriately designed and performed consensus conference.

	Summary of Evidence Table<br>Question 2					
Level of Evidence*^<br><br>Choose one:	Study Citation<br>Author(s), Year, Title, Journal, Volume, Page #s	Funding Source	Study Methodology	Objective & Methods	Results	Conclusions
Strong<br>☐EL 1; RCT<br>☐EL 1; MRCT<br><br>Intermediate<br>☐EL 2; MNRCT<br>☐EL 2; NMA<br>☐EL 2; NRCT<br>☒EL 2; PCS<br>☐EL 2; RCCS<br>☐EL 2; NCCS<br>☐EL 2; CSS<br>☐EL 2; ES<br>☐EL 2; OLES<br>☐EL 2; PHAS<br><br>Weak<br>☐EL 3; DS<br>☐EL 3; ECON<br>☐EL 3; CCS<br>☐EL 3; SCR<br>☐EL 3; PRECLIN<br>☐EL 3; BR<br><br>No Evidence<br>☐EL 4; NE<br>☐EL 4: O	Keller J, Schwartz TL, Lizalek JM, et al. Prospective Validation of the Prognostic 31-Gene Expression Profiling Test in Primary Cutaneous Melanoma. *Cancer Med*. 2019;8(5):2205-2212. (42)<br><br>*Country*: USA	*Source:*<br>St. Louis University Cancer Center<br><br>Editorial support and statistical assistance from Castle Bioscience<br><br>1 author: speaker bureau for Castle Bioscience	*Methodology:*<br><br>Prospective analysis.<br><br>CM pt undergoing SLNB and GEP testing were classified as low-risk or high-risk. RFS and DMFS were estimated using Kaplan-Meier method.	*Stated Objective:*<br>Evaluated the prognostic utility of 31-GEP test in CM pt undergoing SLNB.<br><br>To determine the predictive value of the GEP-31 profile system.<br><br>☒Prospective<br>☐Retrospective<br><br>*Study Population and Setting:*<br>CM pt diagnosed 1/2013-8/2015 undergoing SNLB.<br><br>Adult patients with melanoma staged with SNB at a single academic cancer centre.<br><br>N: 159<br>(174 with 15 insufficient tumor for GEP= 159)<br><br>*Intervention:*<br>GEP testing &<br>Sentinel node biopsy staging<br><br>*Outcome Measures:*<br>RFS and DMFS survival outcomes<br><br>*Follow-Up*: Median 44.9 month<br><br>*Notes:*	*Results:*<br><br>Class 2 associated with high-risk melanomas and worse outcome.<br><br>Cox's regression shows Breslow, GEP class 2 and SN status both as independent predictor of outcomes.<br><br>Median breslow thickness was 1.4 mm (0.2-15.0 mm). Class 1: 117 pt Class 2: 42 pt.<br><br>Breslow thickness, ulceration, SLNB, AJCC stage significantly associated GEP class ($p<0.05$).<br><br>Class 1 RFS 5% and DMFS 1%.<br><br>Class 2 RFS 55% and DMFS 36%.<br><br>Sensitivity Class 2 79% and SLNB 34%.<br><br>-SLNB +/ Class 2 pt 9/10 recurred.<br>-9/11 (82%) first recurrence stage III/GEP Class 2 were to distant visceral sites.	*Describe conclusions relative to question*:<br><br>Class 2 and SLNB positivity independently associated with recurrence and distant metastasis in primary CM. GEP may have additive prognostic utility in initial staging and work-up.<br><br>GEP highlighting otherwise high-risk tumors. Similar results to other studies above.<br><br>Surrogate marker for poorly differentiated primary.<br><br>*Critiques of Methodology:*<br>Small cohort especially when divided into subclasses. Only 42 patients in Class 2.

					Univariate analysis Breslow thickness, ulceration, SLNB and GEP significantly associated with RFS and DMFS (p<0.001).<br><br>Multivariate analysis only SLNB and GEP class were statistically associated with both RFS (p=0.008 and 0.0001) and DMFS (p=0.0019 and 0.001).<br><br>HR Class 2 9.2 (p<0.001 CI 3.0-28.5) for RFS and 19 (p<0.001 CI 2.12-170.5).<br><br>SLNB result was also associated with RFS and DMFS (P<0.02, HR=3.5, 3.7, 95% CI=1.4-9.1,1.2-11.3).<br><br>Breslow significant for RFS HR 1.15 (p=0.0015, CI 1.01-1.31) not significant for DMFS in multivariate.<br><br>Ulceration not significant.<br><br>GEP subclass additional stratification w 3-yr RFS and DMFS rates for subclass 2B of 39.5% and 59.6%.<br><br>*Notes:*	Investigators not blinded to GEP results.

	Summary of Evidence Table<br>Question 1, 2					
Level of Evidence*^<br><br>Choose one:	Study Citation<br>Author(s), Year, Title, Journal, Volume, Page #s	Funding Source	Study Methodology	Objective & Methods	Results	Conclusions
Strong<br>☐EL 1; RCT<br>☐EL 1; MRCT<br><br>Intermediate<br>☒EL 2; MNRCT<br>☐EL 2; NMA<br>☐EL 2; NRCT<br>☐EL 2; PCS<br>☐EL 2; RCCS<br>☐EL 2; NCCS<br>☐EL 2; CSS<br>☐EL 2; ES<br>☐EL 2; OLES<br>☐EL 2; PHAS<br><br>Weak<br>☐EL 3; DS<br>☐EL 3; ECON<br>☐EL 3; CCS<br>☐EL 3; SCR<br>☐EL 3; PRECLIN<br>☐EL 3; BR<br><br>No Evidence<br>☐EL 4; NE<br>☐EL 4: O	Litchman GH, Prado G, Teplitz RW, & Rigel D (2020). A Systematic Review and Meta-Analysis of Gene Expression Profiling for Primary Cutaneous Melanoma Prognosis. *SKIN The Journal of Cutaneous Medicine.* 4(3): 221–237.<br><br>*Country:* USA	*Source:* None<br><br>1 author: consultant for Castle Biosciences<br><br>2 authors: are fellows of the National Society for Cutaneous Medicine which received grants from Castle Biosciences	*Methodology:* Meta-analysis of six Decision DX studies and systematic review of other GEP.<br><br>Systematic review and meta-analysis.	*Stated Objective:*<br><br>☐Prospective<br>☒Retrospective Meta-analysis/systematic review<br><br>*Study Population and Setting:*<br><br>*N:* 29 studies with 8 gene signatures; 6 with 31-gene signature—meta-analysis performed on the 6.<br><br>*Intervention:*<br><br>Outcome Measures: Prognostic validity, analytic validity and clinical impact of GEP.<br><br>*Follow-Up:*<br><br>*Notes:* 29 articles from systematic review (9 unique gene signatures);<br>6 articles used for the meta-analysis (limited to 1 GEP [31-gene signature])	*Results:* In meta-analysis of 31-gene signature, pooled OR for recurrence was 9.42 and for distant metastases 7.93; pooled OR for OS was 6.43 and for SLN positivity 2.99.<br><br>Pooled OR for SLNB positivity = 2.99 (95% CI: 2.15-4.15).<br><br>Performance with other outcomes was slightly better (recurrence, distant metastases, overall survival).<br><br>*Notes*:	*Describe conclusions relative to question:*<br><br>Results of the study may aid clinicians in using GEP assays and in managing patients with melanoma.<br><br>Pooled OR for SLNB positivity = 2.99 (95% CI: 2.15-4.15).<br><br>Limited meta-analysis (reflecting heterogeneous data).<br><br>*Critiques of Methodology:*<br><br>Significant heterogeneity in studies included for systematic review and meta-analysis.<br><br>Limited meta-analysis (reflecting heterogeneous

						data from 4 fairly dated studies).

	Summary of Evidence Table<br>Question 2					
Level of Evidence*^<br><br>Choose one:	Study Citation<br>Author(s), Year, Title, Journal, Volume, Page #s	Funding Source	Study Methodology	Objective & Methods	Results	Conclusions
Strong<br>☐EL 1; RCT<br>☐EL 1; MRCT<br><br>Intermediate<br>☒EL 2; MNRCT<br>☐EL 2; NMA<br>☐EL 2; NRCT<br>☐EL 2; PCS<br>☐EL 2; RCCS<br>☐EL 2; NCCS<br>☐EL 2; CSS<br>☐EL 2; ES<br>☐EL 2; OLES<br>☐EL 2; PHAS<br><br>Weak<br>☐EL 3; DS<br>☐EL 3; ECON<br>☐EL 3; CCS<br>☐EL 3; SCR<br>☐EL 3; PRECLIN<br>☐EL 3; BR<br><br>No Evidence<br>☐EL 4; NE<br>☐EL 4: O	Marchetti M, Coit D, Dusza S, et al. Performance of Gene Expression Profile Tests for Prognosis in Patients With Localized Cutaneous Melanoma: A Systematic Review and Meta-analysis. JAMA Dermatol. 2020 Sep 1;156(9):953-962.<br>(98)<br><br>*Country:* USA + international	*Source:*<br>Funded in part by Memorial Sloan Kettering Cancer Center institutional National Institutes of Health/ National Cancer Center Support Grant P30 CA008748<br><br>The funding source had no role in the design and conduct of the study; collection, management, analysis and interpretation of the data; preparation, review, or approval of the manuscript; and decision to submit the manuscript for publication.	*Methodology:*<br>Meta-analysis/systematic review.<br><br>Systematic review/meta-analysis<br><br>Adaptation of the Checklist for Critical Appraisal and Data Extraction for Systematic Reviews of Prediction Modeling Studies (CHARMS-PF)<br><br>Literature Review of 2015-2019 articles indexed in PubMed plus relevant ASCO 2017-2019 abstracts reviewed by 2 rounds on line survey and in-person 2 hours meeting with review of survey results, relevant articles.	*Stated Objective:*<br>Performance of commercially available GEP tests in predicting outcomes in patients with stage I/II melanoma.<br><br>☒Prospective<br>☒Retrospective<br>Systematic review and Meta-analysis included retrospective and retrospective studies<br><br>*Study Population and Setting:*<br><br>7 studies (5 DecisionDx; 2 MelaGEnix) 1450 study participants<br><br>Meta-analysis and expert consensus by Melanoma Prevention Working Group + other international melanoma specialists (interdisciplinary including patient advocates).<br><br>*N*: 1450 patients<br><br>*Intervention:*<br>GEP testing<br><br>*Outcome Measures:*<br>Melanoma recurrence Proportion of patients with melanoma recurrence accurately predicted by GEP Test.	*Results:*<br><br>Among patients with recurrence Decision Dx correctly classified 29% and 82% with stage I and II disease respectively.<br><br>Among patients with recurrence MelaGenix correctly classified 32% and 76% with stage I and II disease respectively.<br><br>Without recurrence.<br><br>Decision Dx 90% and 44% with stage I and II disease Respectively.<br><br>MelaGenix 77% and 43% with stage I and II disease respectively.<br><br>Before GEP testing is routinely used, the clinical benefit must be established through further clinical investigations.<br><br>For Decisions Dx:<br>In patients with recurrence, accurately predicted recurrence in 29% stage I and 82% stage II;	*Describe conclusions relative to question:*<br><br>GEP testing poor in predicting recurrence in patients with Stage I disease.<br><br>GEP assays appear to have poor predictive capacity for patients with stage I melanoma.<br><br>Prognostic ability was felt to be poor for stage I disease for both GEPs evaluated.<br><br>Prognosticati on was better in stage II disease<br><br>*Critiques of Methodology:*<br><br>Heterogeneity in study

				Follow-Up:<br><br>*Notes:* 7 studies (including 2 GEPs)	In patients without recurrence, accurately predicted this outcome in 90% and 44% for stage I and II respectively.<br><br>For Melagenix:<br>In patients with recurrence, accurately predicted recurrence in 32% and 76% for stage I and II respectively; In patients without recurrence, accurately predicted this outcome in 77% and 43% for stage I and II respectively.<br><br>Primary endpoint was whether GEP correctly classified risk of recurrence.<br><br>Overall, prognostic ability was felt to be poor for stage I disease for both GEPs evaluated.<br><br>Among patients with recurrence, DecisionDx-Melanoma correctly classified 29% with stage I disease and 82% with stage II disease. Among patients without recurrence, the test correctly classified 90% with stage I disease and 44% with stage II disease.<br><br>Among patients with recurrence, MelaGenix correctly classified 32% with stage I disease and 76%with stage II disease. Among patients	designs included and follow-up and reporting.<br><br>Relatively short follow-up of several studies;<br><br>Quality of evidence assessment of studies included could be subject to biases despite use of structured tools.<br><br>Heterogeneou s studies of varying quality and of varying length of patient f/u.<br><br>Meta-analysis of HRs was not able to be done. Potential overlap of cohorts in studies.

					without recurrence, the test<br>correctly classified 77% with stage I disease and 43% with stage II disease.<br><br>*Notes:*	

	Summary of Evidence Table<br>Question 2					
Level of Evidence*^<br><br>Choose one:	Study Citation<br>Author(s), Year, Title, Journal, Volume, Page #s	Funding Source	Study Methodology	Objective & Methods	Results	Conclusions
☐EL 1; RCT<br>☐EL 1; MRCT<br>Strong<br><br>☐EL 2; MNRCT<br>☐EL 2; NMA<br>☐EL 2; NRCT<br>☐EL 2; PCS<br>☒EL 2; RCCS<br>☐EL 2; NCCS<br>☐EL 2; CSS<br>☐EL 2; ES<br>☐EL 2; OLES<br>☐EL 2; PHAS<br>Intermediate<br><br>☐EL 3; DS<br>☐EL 3; ECON<br>☐EL 3; CCS<br>☐EL 3; SCR<br>☐EL 3; PRECLIN<br>☐EL 3; BR<br>Weak<br><br>☐EL 4; NE<br>☐EL 4: O<br>No Evidence	Marks E, Caruso HG, Kurley SJ, Ibad S, et al. Establishing an evidence-based decision point for clinical use of the 31-gene expression profile test in cutaneous melanoma. *SKIN The Journal of Cutaneous Medicine*. (2019); 3(4): 239–249.<br><br>*Country:* USA-single center	*Source:* None<br><br>4 authors: employees Castle Biosciences<br><br>4 authors: options holders at Castle Biosciences<br><br>1 author: has served as a consultant Castle Biosciences<br><br>1 author: has served as a speaker Castle Biosciences	*Methodology:* Retrospective case series	*Stated Objective:* An evidence-based analysis of a decision point for use in thin, T1 tumors would be clinically useful.<br><br>☐Prospective<br>☒Retrospective<br><br>Study Population and Setting: we evaluated changes in patient management, cumulative differential risk across Breslow thicknesses based on a large dataset, and 31-GEP subclass distribution in a clinically tested cohort<br><br>Academic center-Texas<br><br>N: 1479<br>571-T1 melanoma<br>31-GEP test results, tumor clinicopathologic data, and clinical outcomes were derived from three non-overlapping published study cohorts, including prospectively-tested patients6, 7 and archival tumor specimens3 , as well as an independent archival tumor cohort (total n=1479).<br><br>Intervention: GEP classification<br><br>Outcome Measures:	*Results:* The first recurrent/distant metastatic event occurred in a 0.3 mm tumor and thus 5-year RFS and DMFS survival rates are 100% for tumors ≤0.2 mm and significantly different with 31-GEP class in patients with tumors 0.3-1.0 mm thick by logrank test.<br><br>Most changes in patient management for consecutively tested melanomas after 31-GEP testing were made for tumors ≥0.3 mm in Breslow thickness.<br><br>*Notes:*	*Describe conclusions relative to question:*<br><br>Appropriate use of the 31-GEP test for management decisions was found to be in cutaneous melanoma tumors ≥0.3 mm thick.<br><br>*Critiques of Methodology:* Not prospective<br><br>Relatively short follow-up of just over 3 years<br><br>Large N but mixed cohort from several studies<br><br>Uses "change in patient management" to indicate validity but no evidence that the change is correct.<br><br>Methodology: Claims of management change

				RFS, DMFS<br>DFS<br><br>Follow-Up: 3.3 years<br><br>*Notes*:		overstated. No SLNB results presented. No comparison with mitotic rate. Class 2B tumors – 50% ulcerated – unsurprising worse outcome. Survival data but no Cox regression analysis

	Summary of Evidence Table<br>Question 1, 2					
Level of Evidence*^<br><br>Choose one:	Study Citation<br>Author(s), Year, Title, Journal, Volume, Page #s	Funding Source	Study Methodology	Objective & Methods	Results	Conclusions
Strong<br>☐EL 1; RCT<br>☐EL 1; MRCT<br><br>Intermediate<br>☐EL 2; MNRCT<br>☐EL 2; NMA<br>☐EL 2; NRCT<br>☐EL 2; PCS<br>☐EL 2; RCCS<br>☐EL 2; NCCS<br>☐EL 2; CSS<br>☐EL 2; ES<br>☐EL 2; OLES<br>☐EL 2; PHAS<br><br>Weak<br>☐EL 3; DS<br>☐EL 3; ECON<br>☐EL 3; CCS<br>☐EL 3; SCR<br>☐EL 3; PRECLIN<br>☐EL 3; BR<br><br>No Evidence<br>☒EL 4; NE<br>☐EL 4: O	Mirsky R, Prado G, Svoboda R, Glazer A, Rigel D. Management Decisions Made by Physician Assistants and Nurse Practitioners in Cutaneous Malignant Melanoma Patients: Impact of a 31-Gene Expression Profile Test. *J Drugs Dermatol* 2018;17(11):1220-1223 (31)<br><br>*Country:* USA	*Source:*<br>None stated<br><br>1 author: consultant to Castle Biosciences.<br><br>3 authors: participated in a research fellowship which was partially funded by Castle Biosciences	*Methodology:*<br>Questionnaire survey.<br>Hypothetical management.<br><br>Online survey given to PA/NPs with six melanoma patient vignettes.<br>Effect of lower and higher risk 31-GEP test results on management measured.	*Stated Objective:*<br>Change in practice behavior.<br><br>To determine the impact of 31-GEP test results on management decisions made by dermatology PA/NPs for CM patients.<br><br>Survey study<br><br>*Study Population and Setting:*<br>Nurse Practitioners and Physician Assistants<br><br>NPs and Pas – questionnaires sent<br><br>*N:* 164<br><br>*Intervention:* Online survey with vignettes of pt and 31-GEP results.<br>Questionnaires regarding practice.<br><br>*Outcome Measures:*<br>-Proportion PA/NPs alter recommendation SLNB.<br>-Follow-up imaging and intervals recommended.<br><br>Referral rates for SNB and radiology.<br><br>*Follow-Up*: none<br><br>*Notes:*	*Results:*<br><br>Lower risk 31-GEP led to statistically significant decrease in recommended SLNB, imaging or quarterly follow-up.<br><br>Low risk – decreased referral rate for SNB.<br><br>High risk – increased rate of referral for SNB and radiology.<br>All hypothetical – no patients involved.<br><br>*Notes:*	*Describe conclusions relative to question:*<br><br>31-GEP results significantly impact management decisions by dermatology PA/NPs.<br><br>Routine use of GEP would change patient management.<br><br>*Critiques of Methodology:*<br><br>-Survey of GEP on APP management does not evaluate the validity of the test.<br>-Was survey voluntary or was there an incentive.

Level of Evidence*^ Choose one:	Summary of Evidence Table Question 1, 2<br>Study Citation Author(s), Year, Title, Journal, Volume, Page #s	Funding Source	Study Methodology	Objective & Methods	Results	Conclusions
**Strong**<br>☐EL 1; RCT<br>☐EL 1; MRCT<br>**Intermediate**<br>☐EL 2; MNRCT<br>☐EL 2; NMA<br>☐EL 2; NRCT<br>☐EL 2; PCS<br>☒EL 2; RCCS<br>☐EL 2; NCCS<br>☐EL 2; CSS<br>☐EL 2; ES<br>☐EL 2; OLES<br>☐EL 2; PHAS<br>**Weak**<br>☐EL 3; DS<br>☐EL 3; ECON<br>☐EL 3; CCS<br>☐EL 3; SCR<br>☐EL 3; PRECLIN<br>☐EL 3; BR<br>**No Evidence**<br>☐EL 4; NE<br>☐EL 4: O	Mulder E, Johansson I, Grunhagen DJ, et al. Using a Clinicopathologic and Gene Expression (CP-GEP) Model to Identify Stage I-II Melanoma Patients at Risk of Disease Relapse. Cancers. 2022;14(12):2854. (92)<br><br>*Country*<br>Netherlands, Sweden	*Source*: This work was supported by the Netherlands Enterprise Agency (IK18007).<br><br>3 authors: SkylineDx employees<br><br>3 authors: options stakeholders of SkylineDx<br><br>3 authors: rec'd two Health Holland research grants for consortium projects with SkylineDx<br><br>1 author: rec'd institutional research grants from SkylineDx	*Methodology:* Retrospective cohort study.<br><br>Determine 5-year recurrence-free survival (RFS) of low- and high-risk CP-GEP in patients with negative SLN.	*Stated Objective:* CP-GEP and recurrence risk.<br><br>"The primary aim of this retrospective study was to assess the performance of a clinicopathologic and gene expression (CP-GEP) model, a model originally developed to predict sentinel node metastasis, to identify patients with stage I–II melanoma at risk of disease relapse."<br><br>☐Prospective<br>☒Retrospective<br><br>*Study Population and Setting:* Multi-site study<br>Swedish and Dutch patients without nodal metastases<br><br>*N:* 535<br><br>*Intervention:* CP-GEP, all patients had negative GEP CP-GEP assessment<br><br>*Outcome Measures:* RFS<br><br>*Follow-Up:*<br>More than 75% had >5-year f/u<br>medial FU not stated.<br>75.7% more than 5 yr FU available. 26% up to 10 yrs<br><br>*Notes:* EORTC model was also examined	*Results:*<br>Low and high-risk CP-GEP was able to discern 5-year RFS in a population of SLN-negative patients.<br><br>"In total, 535 patients (stage I–II) were included. CP-GEP stratification among these patients resulted in a 5-year RFS of 92.9% (95% confidence interval (CI): 86.4–96.4) in CP-GEP low-risk patients (n = 122) versus 80.7% (95%CI: 76.3–84.3) in CP-GEP high-risk patients (n = 413; hazard ratio 2.93 (95%CI: 1.41–6.09), p < 0.004). According to the EORTC nomogram, 25% of the patients were classified as having a 'low risk' of recurrence (96.8% 5-year RFS (95%CI 91.6–98.8), n = 130), 49% as 'intermediate risk' (88.4% 5-year RFS (95%CI 83.6–91.8), n = 261), and 26% as 'high risk' (61.1% 5-year RFS (95%CI 51.9–69.1), n = 137)."<br><br>Notes:	*Describe conclusions relative to question:*<br>Low and high-risk CP-GEP was able to discern 5-year RFS in a population of SLN-negative patients.<br><br>Supported by findings.<br><br>"in its present form, the CP-GEP model is not yet suitable for clinical practice as the current false positive rate is too high to select patients for adjuvant therapy and would result in overtreatment."<br><br>*Critiques of Methodology:*<br><br>No f/u to account for false negative results.<br><br>Two institutions<br>Limited dataset

	Summary of Evidence Table<br>Question 2					
Level of Evidence*^<br><br>Choose one:	Study Citation<br>Author(s), Year, Title, Journal, Volume, Page #s	Funding Source	Study Methodology	Objective & Methods	Results	Conclusions
Strong<br>☐EL 1; RCT<br>☐EL 1; MRCT<br><br>Intermediate<br>☐EL 2; MNRCT<br>☐EL 2; NMA<br>☐EL 2; NRCT<br>☒EL 2; PCS<br>☐EL 2; RCCS<br>☐EL 2; NCCS<br>☐EL 2; CSS<br>☐EL 2; ES<br>☐EL 2; OLES<br>☐EL 2; PHAS<br><br>Weak<br>☐EL 3; DS<br>☐EL 3; ECON<br>☐EL 3; CCS<br>☐EL 3; SCR<br>☐EL 3; PRECLIN<br>☐EL 3; BR<br><br>No Evidence<br>☐EL 4; NE<br>☐EL 4: O	Podlipnik S, Carrera C, Boada A, et al. Early outcome of a 31-gene expression profile test in 86 AJCC stage IB-II melanoma patients. A prospective multicentre cohort study. *J Eur Acad Dermatol Venereol*. 2019 May;33(5):857-862 (109)<br><br>*Country:* Spain	*Source:*<br>Funding for this project was provided by Castle Biosciences, Inc. The research at the Melanoma Unit in Barcelona is partially funded by Spanish Fondo de Investigaciones Sanitarias grants 09/1393, 12/00840 and 15/00716; CIBER de Enfermedades Raras of the Instituto de Salud Carlos III, Spain, co-financed by European Development Regional Fund "A way to achieve Europe" ERDF; European Commission under the 6th Framework Programme, Contract No. LSHC-CT-	*Methodology:*<br>Prospective multicenter cohort study.<br><br>AJCC stage IB+II<br>N=86<br><br>Called prospective multicentre cohort study but appears to be case series identified retrospectively based on criteria only known after operation.<br><br>*Methodology:*<br>Prospective cohort study<br><br>retrospective cohort study (using a "prospectively evaluated cohort")	*Stated Objective:*<br>To evaluate the early prognostic performance of a genetic signature.<br><br>To study the prognostic performance of 31-GEP Test on recurrence.<br><br>☒Prospective (follow-up)<br>☐Retrospective (identification)<br>Retrospective study of prospectively collected data<br><br>*Study Population and Setting:*<br>Spanish cancer centers<br>Mean T depth 2.5 mm<br>Multicenter Stage IB-II patients (April 2015-December 2016)<br><br>Multicenter cohort in Spain<br><br>*N:* N=86<br>86 patients with stage Ib/II melanoma.<br><br>*Intervention:* DecisionDx test performed on primary tumors.<br>GEP test done after WE and SLNB; patients were followed prospectively.<br><br>*Outcome Measures:*<br>Relapse/recurrence<br>Metastasis at median f/u of 26 months	*Results:*<br>31 GEP assay can help prognosticate patient with melanoma independent of AJCC staging and age.<br><br>53 of 86 Class I low risk<br>33 of 86 Class 2 high risk<br><br>Class 2 had mean BD 3.7 mm vs 1.7 mm Class I.<br><br>14/62 IB/IIA were Class 2<br>5/24 IIB/IIC were Class 1<br><br>All relapses w/in 12 months and in Class 2 patients.<br><br>All patients (n=7) who developed recurrence were in class II group; GEP assay independent significant predictor of recurrence.<br><br>0% recurrence in low risk group (as defined by GEP testing);<br>8.1% recurrence in high risk group;<br><br>Class 2 (high risk group) was an independent prognostic factor for relapse after adjusting for stage and age.	*Describe conclusions relative to question:*<br>31-GEP assay can help in the prognostication of patients with cutaneous melanoma at risk of relapse and help guide surveillance strategies.<br><br>Unclear if test provides benefit over AJCC stage for predicting relapse at 20 mos.<br><br>31-GEP test can correctly predict patients who relapse.<br><br>GEP results (high risk, or Class 2) is prognostic<br><br>*Critiques of Methodology:*<br>Small patient cohort size; only seven metastases;<br>Small event rate for multivariate analysis;

		2006-018702 (GenoMEL) and by the European Commission under the 7th Framework Programme, Diagnoptics; The National Cancer Institute (NCI) of the US National Institute of Health (NIH) (CA83115), a grant from "Fundacio La Marat o de TV3, 201331-30 ", Catalonia, Spain; CERCA Programme / Generalitat de Catalunya, and a grant from "Fundacion Cient ıfica de la Asociacion Espa nola Contra el C ~ ancer ", Spain. Part of the work was carried out at the Esther Koplowitz Center, Barcelona<br>The sponsors had no role in the design and conduct of the study, nor in the collection, analysis and interpretation		Recurrence<br>Disease-free Recurrence<br><br>*Follow-Up:* 26 months<br>: median 26 months (IQR 22-30 months)<br>26 months (median) based on "routine" surveillance protocols.<br><br>*Notes:*	*Notes:*<br>86/88 test successful.<br><br>Type of relapse not stated.	Relatively short follow-up;<br>Cohort limited to Spanish Caucasians;<br><br>Excluded SLN+ patients;<br><br>Table 2 – MVA Age is not significant in UV but included in MVA -is MVA valid?<br><br>No power calculation<br><br>Small patient numbers (7 patients developed metastases)—small numbers for robust multivariate analysis.<br>Only 7/33 patients class II relapse (21.2%).<br><br>The title suggests a prospective study, but this is a retrospective study (using prospectively captured data of patients who underwent GEP testing 2015-2016 and were followed for a median of 26 months).

		of data, nor in the preparation, review and approval of the manuscript nor in the decision to submit the manuscript for publication.<br><br>The authors declare that they have no COI.				Small cohort (does not appear to include consecutive patients—there may be some patient selection bias)

	Summary of Evidence Table<br>Question 2					
Level of Evidence*^<br><br>Choose one:	Study Citation<br>Author(s), Year, Title, Journal, Volume, Page #s	Funding Source	Study Methodology	Objective & Methods	Results	Conclusions
Strong<br>☐EL 1; RCT<br>☐EL 1; MRCT<br><br>Intermediate<br>☐EL 2; MNRCT<br>☐EL 2; NMA<br>☐EL 2; NRCT<br>☒EL 2; PCS<br>☐EL 2; RCCS<br>☐EL 2; NCCS<br>☐EL 2; CSS<br>☐EL 2; ES<br>☐EL 2; OLES<br>☐EL 2; PHAS<br><br>Weak<br>☐EL 3; DS<br>☐EL 3; ECON<br>☐EL 3; CCS<br>☐EL 3; SCR<br>☐EL 3; PRECLIN<br>☐EL 3; BR<br><br>No Evidence<br>☐EL 4; NE<br>☐EL 4: O	Schuitevoerder D, Heath M, Cook RW, et al. Impact of Gene Expression Profiling on Decision-Making in Clinically Node Negative Melanoma Patients after Surgical Staging. *J Drugs Dermatol*. 2018; (17) 2. 196-199 (33)<br><br>*Country:*<br>USA	*Source:*<br>None stated<br><br>2 authors: employees of Castle Biosciences<br><br>2 authors: consultants to Castle Biosciences	*Methodology:*<br>Patients with primary melanoma staged with SLNB – GEP performed alongside clinical staging on primary.<br><br>Melanoma patients surgically staged by SLNB.<br><br>Excluded if GEP test without surgical staging.<br><br>Patients classified by recommended follow-up: 1. Derm only, 2) Surg Onc, 3) Surg onc + adj trial, 4) med onc and surg onc.<br><br>NOTES<br>OSHU will enroll all pt into an industry-sponsored prospective	*Stated Objective:*<br><br>Determine impact of GEP results on the management of clinically node negative cutaneous melanoma patients staged w SLNB.<br><br>☒Prospective<br>☐Retrospective<br><br>Retrospective review of prospectively collected data<br><br>*Study Population and Setting*:<br><br>*N:* 90/118 had SLNB and GEP testing (118 GEP testing)<br><br>*Intervention:*<br>GEP on primary<br><br>*Outcome Measures:*<br>CM patient follow-up<br>Allocation to follow-up regimen<br><br>*Follow-Up:* Not listed, not applicable<br><br>*Notes:*<br>Adj trial stage and med onc referral categorized separately but seems like should overlap.<br><br>What adj for stage I?	*Results*:<br>Decision tree analysis of which follow-up cohort suggested that 42% of decision making process was GEP result.<br><br>Of 90, 52 (58%) were Class 1 and 38 (42%) Class 2.<br><br>Stage I: 87% Class 1<br>Stage II: 33% Class 1<br>Stage III: 50% Class 1<br><br>Stage I, Class 1 more likely fu derm alone compared to Class 2 (82% v 0%).<br><br>Stage I, Class 2 more likely fu Surg Onc +/- adjuvant trial (18% vs 100%, P<0.001).<br><br>Stage II, Class 1 derm alone 21% v 0%)<br>Stage II, Class 2 follow up with surgery + recommendation for adj trials (64% vs 36%; p<0.05).<br><br>Clinical follow-up recs for Class 1 were significantly different from Class 2 (p<0.001).<br><br>Tree-based prediction model binary class I/IIA vs IIB/IIC indicated that GEP accounted for 52% of decision for follow-up.	*Describe conclusions relative to question:*<br>GEP results may change follow-up pathway for patients.<br><br>Follow-up patterns with Class 1 and Class 2 GEP patients differed significantly at this institution.<br><br>*Critiques of Methodology:*<br>One institution's use of GEP for follow-up<br><br>Small N<br><br>Classification bw adj trial and med onc referral seem like would have overlap.<br><br>Excluded GEP pt wo surgical staging but uncertain exact inclusion criteria i.e. all pt w SLN, all pt seen by surg onc

			clinical use trial, partnering with cooperative oncology groups to design adjuvant trials for node negative pt that include stratification by GEP class.		Stratifying for T-stage and GEP found T-stage accounted for 43% of the decision on follow-up, GEP for 42%, and ulceration for 15%.<br><br>___<br>*Notes:*<br>Text of stage II, Class 2 unclear exactly what 64 v *36%* is. Which is surgery + adj trial and what is the other category ie derm alone, surgery alone. Does not match exactly w Fig. 3 with appears to be *32%* Surgical oncology.	Authors are employees of Castle Bioscience – not independent<br><br>No follow-up therefore false-negative rate not available.

	Summary of Evidence Table<br>Question 2					
Level of Evidence*^<br><br>Choose one:	Study Citation<br>Author(s), Year, Title, Journal, Volume, Page #s	Funding Source	Study Methodology	Objective & Methods	Results	Conclusions
**Strong**<br>☐EL 1; RCT<br>☐EL 1; MRCT<br><br>**Intermediate**<br>☐EL 2; MNRCT<br>☐EL 2; NMA<br>☐EL 2; NRCT<br>☐EL 2; PCS<br>☐EL 2; RCCS<br>☐EL 2; NCCS<br>☐EL 2; CSS<br>☐EL 2; ES<br>☐EL 2; OLES<br>☐EL 2; PHAS<br><br>**Weak**<br>☐EL 3; DS<br>☐EL 3; ECON<br>☒EL 3; CCS<br>☐EL 3; SCR<br>☐EL 3; PRECLIN<br>☐EL 3; BR<br><br>**No**<br>☐EL 4; NE<br>☐EL 4: O	Scott AM, Dale PS, Conforti A, Gibbs JN. Integration of a 31-Gene Expression Profile Into Clinical Decision-Making in the Treatment of Cutaneous Melanoma. *Am. Surg*. 2020;86(11):1561-1564.<br>(67)<br><br>*Country:* United States	*Source:* No financial support for the research, authorship, and/or publication of this article	*Methodology:*<br>Retrospective Case series<br><br>31 gene GEP in intermediate melanoma and impact on clinical management	*Stated Objective:*<br><br>☐Prospective<br>☒Retrospective<br><br>*Study Population and Setting:*<br>Single institution<br><br>*N:*<br><br>26 pts, complete data present in 23<br><br>*Intervention:* Applied GEP post biopsy to determine follow up protocol<br><br>Documentation of change of care due to GEP (Two patients excluded SLNB)<br><br>*Outcome Measures:*<br><br>*Follow-Up:*<br><br>*Notes:*	*Results:*<br>SLNB was omitted in Two patients low risk over age 65.<br><br>18 patients had a SLN bx, two of which were positive and both class 2b. These two patients were characterized by the authors as "having their management changed by the GEP," but it is unclear why they stated that as the patients were having a SLNB anyway.<br><br>Clinical decision- making was altered such that SLNB was omitted in two cases in which the patients were found to be low risk by 31-GEP with age >65 years. Eighteen patients underwent SLNB which returned positive in two patients of Class 2B (P = .497). In seven cases, sentinel lymph nodes were obtained despite low- risk classification by 31- GEP, but higher clinical suspicion was present. Follow-up routines for patients also varied based on 31- GEP results, but this was not fully evaluated for the purposes of this study.<br><br>*Notes:*	*Describe conclusions relative to question:*<br><br>No statistical results to change management, but conclusions by authors still were to change practice and incorporate the GEP selectively.<br><br>*Critiques of Methodology:*<br><br>Too small to characterize utility and no real structure to the protocol. No follow up. Very small cohort, no comparator arm, impact of changes not measured.

	Summary of Evidence Table<br>Question 1, 2, 3					
Level of Evidence*^<br><br>Choose one:	Study Citation<br>Author(s), Year, Title, Journal, Volume, Page #s	Funding Source	Study Methodology	Objective & Methods	Results	Conclusions

**Strong**<br>☐EL 1; RCT<br>☐EL 1; MRCT<br><br>**Intermediate**<br>☐EL 2; MNRCT<br>☐EL 2; NMA<br>☐EL 2; NRCT<br>☐EL 2; PCS<br>☐EL 2; RCCS<br>☐EL 2; NCCS<br>☐EL 2; CSS<br>☐EL 2; ES<br>☐EL 2; OLES<br>☐EL 2; PHAS<br><br>**Weak**<br>☐EL 3; DS<br>☐EL 3; ECON<br>☐EL 3; CCS<br>☐EL 3; SCR<br>☐EL 3; PRECLIN<br>☐EL 3; BR<br><br>**No Evidence**<br>☒EL 4; NE<br>☐EL 4: O	Svoboda RM, Glazer AM, Farberg AS, Rigel DS. Factors Affecting Dermatologists' Use of a 31-Gene Expression Profiling Test as an Adjunct for Predicting Metastatic Risk in Cutaneous Melanoma. J. Drugs Dermatol. 2018;17(5):544-547. (35)<br><br>*Country:*<br>USA	*Source:*<br><br>This study was funded in part by a grant from Castle Biosciences Inc.<br><br>1 author: consultant to Castle Bioscience<br><br>1 author: served on advisory board for Castle Bioscience<br><br>2 authors: participated in research fellowship that was partially funded by Castle Bioscience	*Methodology:*<br><br>Dermatologists answered questionnaire with four clinical vignettes to determine the impact of Breslow thickness, ulceration and SLNB status decision to order GEP test.<br><br>Survey-based study performed at a conference.	*Stated Objective:*<br>Clinical factors that impact dermatologists' decisions to utilize 31-GEP.<br><br>☒Prospective<br>☐Retrospective<br><br>*Study Population and Setting*:<br>Dermatology questionnaire at a national conference.<br><br>Panel of clinicians.<br>Survey<br><br>*N:* 181/187<br><br>*Intervention:*<br>Questionnaire with four clinical vignettes.<br><br>Opinion regarding management<br><br>*Outcome Measures:*<br>Percentage of respondents who would order 31-GEP in clinical scenarios.<br><br>Recommended treatment pathway.<br><br>*Follow-Up*: N/A<br><br>*Notes:*	*Results:*<br>A majority of patients would recommend GEP test."<br><br>Breslow thickness ≥ 0.5 mm, majority dermatologists would order GEP. Ulceration was associated with a statistically significant increase to recommend for all but the thickest ≥ 2.1 mm. For thin tumor (0.26 mm) ulceration significantly changed from 22% to 67%, p <0.001). A negative SLNBx only associated with statistically significant increase in the percentage for the thinnest tumors (22% to 34%, p=0.033).<br><br>Impact of GEP test result on T1b 0.76-1.0 mm melanoma:<br>Class 1 result -91% respondents reported less likely to recommend SLNB.<br>Class 2 - 81% would make more likely to recommend SLNB.<br><br>*Notes:*	*Describe conclusions relative to question:*<br>Not relevant to current clinical practice.<br><br>Ulceration most important factor deciding to order 31-GEP.<br><br>*Critiques of Methodology:*<br>Authors do not state whether the survey was taken at a session paid for by Castle Bioscience.<br><br>Authors have conflict of interest – was the survey voluntary<br><br>-Survey of dermatologists' ordering pattern does not evaluate the validity of the test.<br>- Selection bias in those willing to take the survey<br>-was survey voluntary or was there an incentive.

Level of Evidence*^<br><br>Choose one:	Study Citation<br>Author(s), Year, Title, Journal, Volume, Page #s	Funding Source	Study Methodology	Objective & Methods	Results	Conclusions
**Strong**<br>☐EL 1; RCT<br>☐EL 1; MRCT<br><br>**Intermediate**<br>☐EL 2; MNRCT<br>☐EL 2; NMA<br>☐EL 2; NRCT<br>☐EL 2; PCS<br>☐EL 2; RCCS<br>☐EL 2; NCCS<br>☐EL 2; CSS<br>☐EL 2; ES<br>☐EL 2; OLES<br>☐EL 2; PHAS<br><br>**Weak**<br>☐EL 3; DS<br>☐EL 3; ECON<br>☐EL 3; CCS<br>☐EL 3; SCR<br>☐EL 3; PRECLIN<br>☐EL 3; BR<br><br>**No Evidence**<br>☒EL 4; NE<br>☐EL 4: O	Swetter SM, Tsao H., Bichakjian CK, Curiel-Lewandrowski C, Ender DE, Gershewald JE, et al. Guidelines of Care for the Management of Primary Cutaneous Melanoma. *J. Am. Acad. Dermatol.* 2019 Jan 1; 80(1):208-250. (117)<br><br>*Country:* USA	*Source:* None<br><br>1 author: served as an advisory board member to Castle Biosciences, receiving fees.	*Methodology*: Practice guideline development.<br><br>AAD guidelines for the management of primary cutaneous melanoma from a group of dermatologists, patient advocate, at least one surgeon<br><br>2018	*Stated Objective:*<br><br>☐*Prospective*<br>☐*Retrospective*<br><br>*Study Population and Setting:*<br><br>*N:*<br><br>*Intervention:*<br><br>*Outcome Measures:*<br><br>*Follow-Up:*<br><br>*Notes:*	*Results:*<br><br>GEP testing can stratify patients into risk categories with some degree of precision; however the data to date consists of heterogenous groups of high risk event enriched groups that may not represent the spectrum of melanoma patient population and the clinical applicability of the tests is uncertain based on the lack of surgical or therapeutic RCTS to examine outcomes with GEP testing.<br><br>Page 230 of the article addresses GEP and cautions against routine use for clinical decision-making pending appropriate clinical trials evaluating clinical usefulness of tests and whether currently available tests add to AJCC8 staging.<br><br>*Notes:*	*Describe conclusions relative to question:*<br><br>The working group discourages baseline GEP testing for prognostication and for management decisions.<br><br>AAD opinion on GEP provided on p 230.<br><br>*Critiques of Methodology:*

	Summary of Evidence Table<br>Question 1, 2					
Level of Evidence*^<br><br>Choose one:	Study Citation<br>Author(s), Year, Title, Journal, Volume, Page #s	Funding Source	Study Methodology	Objective & Methods	Results	Conclusions
Strong<br>☐EL 1; RCT<br>☐EL 1; MRCT<br><br>Intermediate<br>☐EL 2; MNRCT<br>☐EL 2; NMA<br>☐EL 2; NRCT<br>☒EL 2; PCS<br>☐EL 2; RCCS<br>☐EL 2; NCCS<br>☐EL 2; CSS<br>☐EL 2; ES<br>☐EL 2; OLES<br>☐EL 2; PHAS<br><br>Weak<br>☐EL 3; DS<br>☐EL 3; ECON<br>☐EL 3; CCS<br>☐EL 3; SCR<br>☐EL 3; PRECLIN<br>☐EL 3; BR<br><br>No Evidence<br>☐EL 4; NE<br>☐EL 4: O	Thorpe RB, Covington KR, Caruso HG, et al. Development and Validation of a Nomogram Incorporating Gene Expression Profiling and Clinical Factors for Accurate Prediction of Metastasi in Patients with Cutaneous Melanoma following Mohs MicrographicSsurgery. J. Am. Acad. Dermatol. 2022;86(4):846-853. (96)<br><br>*Country*: USA 9 Mohs private practices	*Source:*<br>Statistician provided by Castle Biosciences<br>IRB funded by Skin Cancer Trust for independent Research and Education, a 501c3 managed by Drs. Zitelli and Brodland<br><br>4 authors: employees of Castle Biosciences<br><br>4 authors: stockholders at Castle Biosciences	*Methodology:*<br>Prospective testing with 31-GEP of patients with melanoma undergoing melanoma surgery at Mohs surgery center.<br><br>Melanoma patients with complete clinical and pathologic information including 31-GEP enrolled from the nine centers<br><br>Years of enrollment not stated.<br><br>All pts had Mohs with six mm initial margins with further margins until clear by MART1 IHC.	*Stated Objective:*<br>To develop a nomogram for predicting metastasis using 31-GEP and T stage.<br><br>To define a nomogram for practical clinical use using 31-GEP.<br><br>☒Prospective<br>☐Retrospective<br><br>*Study Population and Setting:*<br>Patients undergoing melanoma surgery at nine Mohs surgery centers.<br><br>Nine Mohs Private Derm Practices<br><br>*N:* 1124 enrolled – data presented is from 684 patients followed for min 1 yr or a "metastatic event" as those used for nomogram development (p847).<br><br>*Intervention:* Mohs + 31-GEP<br><br>*Outcome Measures:*<br>Nomogram development for predictng metastasis using<br><br>Risk of metastasis using a nomogram<br><br>*Follow-Up:*	*Results:*<br><br>31-GEP test and T stage offers simplest nomogram with lowest Bayesian information score—validated in a separate cohort of 905 patients.<br><br>Class 2B patients do worse (53/684 = 7.7% of the cohort<br>Don't see where the nomogram considering Breslow depth and 31-GEP adds value over either alone (see Table II) except maybe T2-T3a and shifts %s in clinically unmeaningful way – wouldn't change f/u or rx recommendations.<br><br>*Notes:*<br><br>Mohs for invasive melanoma is unsupported by RCT evidence.<br>Graphs present five year survival without numbers at risk data & P value doesn't state what the comparison is (class IA,1B,2A all appear to overlap on the RFS and DMFS curves)<br>Demographics in supplemental table mean BT 0.5 mm (0.1-13.0 mm)	*Describe conclusions relative to question:*<br><br>31-GEP test and T stage can provide prognostic information for metastasis.<br><br>Question biopsy as non-invasive (need to do it to get T category which they refer to as T stage).<br><br>*Critiques of Methodology:*<br>SLNB not done for many patients and not included in the nomogram<br><br>Limited to patients undergoing surgery at Mohs surgery centers which may reflect a selection bias (very few T2B and higher lesions)<br>Relatively short f/u particularly for earlier T stage lesions<br><br>Study design flawed:<br>Patient selection

				At least one year f/u or metastatic event; median f/u 3.2 years<br><br>Median 3.2 years<br>___<br>*Notes:*	and correlate with 31-GEP class for entire 1124 pt cohort not the 684 used to develop nomogram.	No SLNB in 654 of the 684 pts<br>No prospective validation cohort for nomogram – used an "archival cohort of 901 stage I-III patients w/ median 6.1 yrs f/u from 22 centers (no ref) to "validate" which is different from this study population.

	Summary of Evidence Table<br>Question 2					
Level of Evidence*^<br><br>Choose one:	Study Citation<br>Author(s), Year, Title, Journal, Volume, Page #s	Funding Source	Study Methodology	Objective & Methods	Results	Conclusions
**Strong**<br>☐EL 1; RCT<br>☐EL 1; MRCT<br><br>**Intermediate**<br>☐EL 2; MNRCT<br>☐EL 2; NMA<br>☐EL 2; NRCT<br>☐EL 2; PCS<br>☒EL 2; RCCS<br>☐EL 2; NCCS<br>☐EL 2; CSS<br>☐EL 2; ES<br>☐EL 2; OLES<br>☐EL 2; PHAS<br><br>**Weak**<br>☐EL 3; DS<br>☐EL 3; ECON<br>☐EL 3; CCS<br>☐EL 3; SCR<br>☐EL 3; PRECLIN<br>☐EL 3; BR<br><br>**No Evidence**<br>☐EL 4; NE<br>☐EL 4: O	Williams A, Hamilton O, Likar C, Thomay A, Garland-Kledzik M. "The Benefit Of Positron Emission Tomography/Computed Tomography In Stage I And Stage II Melanomas With High-Risk Decisiondx-Melanoma Scores". Am Surg. 2022;88(7):1446-1451 (97)<br><br>*Country:* USA<br>West Virginia School of Medicine	*Source:*<br>None declared	*Methodology:*<br>Retrospective cohort study-<br><br>295 pts who had 31-GEP assessed for eligibility and 66 identified (no information provided on pts who did not have 31-GEP, if any).	*Stated Objective:*<br>Assess the incidence of PET identified recurrent disease in patients with Class 2 Decision Dx category.<br><br>Assess benefit of PET-CT+Brain MRI screening for SLNB-negative pts with Class 2 31-GEP for 3 years post-op.<br><br>☐Prospective<br>☒Retrospective<br><br>*Study Population and Setting:*<br>Patients with melanoma undergoing Decision DX testing (2014-2021) and classified as Class II.<br><br>Academic Practice single institution.<br><br>*N:* 66 Class II patients with negative SLNB.<br><br>66 of 297 patients 2014-2021 with melanoma who had 31-GEP testing performed were SLN-negative and had 31-GEP test resulted as Class 2.<br><br>*Intervention:*<br>Decision Dx testing and PETCT yearly and brain MRI.	*Results:*<br><br>12.1% of patients (8/66) developed metastases as detected by PETCT; no patients with stage IA or IB developed metastasis.<br><br>% with distant disease identified on screening PET-CT + brain MRI (12.1% in study vs <3% for Stage I/II melanoma historical reports).<br>All eight pts with recurrence had Stage II melanoma and 5 of 8 IIC<br>12 pts had biopsies +/- additional imaging based on PET-CT and/or Brain MRI findings and 4 of 12 were benign.<br><br>Notes:	*Describe conclusions relative to question:*<br><br>Recommend Decision DX for stage II and higher patients and for PETCT and brain MRI among those patients with class II Decision DX score.<br><br>Recommend imaging screening based on these data for Class 2 patients.<br>No comparison or stats on screening by AJCC stage (IIC or IIB/C vs 31-GEP class).<br>Data on benefit of detection of asymptomatic metastasis in f/u of cutaneous melanoma patients unknown.<br><br>*Critiques of Methodology:*<br>Retrospective study with

				Annual PET-CT + Brain MRI<br><br>*Outcome Measures:*<br>Metastases detected by PETCT<br>% with distant disease identified on screening PET-CT + brain MRI<br><br>*Follow-Up:*<br>Within 3 years<br>median 16 months<br><br>*Notes:*		potential selection biases.<br><br>Incidence of metastases is fairly high in stage II in patients not undergoing Decision Dx; the majority of recurrences were in stage IIC patients (5/8) which would be expected without GEP Testing.<br><br>Relatively short follow-up.<br><br>Retrospective based on test receipt and imaging.<br>Short f/u 16 months median.<br>No comparator group.<br>Rec overstated based on these data.

	Summary of Evidence Table<br>Question 2					
Level of Evidence*^<br><br>Choose one:	Study Citation<br>Author(s), Year, Title, Journal, Volume, Page #s	Funding Source	Study Methodology	Objective & Methods	Results	Conclusions
☐EL 1; RCT<br>☐EL 1; MRCT<br><br>☐EL 2; MNRCT<br>☐EL 2; NMA<br>☐EL 2; NRCT<br>☐EL 2; PCS<br>☐EL 2; RCCS<br>☐EL 2; NCCS<br>☐EL 2; CSS<br>☐EL 2; ES<br>☐EL 2; OLES<br>☐EL 2; PHAS<br><br>☐EL 3; DS<br>☐EL 3; ECON<br>☒EL 3; CCS<br>☐EL 3; SCR<br>☐EL 3; PRECLIN<br>☐EL 3; BR<br><br>☐EL 4; NE<br>☐EL 4: O<br><br>Strong / Intermediate / Weak / No Evidence	Wisco OJ, Marson JW, Litchman GH, et al. Improved cutaneous melanoma survival stratification through integration of 31-gene expression profile testing with the American Joint Committee on Cancer 8th Edition Staging. *Melanoma Res.* Apr 1, 2022;32(2):98-102.<br><br>*Country*: USA	*Source:*<br>This research was funded in part by Castle Biosciences. The funding source assisted in the collection, management and analysis of data. The funding source had no role in the design and conduct of the study; interpretation of the data, preparation, review or approval of the manuscript and decision to submit the manuscript.<br><br>6 authors: employees of Castle Biosciences<br><br>6 authors are options holders of Castle Biosciences	*Methodology:*<br>Retrospective case series<br><br>Samples of CM from 1998-2016 with at least 5 years of follow up were obtained.	*Stated Objective:*<br>Prediction of melanoma survival by GEP classification<br><br>Determine if 31-GEP could improve upon AJCC8 in prediction of MSS<br><br>☐Prospective<br>☒Retrospective<br><br>*Study Population and Setting:*<br>multicenter – academic cancer centers.<br><br>Cohort of patients in whom recurrence data was previously published (Greenhaw JAAD 2000, ref 5). This paper focused on MSS.<br><br>*N:* 901<br><br>*Intervention:* GEP-classification<br>31-GEP<br><br>*Outcome Measures:*<br>Melanoma specific survival<br><br>*Follow-Up:* 6.1 years<br>Median 6.1 years<br><br>*Notes:*	*Results:* Cox – HR for melanoma death from class 2B compared to 1A is 6.44 [2.61-15.85]<br><br>Breslow thickness and SNB status also independent predictors of survival – but ulceration status was not.<br><br>Specificity/Sensitivity/PPV/NPV are inappropriate metrics for this analysis and should be ignored.<br><br>No survival curves<br><br>Comparison of 1B/2A to 2B omitted – selective data reporting again?<br><br>31-GEP provided additional prognostic information within stage for MSS. In stage I there were very few recurrences limiting analysis. In Stage II and III differences were more pronounced	*Describe conclusions relative to question:*<br><br>Class 2B GEP status indicates poor prognosis for melanoma<br><br>Results suggest that GEP status is surrogate marker for ulceration, which indicates poorly differentiated melanoma.<br><br>Incorporation of GEP may improve prognostication compared to AJCC alone.<br><br>Class IA vs IIB but not IB/IIA included.<br><br>*Critiques of Methodology:*<br>Reuse of old data (see previous publications).

		2 author: speakers bureau Castle Biosciences<br><br>2 authors are a consultant for Castle Biosciences<br><br>1 author: advisory board member Castle Bioscience			*Notes:*	Data only selectively presented<br><br>The source of the patients is unclear. Presumably many have been previously published.<br><br>Selective reporting of data. No crosstab of tumor characteristics and GEP-class.<br><br>Details of this cohort are very poorly described.

Level of Evidence*^<br><br>Choose one:	Summary of Evidence Table<br>Question 2<br>Study Citation<br>Author(s), Year, Title, Journal, Volume, Page #s	Funding Source	Study Methodology	Objective & Methods	Results	Conclusions
**Strong**<br>☐EL 1; RCT<br>☐EL 1; MRCT<br><br>**Intermediate**<br>☐EL 2; MNRCT<br>☐EL 2; NMA<br>☐EL 2; NRCT<br>☐EL 2; PCS<br>☒EL 2; RCCS<br>☐EL 2; NCCS<br>☐EL 2; CSS<br>☐EL 2; ES<br>☐EL 2; OLES<br>☐EL 2; PHAS<br><br>**Weak**<br>☐EL 3; DS<br>☐EL 3; ECON<br>☐EL 3; CCS<br>☐EL 3; SCR<br>☐EL 3; PRECLIN<br>☐EL 3; BR<br><br>**No Evidence**<br>☐EL 4; NE<br>☐EL 4: O	Zager JS, Gastman BR, Leachman S, et al. Performance of a Prognostic 31-Gene Expression Profile in an IndependentCohort of 523 Cutaneous Melanoma Patients. *BMC Cancer.* 2018;18(1):130. (36)<br><br>*Country*:<br>USA	*Source:*<br><br>This study was sponsored by Castle BioSciences, Inc., which provided funding for tissue and clinical data retrieval to contributing centers.<br><br>10 authors: previously served as paid consultants to Castle Biosciences<br><br>1 author: services as a paid clinical consultant to Castle Bioscience<br><br>4 authors: Employees to Castle Bioscience<br><br>4 authors: hold stock options for	*Methodology:*<br>GEP classified primary melanoma tumors as Class 1 or Class 2 which was correlated to clinical outcome and assessed along w AJCC v7 staging. Multi-center.<br><br>Multicentre study. Archival tissue sent for analysis.	*Stated Objective:*<br>Predictive survival value of GEP for melanoma patients.<br><br>To evaluate 31-GEP's prognostic accuracy in independent cohort of CM patients.<br><br>☐Prospective<br>☒Retrospective<br><br>*Study Population and Setting:*<br>Primary cutaneous melanoma patients staged with SNB<br><br>Multi-center (16) primary melanoma tumors<br><br>Inclusion criteria:<br>Biopsy confirmed stage I–III cutaneous melanoma diagnosed between 2000 and 2014, with at least five years of follow-up, unless there was an earlier documented recurrence or metastatic event.<br><br>*N:* 523<br><br>*Intervention:*<br>31-GEP<br>GEP staging of primary SNB<br><br>*Outcome Measures:*	*Results:*<br><br>5-year RFS, DMFS and MSS for Class 1 was 88%, 93% and 98% respectively and Class 2 was 52%, 60% and 78% (p<0.001).<br><br>Stage I pt 5-year RFS class 1- 96%, Class 2- 85%. RFS class 1A- 98%, Class 2B-73%. (p<0.001).<br><br>Stage I pt 5-year DMFS class 1- 97%, Class 2- 90%. RFS class 1A- 98%, Class 2B-87%. (p=0.05).<br><br>Stage I pt 5-year MSS class 1A- 100%, Class 2B-93%. (p<0.01).<br><br>Stage II pt 5-year RFS class 1- 74%, Class 2- 55%. p=0.043. RFS class 1A-77%, Class 2B-50%. (p=0.13).<br><br>Stage I pt 5-year DMFS class 1- 90%, Class 2- 63%. RFS class 1A- 95%, Class 2B- 57%. (p<0.001).<br><br>Stage I pt 5-year MSS class 1A- 100%, Class 2B-82%. (p=.13).<br><br>30/43 (70%) patients stage I and II with distant mets were Class 2.	*Describe conclusions relative to question:*<br><br>31-GEP is an accurate predictor of metastatic risk.<br><br>Class II GEP result is significant independent predictor of survival in multivariate analysis. However, SLNB status and Breslow thickness still significant. High Risk GEP seems to be replacing ulceration status and mitotic rate in primary risk factors. Predictor of low risk disease In SLN negative patients.<br><br>*Critiques of Methodology:*<br><br>Retrospective<br><br>Size of cohort especially as

		Castle<br>Bioscience		Recurrence-free (RFS) and distant metastasis-free (DMFS) survival.<br>MSS-secondary endpoint.<br>5-year RFS and DMFS rates<br><br>*Follow-Up:* 5 years +, unless earlier documented recurrence.<br><br>Minimum 5 years<br><br>*Notes:*	9/11 (82%) patients stage I and II who died were Class 2.<br><br>Stage IIIA 5-year RFS class 1- 72%, Class 2- 51%, p=0.015. DMFS class 1- 80%, Class 2- 54%. (p0.019). MSS class 1- 100%, Class 2- 67%. (p0.009).<br><br>In univariate analysis, DMFS Hazard ratio Class 1 was 5.4 and Class 2 was 6.6 p <0.001. Breslow thickness, ulceration, mitotic rate and SLN status also significant.<br><br>Multivariate model<br>HR for RFS<br>Breslow 1.2, p <0.001;<br>mitotic rate 0.9, NS;<br>ulceration 1.4, NS;<br>SLN positive 2.5, p<0.001;<br>GEP Class 2, 2.1, p=0.003.<br><br>HR for DMFS<br>Breslow 1.3, p <0.001;<br>mitotic rate 0.9, NS;<br>ulceration 1.2, NS;<br>SLN positive 3.0, p<0.001;<br>GEP Class 2, 2.7, p=0.002.<br><br>SLNB status<br>N=337 both SLN status and GEP.<br>5-yr RFS<br>SLN-neg/ Class 1 87%<br>SLN-neg/ Class 2 67%<br><br>5-yr DMFS<br>SLN-neg/ Class 1 93%<br>SLN-neg/ Class 2 75%<br><br>5-yr MSS	analyze smaller groups such as in stage or ulceration and fewer events<br><br>Classification Breslow thickness ≤ 1mm v > 1 mm. Should look at continuous range or more categories ie 1-2, 2-3, >4.<br><br>Statistical methodology most appropriate that I have seen so far.

					SLN-neg/ Class 1 98%<br>SLN-neg/ Class 2 92%<br><br>RFS/DMFS/MSS<br>SLN-pos/ Class 1: 61%, 74%, 93%.<br><br>SLN-pos/ Class 2: 37%, 44% and 63%.<br><br>SLN-neg/Class 1A v SLN-neg/Class 2b 90% v 60%, 96% vs 69% and 100% vs 88%<br><br>GEP v SLNB accuracy *sensitivity* R/DM/MSS Class 2 70%, 75%, and 85%, compared to SLNB-positivity 66%, 67% and 79%<br><br>GEP v SLNB accuracy *specificity* R/DM/MS mortality<br>Class 1 71%, 69%, and 64%, compared to SLNB-negativity 65%, 62% and 58%.<br><br>Positive predictive value (PPV) of Class 2 and SLN-positivity were 48%, and 52% recurrence, 40% and 42% and 19% and 21% for MSS. The PPV of class 2B was 55% for recurrence, 45% for DM and 24% for MS mortality.<br><br>NPV for Class 1 and SLN-negative were 87% and 76% for recurrence, 91% and 82% for DM and 98% and 95% for MS mortality. NPV Class 1A was 89% for recurrence, 94% for DM and 99% for MS mortality.	

						Improved sensitivity with SLNB +GEP 88% for all recurrences, 91% distant mets.<br><br>The 5-year RFS rates for Class 1 and Class 2 were 88% and 52%, respectively, and DMFS rates were 93% versus 60%, respectively (P < 0.001). The GEP was a significant predictor of RFS and DMFS in univariate analysis (hazard ratio [HR] = 5.4 and 6.6, respectively, P < 0.001 for each), along with Breslow thickness, ulceration, mitotic rate, and sentinel lymph node (SLN) status (P < 0.001 for each). GEP, tumor thickness and SLN status were significant predictors of RFS and DMFS in a multivariate model that also included ulceration and mitotic rate (RFS HR = 2.1, 1.2, and 2.5, respectively, P <0.001 for each; and DMFS HR=2.7, 1.3 and 3.0, respectively, P < 0.01 for each).<br><br>________________<br>Notes: Class IB and 2A not discussed much in paper; however, curves appear to overlap.	

Level of Evidence*^ Choose one:	Summary of Evidence Table Question 1, 2					
	**Study Citation** Author(s), Year, Title, Journal, Volume, Page #s	**Funding Source**	**Study Methodology**	**Objective & Methods**	**Results**	**Conclusions**
☐EL 1; RCT<br>☐EL 1; MRCT<br>Strong<br><br>☐EL 2; MNRCT<br>☐EL 2; NMA<br>☐EL 2; NRCT<br>☐EL 2; PCS<br>☐EL 2; RCCS<br>☐EL 2; NCCS<br>☐EL 2; CSS<br>☐EL 2; ES<br>☐EL 2; OLES<br>☐EL 2; PHAS<br>Intermediate<br><br>☐EL 3; DS<br>☐EL 3; ECON<br>☐EL 3; CCS<br>☐EL 3; SCR<br>☐EL 3; PRECLIN<br>☐EL 3; BR<br>Weak<br><br>☒EL 4; NE<br>☐EL 4: O<br>No Evidence	Zakria D, Brownstone N, Berman B, Ceilley R, Goldenberg G, Lebwohl M, Litchman G, & Siegel D. Incorporating Prognostic Gene Expression Profile Assays into the Management of Cutaneous Melanoma: An Expert Consensus Panel Report. *SKIN The Journal of Cutaneous Medicine.* 2023; 7(1): 556–569.<br><br>*Country:* USA	*Source:*<br><br>This study was funded in part by an unrestricted educational grant from Castle Biosciences.<br><br>2 author: consultant to Castle Biosciences<br><br>1 author: served on advisory boards for Castle Biosciences	*Methodology:* Review of literature; consensus study.<br><br>Literature review and consensus panel.	*Stated Objective:*<br><br>☐Prospective<br>☒Retrospective<br><br>*Study Population and Setting:*<br><br>*N:* 785 article that met search criteria; 22 validating 31-GEP; 2 11-GEP and 7 8 GEP<br><br>*Intervention:*<br><br>*Outcome Measures:*<br><br>*Follow-Up:*<br><br>*Notes*: 22 articles used to inform the panel and 11 consensus recommendations	*Results:*<br>Review of current literature with level of evidence assessments.<br><br>SORT level A recommendations were made for<br>--GEP testing can identify a high-risk subset for recurrence, distant metastasis, or death of traditionally assessed low-risk patients (e.g., SLN negative or T1a/b) (SORT Level=A)<br>--GEP testing provides clinically useful information that augments risk-aligned management decisions to both rule-in or rule-out the need for SLNB and subsequent management plans (SORT Level=A)<br><br>SORT level B recommendations were made for<br>--Integrating GEP results can increase the precision and confidence of melanoma management decisions (e.g., follow up regimens, decision for SLNB, referral to other specialties, and need for imaging) (SORT Level=B)<br><br>*Notes:*	*Describe conclusions relative to question:*<br><br>GEP tests appear to offer valuable prognostic information and assistance with clinical management of patients with cutaneous melanoma over and above AJCC staging and NCCN guidelines; there appears to be more published data supporting the clinical utility of 31- GEP test.<br><br>General support for use of GEP in practice.<br><br>*Critiques of Methodology:*<br>Conclusions do not appear to be entirely supported by data of published literature, particularly for T1 lesions<br><br>This is a consensus panel

					SORT is Strength of Recommendation Taxonomy (A, B, or C)	(recommendations made as the National Society for Cutaneous Medicine)

Question 3 + Future Research Directions

1) In adult patients with primary cutaneous melanoma, does GEP testing provide additional information and improve risk-stratification, beyond current diagnostic standards, to influence decisions for the utilization and the utility of adjuvant therapy?

Future Research Directions

What further research is needed to inform indications for GEP testing in the clinical care of patients with AJCC pT1a-pT4b (cN0M0) primary cutaneous melanoma?

	Summary of Evidence Table Question 1, 2, 3					
Level of Evidence*^<br><br>Choose one:	Study Citation Author(s), Year, Title, Journal, Volume, Page #s	Funding Source	Study Methodology	Objective & Methods	Results	Conclusions
Strong<br>☐EL 1; RCT<br>☐EL 1; MRCT<br><br>Intermediate<br>☐EL 2; MNRCT<br>☐EL 2; NMA<br>☐EL 2; NRCT<br>☐EL 2; PCS<br>☐EL 2; RCCS<br>☐EL 2; NCCS<br>☐EL 2; CSS<br>☐EL 2; ES<br>☐EL 2; OLES<br>☐EL 2; PHAS<br><br>Weak<br>☐EL 3; DS<br>☐EL 3; ECON<br>☐EL 3; CCS<br>☐EL 3; SCR<br>☐EL 3; PRECLIN<br>☐EL 3; BR<br><br>No Evidence<br>☒EL 4; NE<br>☐EL 4: O	Berman B, Ceilley R Cockerell C. Appropriate Use Criteria for the Integration of Diagnostic and Prognostic Gene Expression Profile Assays into the Management of Cutaneous Malignant Melanoma: An Expert Panel Consensus-Based Modified Delphi Process Assessment. *SKIN The Journal of Cutaneous Medicine*, 2019; 3(5): 291–306.<br><br>*Country:* USA	*Source:*<br><br>The National Society for Cutaneous Medicine (a 501(c)3 non-profit entity) funded the consensus development program. The group has received unrestricted educational grants from related companies in volved with these technologies.<br><br>5 authors: Castle Bioscience consultant<br><br>2 author: Castle	*Methodology:*<br><br>Expert panel consensus based Modified Delphi Process<br><br>3 GEP tests evaluated: 2-GEP Test, myPath from Myriad, Castle 31-GEP<br><br>Literature review	*Stated Objective:*<br><br>The objective of this expert panel was to develop a set of consensus-based AUC recommendations to guide the integration of GEP technology into the diagnosis and management of melanoma<br><br>☐Prospective<br>☐Retrospective<br><br>*Study Population and Setting*:<br>Lit review done (33 total articles)<br>Consensus panel was 9 derm/dermpath/surgeons<br><br>*N:*<br><br>*Intervention:*<br><br>*Outcome Measures:*<br><br>*Follow-Up:* None	*Results:*<br>Several clinical scenarios evaluation and given consensus based recommendations based on the taxonomy below.<br><br>One of 14 scenarios received an A-strength recommendation: • Use of the 31-GEP test to aid in the management of patients who are SLNB negative<br><br>Not applicable<br><br>*Notes:*<br>Based on SORT Taxonomy, A = Consistent, good-quality patient-oriented evidence, B = Inconsistent or limited quality patient-oriented evidence, C = Consensus, disease-oriented evidence, usual	*Describe conclusions relative to question:*<br><br>First "evidence based" framework for using GEP tests in clinical management.<br><br>*Critiques of Methodology:*

		Bioscience investigator<br><br>2 author: Castle Bioscience honoraria<br><br>3 author: Castle Bioscience advisory board		___<br>*Notes:*	practice, expert opinion, or case series	

	Summary of Evidence Table<br>Question 1, 2, 3					
Level of Evidence*^<br><br>Choose one:	Study Citation<br>Author(s), Year, Title, Journal, Volume, Page #s	Funding Source	Study Methodology	Objective & Methods	Results	Conclusions
Strong<br>☐EL 1; RCT<br>☐EL 1; MRCT<br><br>Intermediate<br>☐EL 2; MNRCT<br>☐EL 2; NMA<br>☐EL 2; NRCT<br>☐EL 2; PCS<br>☐EL 2; RCCS<br>☐EL 2; NCCS<br>☐EL 2; CSS<br>☐EL 2; ES<br>☐EL 2; OLES<br>☐EL 2; PHAS<br><br>Weak<br>☒EL 3; DS<br>☐EL 3; ECON<br>☐EL 3; CCS<br>☐EL 3; SCR<br>☐EL 3; PRECLIN<br>☐EL 3; BR<br><br>No Evidence<br>☐EL 4; NE<br>☐EL 4: O	Cook RW, Middlebrook B, Wilkinson J, et al. Analytic validity of DecisionDx-Melanoma, a gene expression profile test for determining metastatic risk in melanoma patients. *Diagn Pathol.* Feb 13, 2018;13(1):13.<br><br>*Country:* USA	*Source:*<br>This study was sponsored by Castle Biosciences<br><br>7 authors: employees of Castle Biosciences<br><br>7 authors: hold stock in the company	*Methodology:*<br><br>Lab validation study<br><br>Inter-assay, inter-instrument, and inter-operator studies to evaluate reliability of test results, sample stability and reagent stability.	*Stated Objective:*<br>Assess reproducibility of Decision DX assay<br><br>☐Prospective<br>☒Retrospective<br><br>*Study Population and Setting:*<br><br>*N:* 8244 samples run (March 2013-June 2016)<br>168 samples run twice to assess reproducibility<br><br>168 specimens (de-identified of patient info)<br><br>*Intervention:* none<br><br>*Outcome Measures:*<br>Reproducibility of Decision Dx assay<br><br>*Follow-Up:*<br><br>*Notes:* Based on existing fixed tissue samples	*Results:*<br>Among all samples, the technical success of the assay was 98% in the 85% of samples that met pre-specified tumor content parameters.<br><br>Concordance among assays (in 168 subset of samples for which repeat assay was performed) on two consecutive days was 99%. Inter-instrumental concordance was 95%.<br><br>Analytic validity based on inter-assay, inter-operator, and inter-instrument reliability measurements show technical success of the test (99% rate).<br><br>*Notes:*	*Describe conclusions relative to question:*<br><br>The assay is reliable and reproduceable.<br><br>*Critiques of Methodology:*

	Summary of Evidence Table<br>Question 1, 2, 3					
Level of Evidence*^<br><br>Choose one:	Study Citation<br>Author(s), Year, Title, Journal, Volume, Page #s	Funding Source	Study Methodology	Objective & Methods	Results	Conclusions
Strong<br>☐EL 1; RCT<br>☐EL 1; MRCT<br><br>Intermediate<br>☐EL 2; MNRCT<br>☐EL 2; NMA<br>☐EL 2; NRCT<br>☐EL 2; PCS<br>☐EL 2; RCCS<br>☐EL 2; NCCS<br>☐EL 2; CSS<br>☐EL 2; ES<br>☐EL 2; OLES<br>☐EL 2; PHAS<br><br>Weak<br>☐EL 3; DS<br>☐EL 3; ECON<br>☐EL 3; CCS<br>☐EL 3; SCR<br>☐EL 3; PRECLIN<br>☐EL 3; BR<br><br>No Evidence<br>☒EL 4; NE<br>☐EL 4: O	Farberg AS, Marson JW, Glazer A, et al. Expert Consensus on the Use of Prognostic Gene Expression Profiling Tests for the Management of Cutaneous Melanoma: Consensus from the Skin Cancer Prevention Working Group. *Dermatol Ther.* 2022;12(4):807-823.<br>(81)<br><br>*Country:* USA	*Source:*<br>No funding or sponsorship was received for this study or publication of this article.<br><br>3 authors=consultants for Castle Biosciences<br><br>1 author=advisory board member and speaker for Castle Biosciences	*Methodology:*<br>Modified Delphi technique for consensus statements.<br><br>Review – no new data.<br><br>Skin Cancer Prevention Working Group are authors.	*Stated Objective:*<br>Assess applications for GEP in melanoma management.<br><br>☐Prospective<br>☐Retrospective<br><br>*Study Population and Setting:*<br><br>*N:*<br><br>*Intervention:*<br><br>*Outcome Measures:*<br><br>*Follow-Up:*<br><br>Notes:	*Results:*<br>Consensus statements support role for GEP above and beyond AJCC and NCCN.<br><br>*Notes:*<br>Consensus statements support role for GEP above and beyond AJCC and NCCN.<br>The statements are non-specific.	*Describe conclusions relative to question:*<br><br>Consensus statements support role for GEP.<br><br>"GEP tests provide additional, reproducible information for dermatologists to consider within the larger framework of the eighth edition of the AJCC and NCCN cutaneous melanoma guidelines when counseling regarding prognosis and when considering a sentinel lymph node biopsy."<br><br>*Critiques of Methodology:*<br>No formal accompanying systematic review/meta-analysis to support the consensus

						process (only 8 panelists).<br><br>Review of existing data.

	Summary of Evidence Table<br>Question 2,3					
Level of Evidence*^<br><br>Choose one:	Study Citation<br>Author(s), Year, Title, Journal, Volume, Page #s	Funding Source	Study Methodology	Objective & Methods	Results	Conclusions
Strong<br>☐EL 1; RCT<br>☐EL 1; MRCT<br><br>Intermediate<br>☐EL 2; MNRCT<br>☐EL 2; NMA<br>☐EL 2; NRCT<br>☐EL 2; PCS<br>☐EL 2; RCCS<br>☐EL 2; NCCS<br>☐EL 2; CSS<br>☐EL 2; ES<br>☐EL 2; OLES<br>☐EL 2; PHAS<br><br>Weak<br>☐EL 3; DS<br>☐EL 3; ECON<br>☒EL 3; CCS<br>☐EL 3; SCR<br>☐EL 3; PRECLIN<br>☐EL 3; BR<br><br>No Evidence<br>☐EL 4; NE<br>☐EL 4: O	Ferris LK, Farberg AS, Middlebrook B, et al. Identification of high-risk cutaneous melanoma tumors is improved when combining the online American Joint Committee on Cancer Individualized Melanoma Patient Outcome Prediction Tool with a 31-gene expression profile-based classification. *J Am Acad Dermatol*. May 2017;76(5):818-825.e3.<br><br>*Country:* USA	*Source:* Funded by Castle Biosciences which provided financial compensation to those centers contributing cutaneous melanoma tissue to the study.<br><br>6 authors: employees of Castle Biosciences<br><br>4 authors: served as consultants to Castle Bioscience	*Methodology:* RNA isolated to run 31 gene test.<br><br>Five-year survival for each patient using AJCC Individualized Melanoma Patient Outcome Prediction Tool<br>Survival cutoff for low and high-risk determined by stage IIA (79%) and stage IIB (68%) cohorts.<br><br>K-M analysis and Cox proportional hazards survival analysis performed.<br><br>205 cases of stage I/II CM with available tissue were collected from six US centers.	*Stated Objective:* To compare accuracy of GEP with risk determined using the web-based AJCC Individualized Melanoma Patient Outcome Prediction Tool.<br><br>Compare recurrence risk prediction by AJCC Stage vs. 31-GEP class.<br><br>☐Prospective<br>☒Retrospective<br><br>*Study Population and Setting:* Stage I/II CM. 205 retrospectively collected.<br><br>*N:* 205<br><br>*Intervention:* GEP and AJCC prediction tool<br>GEP vs conventional staging<br><br>*Outcome Measures:* RFS, DMFS, OS<br><br>*Follow-Up:* 6.9 y<br>f/u 6.9 years (0.1-15.4)<br><br>*Notes:*	*Results:* 43 (21%) cases had discordant GEP and AJCC classification (with 79% cutoff). 11/43 discordant cases classified as high risk GEP but low by AJCC.<br><br>Sensitivity GEP 82%, 81% and 78% for RFS, DMFS and MSS. AJCC 70%, 69% and 60%.<br><br>Specificity<br>GEP 77%, 69%, 69% and AJCC 83%, 76%, 74%<br><br>Increased sensitivity in combining GEP + AJCC with decreased specificity.<br><br>Multivariate cox regression GEP w AJCC tool indicated GEP more significantly associated with DM and death than binary AJCC.<br><br>HR GEP vs AJCC 79%<br>Recurrence- 5.9 v 3.6<br>DMFS- 5.3 v 3.0,<br>Death -5.3 vs 2.2<br><br>Use of GEP test with AJCC stage improved sensitivity to detect recurrence and to a lesser degree death at the cost of specificity compared with AJCC alone.	*Describe conclusions relative to question:*<br><br>To combine GEP with AJCC may help identify pt benefit from increased surveillance and administer therapies.<br><br>GEP can improve detection of recurrence/mets when used in combination with AJCC.<br><br>Somewhat overstated based on limitations of dataset.<br><br>*Critiques of Methodology:* Using AJCC tool as binary high-risk vs low-risk to fit comparison.<br><br>Unconventional and narrow to define sensitivity and specificity of test using definition of

					______________________<br>Notes:	low-risk group from stage IIA AJCC 79% survival and high-risk 68% survival based off of stage IIB.<br><br>These patients were previously published in the development of the model. Unclear how this subset was selected. Stage was dichotomized at IIA- vs IIB+ somewhat arbitrarily.

Level of Evidence*^ Choose one:	Summary of Evidence Table Question 1,2,3 Study Citation Author(s), Year, Title, Journal, Volume, Page #s	Funding Source	Study Methodology	Objective & Methods	Results	Conclusions
☐EL 1; RCT<br>☐EL 1; MRCT<br>Strong<br><br>☐EL 2; MNRCT<br>☐EL 2; NMA<br>☐EL 2; NRCT<br>☐EL 2; PCS<br>☐EL 2; RCCS<br>☐EL 2; NCCS<br>☐EL 2; CSS<br>☐EL 2; ES<br>☐EL 2; OLES<br>☐EL 2; PHAS<br>Intermediate<br><br>☐EL 3; DS<br>☐EL 3; ECON<br>☐EL 3; CCS<br>☐EL 3; SCR<br>☐EL 3; PRECLIN<br>☐EL 3; BR<br>Weak<br><br>☒EL 4; NE<br>☐EL 4: O<br>No	Grossman D, Okwundu N, Bartlett EK, et al. Prognostic Gene Expression Profiling in Cutaneous Melanoma: Identifying the Knowledge Gaps and Assessing the Clinical Benefit. *JAMA Dermatol*. 2020;156(9):1004-1011.<br>(61)<br><br>*Country:* United States	*Source:* Supported by the University of Utah Department of Dermatology, the Huntsman Cancer Foundation at the University of Utah, the Melanoma Center at the Huntsman Cancer Institute, the Hope Foundation, the National Health and Medical Research Council of Australia Program, fellowship grants and grants from the American Skin Association and the Sydney Medical School Foundation, This material is the result of work supported	*Methodology:*<br><br>Consensus statement	*Stated Objective:* Develop guidelines within the national Melanoma Prevention Working Group (MPWG) on integration of GEP testing into management of patients with CM.<br>9. Review of published data using GEP tests<br>10. Definition of acceptable performance criteria<br>11. Current recommendations of use of GEP in clinical practice<br>12. Considerations for future studies<br><br>☐Prospective<br>☒Retrospective<br><br>Study Population and Setting:<br><br>N: N/A<br><br>Intervention: Consensus guideline<br><br>MPWG members and other international specialists, 2 surveys<br><br>Outcome Measures: N/A	*Results:*<br><br>The MPWG members are optimistic about the future use of prognostic GEP testing to improve risk stratification and enhance clinical decision-making but acknowledge that current utility is limited by test performance in patients with stage I disease. Published studies of GEP testing have not evaluated results in the context of all relevant clinicopathologic factors or as predictors of regional nodal metastasis to replace sentinel lymph node biopsy (SLNB). The performance of GEP tests has generally been reported for small groups of patients representing particular tumor stages or in aggregate form, such that stage-specific performance cannot be ascertained, and without survival outcomes compared with data from the American Joint Committee on Cancer 8th edition melanoma staging system international database. There are significant challenges to performing clinical trials incorporating GEP testing	*Describe conclusions relative to question:* Enthusiasm for the concept, but limited test performance in stage I disease. Published studies not evaluated results in context of all relevant clinicopathologic factors or as predictors of regional nodal metastasis to replace SLNB.<br><br>More studies are needed to determine clinical utility of GEP.<br><br>*Critiques of Methodology:*<br><br>Consensus guideline was performed reasonably.

		with resources and use of facilities at the Veterans Affairs Palo Alto Health Care System in Palo Alto CA. Contents do not represent the views of the US Dept of Veterans Affairs or the US Gov't. The funders had no role in the design and conduct of the study; collection, management, analysis and interpretation of data, preparation, review or approval of the manuscript; and decision to submit for publication.<br><br>1 author: nonfinancial support from Castle Biosciences outside submitted work.<br><br>1 author: grants from Castle Biosciences outside		Follow-Up:<br><br>Notes:	with SLNB and adjuvant therapy. The MPWG members favor conducting retrospective studies that evaluate multiple GEP testing platforms on fully annotated archived samples before embarking on costly prospective studies and recommend avoiding routine use of GEP testing to direct patient management until prospective studies support their clinical utility.<br><br>Notes:	

		submitted work<br><br>1 author: personal fees outside the submitted work Castle Biosciences<br><br>1 author: personal fees from Neracare outside submitted work<br><br>1 author: manuscripts and abstracts published using the test with company support of the assay—all publications were peer review and no personal of institutional payment or compensation was rec'd. Castle Biosciences<br><br>1 author: served as an investigator for Castle Biosciences (no personal financial compensation)				

	Summary of Evidence Table<br>Question 1, 2, 3					
Level of Evidence*^<br><br>Choose one:	Study Citation<br>Author(s), Year, Title, Journal, Volume, Page #s	Funding Source	Study Methodology	Objective & Methods	Results	Conclusions
**Strong**<br>☐EL 1; RCT<br>☐EL 1; MRCT<br><br>**Intermediate**<br>☐EL 2; MNRCT<br>☐EL 2; NMA<br>☐EL 2; NRCT<br>☐EL 2; PCS<br>☐EL 2; RCCS<br>☐EL 2; NCCS<br>☐EL 2; CSS<br>☐EL 2; ES<br>☐EL 2; OLES<br>☐EL 2; PHAS<br><br>**Weak**<br>☐EL 3; DS<br>☐EL 3; ECON<br>☐EL 3; CCS<br>☐EL 3; SCR<br>☐EL 3; PRECLIN<br>☐EL 3; BR<br><br>**No Evidence**<br>☒EL 4; NE<br>☐EL 4: O	Svoboda RM, Glazer AM, Farberg AS, Rigel DS. Factors Affecting Dermatologists' Use of a 31-Gene Expression Profiling Test as an Adjunct for Predicting Metastatic Risk in Cutaneous Melanoma. J. Drugs Dermatol. 2018;17(5):544-547. (35)<br><br>*Country:*<br>USA	*Source:*<br><br>This study was funded in part by a grant from Castle Biosciences Inc.<br><br>1 author: consultant to Castle Bioscience<br><br>1 author: served on advisory board for Castle Bioscience<br><br>2 authors: participated in research fellowship that was partially funded by Castle Bioscience	*Methodology:*<br><br>Dermatologists answered questionnaire with four clinical vignettes to determine the impact of Breslow thickness, ulceration and SLNB status decision to order GEP test.<br><br>Survey-based study performed at a conference.	*Stated Objective:*<br>Clinical factors that impact dermatologists' decisions to utilize 31-GEP.<br><br>☒Prospective<br>☐Retrospective<br><br>*Study Population and Setting*:<br>Dermatology questionnaire at a national conference.<br><br>Panel of clinicians.<br>Survey<br><br>*N:* 181/187<br><br>*Intervention:*<br>Questionnaire with four clinical vignettes.<br><br>Opinion regarding management<br><br>*Outcome Measures:*<br>Percentage of respondents who would order 31-GEP in clinical scenarios.<br><br>Recommended treatment pathway.<br><br>*Follow-Up*: N/A<br><br>*Notes:*	*Results:*<br>A majority of patients would recommend GEP test."<br><br>Breslow thickness ≥ 0.5 mm, majority dermatologists would order GEP. Ulceration was associated with a statistically significant increase to recommend for all but the thickest ≥ 2.1 mm. For thin tumor (0.26 mm) ulceration significantly changed from 22% to 67%, p <0.001). A negative SLNBx only associated with statistically significant increase in the percentage for the thinnest tumors (22% to 34%, p=0.033).<br><br>Impact of GEP test result on T1b 0.76-1.0 mm melanoma:<br>Class 1 result -91% respondents reported less likely to recommend SLNB.<br>Class 2 - 81% would make more likely to recommend SLNB.<br><br>*Notes:*	*Describe conclusions relative to question:*<br>Not relevant to current clinical practice.<br><br>Ulceration most important factor deciding to order 31-GEP.<br><br>*Critiques of Methodology:*<br>Authors do not state whether the survey was taken at a session paid for by Castle Bioscience.<br><br>Authors have conflict of interest – was the survey voluntary<br><br>-Survey of dermatologists' ordering pattern does not evaluate the validity of the test.<br>- Selection bias in those willing to take the survey

						-was survey voluntary or was there an incentive.

	Summary of Evidence Table<br>Question 1, 2, 3					
Level of Evidence*^<br><br>Choose one:	Study Citation<br>Author(s), Year, Title, Journal, Volume, Page #s	Funding Source	Study Methodology	Objective & Methods	Results	Conclusions
Strong<br>☐EL 1; RCT<br>☐EL 1; MRCT<br><br>Intermediate<br>☐EL 2; MNRCT<br>☐EL 2; NMA<br>☐EL 2; NRCT<br>☐EL 2; PCS<br>☐EL 2; RCCS<br>☐EL 2; NCCS<br>☐EL 2; CSS<br>☐EL 2; ES<br>☐EL 2; OLES<br>☐EL 2; PHAS<br><br>Weak<br>☐EL 3; DS<br>☐EL 3; ECON<br>☐EL 3; CCS<br>☐EL 3; SCR<br>☐EL 3; PRECLIN<br>☐EL 3; BR<br><br>No Evidence<br>☒EL 4; NE<br>☐EL 4: O	Swetter SM, Tsao H., Bichakjian CK, Curiel-Lewandrowski C, Ender DE, Gershewald JE, et al. Guidelines of Care for the Management of Primary Cutaneous Melanoma. *J. Am. Acad. Dermatol.* 2019 Jan 1; 80(1):208-250. (117)<br><br>*Country:* USA	*Source:* None<br><br>1 author: served as an advisory board member to Castle Biosciences, receiving fees.	*Methodology*: Practice guideline development.<br><br>AAD guidelines for the management of primary cutaneous melanoma from a group of dermatologists, patient advocate, at least one surgeon<br><br>2018	*Stated Objective:*<br><br>*☐Prospective*<br>*☐Retrospective*<br><br>*Study Population and Setting:*<br><br>*N:*<br><br>*Intervention:*<br><br>*Outcome Measures:*<br><br>*Follow-Up:*<br><br>*Notes:*	*Results:*<br><br>GEP testing can stratify patients into risk categories with some degree of precision; however, the data to date consists of heterogenous groups of high risk event enriched groups that may not represent the spectrum of melanoma patient population and the clinical applicability of the tests is uncertain based on the lack of surgical or therapeutic RCTS to examine outcomes with GEP testing.<br><br>Page 230 of the article addresses GEP and cautions against routine use for clinical decision-making pending appropriate clinical trials evaluating clinical usefulness of tests and whether currently available tests add to AJCC 8th edition staging.<br><br>*Notes:*	*Describe conclusions relative to question:*<br><br>The working group discourages baseline GEP testing for prognostication and for management decisions.<br><br>AAD opinion on GEP provided on p 230.<br><br>*Critiques of Methodology:*
